# CO_2_ Reduction to Formic Acid/Formate by Intermittent Electricity at Bismuth Gas Diffusion Electrodes

**DOI:** 10.1002/cssc.202501583

**Published:** 2025-09-30

**Authors:** Ida Dinges, Siegfried R. Waldvogel, Markus Stöckl

**Affiliations:** ^1^ Chemical Technology DECHEMA Research Institute Theodor‐Heuss‐Allee 25 60486 Frankfurt am Main Germany; ^2^ Department for Electrosynthesis Max‐Planck‐Institute for Chemical Energy Conversion Stiftstraße 34‐36 45470 Mülheim an der Ruhr Germany; ^3^ Karlsruhe Institute of Technology Institute of Biological and Chemical Systems – Functional Molecular Systems (IBCS‐FMS) Kaiserstraße 12 76131 Karlsruhe Germany

**Keywords:** bismuth, cathode, CO_2_ valorization, flexible electricity consumption, formic acid

## Abstract

To avoid the waste of renewable energy resources beyond demand and grid capacity, innovative gas diffusion electrodes (GDE) for operation at intermittent electricity are presented. They are based on Bi as affordable and nontoxic electrocatalyst, to facilitate decentralized and cost‐efficient reduction of CO_2_ to formic acid. To develop flexible GDE materials, their catalyst composition is optimized by studying systematically inexpensive Bi/Bi_2_O_3_ mixtures. During initial evaluation at technically relevant current density (150 mA cm^−2^, 21 h), the best composition achieve high Faradaic efficiency (FE) (≈90%) and the loss of catalyst is minor. In three demonstrative examples of realistic current patterns based on intermittent electricity, the performance and resilience of the optimized GDE is consistently very good in terms of high FE (≈90%) and stable synthesis rates of formate. However, loss of catalyst is partially increased, especially when GDEs are depolarized between electrolysis phases. Nonetheless, the GDE materials already show robust performances despite swift adjustments of current density (60 s) without any optimization of operational parameters so far. Based on these results, flexible operation of these GDE can be optimized to minimize cathodic corrosion of catalyst at long‐term operation, and thus, ultimately evaluate their implementation to valorize intermittent electricity.

## Graphical abstract

To use intermittent electricity for CO_2_ reduction to formic acid/formate, gas diffusion electrodes (GDE) are developed for flexible operation at variable current density. They are based on Bi as affordable electrocatalyst to facilitate decentralized implementation. The cost‐efficient GDE show remarkably robust performance despite swift adjustment of current density without parameter optimization.

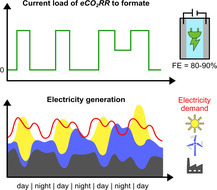

## 1 Introduction

Driven by anthropogenic climate change, the chemical industry needs a transition to renewable energy sources and sustainable feedstocks. Two of the most important renewable energy sources are solar and wind energy, but they only provide intermittent electricity depending on day‐night cycles and/or weather conditions. Furthermore, electricity generation based on solar and wind power can exceed demand as well as grid and storage capacity. Therefore, photovoltaics and/or wind turbines have frequently to be taken off the grid, wasting potential energy resources instead of using this surplus of energy. Of course, progress has been made to improve energy storage (e.g., battery systems) and to increase storage capacities in general. However, another attractive option would be to use the fluctuating excess energy with flexible electrosynthesis systems, instead of shutting off renewable energy sources in order to stabilize the grid. This is also in line with the strategy of electrifying chemical processes.^[^ ^1^, ^2^, ^3^ ^]^ Furthermore, electrosynthesis can be carried out decentralized at point sources of resources (e.g., local CO_2_ emitters) and/or on‐site on demand. This offers the unique and attractive opportunity to exploit intermittent electricity directly to generate feedstocks for the chemical industry, which would support its defossilization. To facilitate this, unit capital costs of systems for decentralized on‐site applications should be as low as possible to avoid high investment barriers.

To purse this approach, electrochemical reduction of CO_2_ is particularly promising, as it works at ambient conditions and would integrate CO_2_ as sustainable carbon source toward a circular carbon economy. In addition, several point sources (e.g., flue gas) are available and their use would mitigate CO_2_ emissions while avoiding the high costs of direct air capture.^[^ ^4^ ^]^ Among numerous possible reduction products,^[^ ^5^ ^]^ formic acid and formate are attractive due to their general importance in various areas, such as textile and leather industries, as preservatives and antibacterial agents and for biological/medical research.^[^ ^6^, ^7^ ^]^ Furthermore, their electrosynthesis is compelling due to their good storage and transport features, as well as their versatile subsequent applications (e.g., hydrogen source,^[^ ^8^, ^9^ ^]^ substrate for biosynthesis,^[^ ^10^, ^11^, ^12^ ^]^ starting material for performic acid^[^ ^13^, ^14^ ^]^). Moreover, electro‐synthesized formic acid/formate is considered to become economically viable in the near future.^[^ ^15^, ^16^ ^]^ For efficient conversion of CO_2_ at high current density, suitable electrocatalysts are employed with gas diffusion electrodes (GDE) circumventing the low solubility of CO_2_ in aqueous electrolytes (33 mmol L^−1^ at 298 K and 1 atm).^[^ ^17^, ^18^ ^]^ Of several cathodic electrocatalysts selective for formic acid formation, Bi has received increasing attention in numerous variations (e.g., nanoparticles, nanosheets, flakes, dendrites) in recent years.^[^ ^19^, ^20^, ^21^ ^]^ Besides their selectivity, Bi‐based electrocatalysts are promising because Bi is an affordable metal (95 € kg^−1^ standard ingot 15 kg)^[^ ^22^ ^]^ and is considered nontoxic.^[^ ^23^ ^]^ As Bi mainly occurs in small amounts in various ores and minerals, it is co‐obtained as byproduct of commodity metals (e.g., W, Pb, Zn).^[^ ^24^, ^25^ ^]^ Global reserves were estimated at 370,000 t in 2017,^[^ ^26^ ^]^ but Bi is often unreported in the prospection of ores and little data is reported. Consequently, more Bi seems to be available.^[^ ^24^ ^]^ For some years now, the global production for Bi has been dominated by China and Vietnam.^[^ ^27^ ^]^ For example, the European Union imported over 90% of Bi from China in 2024.^[^ ^28^ ^]^ Since the production and export shares of Bi are highly concentrated, this metal is recognized as critical raw material by the European Commission, the United Kingdom, the United States, India, and Australia.^[^ ^24^, ^25^ ^]^ Nonetheless, there are Bi natural resources on every continent, and thus, sufficient reserves in case of geopolitical uncertainties.

So far, great efforts have already been made in the development of Bi‐based catalysts to enable efficient CO_2_ reduction to formic acid/formate at high current density (0.5–2 A cm^−2^) with high Faradaic efficiency (FE) (≥90%)^[^ ^29^, ^30^, ^31^, ^32^ ^]^ and toward continuous production.^[^ ^33^ ^]^ Elaborate catalysts with finely tailored catalytic sides, facets, and grain boundaries have been developed.^[^ ^34^, ^35^, ^36^ ^]^ Thereby, complex engineering of catalyst materials generally increases costs, which would increase unit costs for on‐site, decentralized electrosynthesis. Moreover, most elaborate catalysts and electrode materials are developed for continuous operation at constant high current density, neglecting material stability under intermittent electricity/variable current density. However, the latter is prerequisite for the translation into application.

With this in mind, this study concerns the development of cost‐efficient, mechanically durable, and chemically stable Bi‐based GDEs for flexible operation at intermittent electricity. GDEs were developed systematically varying Bi to Bi_2_O_3_ ratio. Thereby, elemental Bi was selected because it is the primary version of Bi by refining. In addition, Bi_2_O_3_ was chosen as precursor (i.e., pore‐forming agent) to enhance electrode material performance.^[^ ^34^, ^35^, ^36^ ^]^ Details on design strategy are outlined below. As a result, GDEs with an optimized Bi/Bi_2_O_3_ composition were obtained and subsequently used in flexible electrolysis. Different realistic intermittent electricity scenarios were applied and the efficient and stable electrosynthesis of formate at high FE (≈90%) was successfully demonstrated at such variable current density.

## 2 Results and Discussion

To identify the best catalyst composition of Bi/Bi_2_O_3_‐based GDEs for electrosynthesis of formate at variable current load, six different compositions were systematically investigated. All fabricated GDEs were first applied for electrosynthesis of formate at constant current density (150 mA cm^−2^, 21 h) and characterized prior and upon electrolysis. The current density was based on the results of comparable previous studies.^[^ ^12^, ^14^ ^]^ Subsequently, the best performing GDE was applied for variable CO_2_ reduction under various current conditions.

### 2.1 Fabrication and Characterization of GDE

For GDE design strategy, elemental Bi was selected because it is the main product of global Bi production/refining. Bi_2_O_3_ was added to it systematically as precatalyst and to act as reductive binder, as it is reduced to Bi during cathodic polarization.^[^ ^14^ ^]^ Thereby, it will increase the conductivity of the Bi‐based GDE by improving interconnectivity of the individual particles, and thus, lower the operational energy demand. This was based on the well‐established silver/silver oxide based GDE in chlor‐alkali electrolysis.^[^ ^37^, ^38^ ^]^ Furthermore, Bi_2_O_3_ likely acts as a pore‐forming agent upon reduction,^[^ ^39^ ^]^ which could also improve mass transport within the GDE. Moreover, Ni foam was chosen as porous support material instead of gas diffusion layers based on carbon, as these are vulnerable to degradation and thus flooding of the GDE.^[^ ^40^ ^]^

The full catalyst GDEs were fabricated by heat pressing Bi/Bi_2_O_3_ onto Ni foam as support material and current collector. This was based on the oxygen depolarized cathode technology developed by Covestro.^[^ ^37^ ^]^ Each catalyst mixture had the same electrocatalyst to binder weight ratio (87.5:12.5). As relatively inexpensive electrocatalysts, Bi (particle size ≤ 40 μm, ≈50 € kg^−1^) and/or Bi_2_O_3_ (particle size ≈80 nm, ≈200 € kg^−1^) were used. Polytetrafluoroethylene (PTFE) powder served as hydrophobic binder (≈50 € kg^−1^). All materials were used as commercially available (Supporting Information).

In total, three GDEs (*n* = 3) were fabricated for each of the six different compositions of electrocatalyst (Bi/Bi_2_O_3_). The composition was systemically varied in steps of 20 wt% from pure Bi (dark gray) to pure Bi_2_O_3_ (yellow). The catalyst loading *b* (67 ± 3 mg cm^−2^, Table 2) and thickness *d* (507 ± 24 μm) of all GDEs were in the same range. Furthermore, no influence of minor variations of the predominantly manual steps of the fabrication method (e.g., distribution of catalyst mixture, handling of the GDE blank, cf. Supporting Information) on GDE performance was observed.

**Figure 1:**
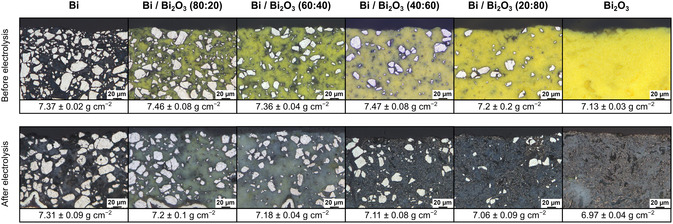
Cross section microscopy image of the catalyst layer and density of fabricated GDE with varying composition for electrosynthesis of formate before and after electrolysis.

Taking contemporary costs into account, electrocatalyst cost of GDEs increased from 34.3 ± 0.1 € m^−2^ (pure Bi) to 130.5 ± 0.5 € m^−2^ (pure Bi_2_O_3_). All GDE types were characterized before and after electrolysis (21 h at 150 mA cm^−2^) by bright‐field microscopy of cross sections and measurement of density (**Figure** **1**, cf. Supporting Information).

The microscopy images show the distribution of Bi particles (light color due to reflection) with various sizes (≤40 μm) in PTFE binder and/or Bi_2_O_3_. As Bi_2_O_3_ particles were a magnitude smaller (≈80 nm), they cannot be distinguished at this resolution. However, areas rich in Bi_2_O_3_ of the mixed GDE as well as the pure Bi_2_O_3_ GDE appear relatively homogeneous prior to electrolysis. After electrolysis, most Bi_2_O_3_ (yellow) was reduced to Bi (dark gray).^[^ ^14^ ^]^ The nontransparent white binder is visible as cloudy shadow. Noticeably, Bi particles seemed more porous upon electrolysis for each composition, which might indicate cathodic corrosion. Furthermore, some yellow (not reduced) Bi_2_O_3_ is still visible. This indicates some Bi_2_O_3_ could have been encapsulated in binder and was not accessible for electrolysis. Furthermore, different shades of gray in the catalyst layer indicate a small degree of inhomogeneity.

To examine stability and electrochemically induced changes of the GDE further, their density was determined with a gas pycnometer. In general, density was up to 4.9% lower after electrolysis. By reduction of Bi_2_O_3_ to Bi, a small decrease in density was expected. But unexpectedly, the GDE with the highest Bi_2_O_3_ content did not show the largest decrease in density (2.3%). Since density measurements after electrolysis were conducted with simply air‐dried GDE, salt precipitations inside the porous GDE might have increased measured density. Furthermore, density could also have been decreased by loss of Bi due to cathodic corrosion. Hence, electrolytes were examined with inductively coupled plasma optical emission spectroscopy (ICP‐OES) after electrolysis.

ICP‐OES analysis revealed low concentrations of dissolved Bi and Ni in the catholyte for all catalyst compositions (**Table** **1**).

**Table 1:** Concentrations of Bi^3+^ and Ni^2+^ in the catholyte after electrosynthesis of formate (*n* = 3) determined via ICP‐OES.

Catalyst composition of GDE	c(Bi^3+^) [μg L^−1^]	c(Ni^2+^) [μg L^−1^]
Bi	342 ± 212a)	25 ± 6
Bi/Bi_2_O_3_ (80:20)	109 ± 32a)	20 ± 3
Bi/Bi_2_O_3_ (60:40)	101 ± 9	55 ± 7
Bi/Bi_2_O_3_ (40:60)	117 ± 13	60 ± 16
Bi/Bi_2_O_3_ (20:80)	86 ± 9	89 ± 20
Bi_2_O_3_	112 ± 14	52 ± 5

a) (*n* = 2), excluding outlier (see Supporting Information).

Noticeably, the highest concentration of Bi occurred for GDE with pure Bi as starting material for the electrocatalyst. This agrees with observed cavities in the Bi particles, which made them seem more porous as described above. Consequently, pure Bi particles seemed more affected by cathodic corrosion than Bi originating from the reduction of Bi_2_O_3_. Furthermore, comparison of Bi/Bi_2_O_3_ (40:60) and Bi_2_O_3_ GDE show their cathodic corrosion of Bi was similar and therefore cannot explain the difference in density after electrolysis (4.9% vs. 2.3%). This suggests GDE densities may have been altered by salt precipitates in the GDE pore structures after electrolysis, as mentioned above.

Besides Bi, low concentrations of Ni were found in the catholyte for all catalyst compositions. This shows the GDE's support material Ni foam was in contact with catholyte and was slightly affected by cathodic corrosion, respectively. Noticeably, the highest concentration of Ni (Bi/Bi_2_O_3_, 20:80 GDE) did not coincide with the highest concentration of Bi (pure Bi GDE).

Nonetheless, the overall concentrations of Bi and Ni were rather low. Hence, no catalyst composition was significantly affected by cathodic corrosion and the respective catalyst was rather stable at the electrolysis conditions examined (21 h at 150 mA cm^−2^). The GDEs’ lifespan beyond the presented runtimes were not evaluated.

To summarize, density and cathodic corrosion of the six types of GDE were altogether similar, although their cross sections before and after electrolysis showed significant differences. Accordingly, greater differences in performance were expected during electrolysis. The electrolysis results are discussed in the following chapter to evaluate GDE performance.

### 2.2 Electrosynthesis of Formate at Constant Current Density

Electrosynthesis of formate was carried out in a gas‐fed flow reactor (5 cm^2^ GDE, anolyte, and catholyte were circulated and separated by a cation exchange membrane, Section S1.3, Supporting Information) using 0.2 mol L^−1^ KH_2_PO_4_/K_2_HPO_4_ (equimolar, pH ≈ 6.67) as both anolyte and catholyte. Phosphate buffer was chosen as supporting electrolyte based on promising results in previous studies.^[^ ^11^, ^12^, ^14^ ^]^ To evaluate performance of the six different GDE types, electrolyses were run 21 h at constant current density of 150 mA cm^−2^ (*n* = 3, respectively). The aim was to achieve and maintain high FE (>85%) and stable synthesis rates for formate. Catholyte samples were taken every hour to determine concentration and FE of formate and additionally every 15 min in two intervals to determine formate synthesis rates. The first interval (4–5 h) was after initial conditioning of the GDE to ensure electrochemical reduction of Bi_2_O_3_ was complete and electrowetting of the GDE was relatively stable. The second interval (20–21 h) was in the last hour of the electrolysis to assess stability of synthesis rates. Detailed courses of the individual electrolysis are provided in the Supporting Information Section S3.1. By screening the catalyst composition as described above, the least expensive composition for efficient electrosynthesis of formate was determined. The results of the six GDE types are summarized and discussed below.

**Figure 2:**
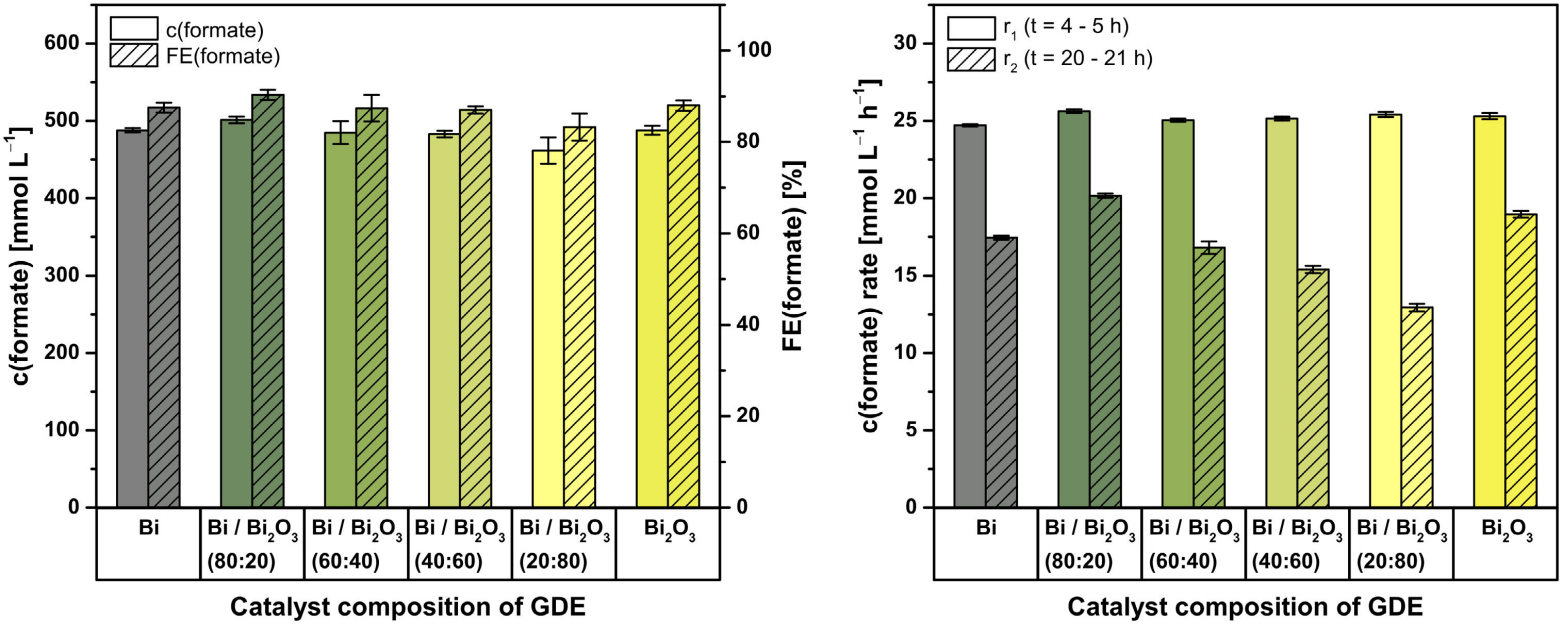
Final concentration and corresponding FE of formate (right) and formate synthesis rates (left) for the six different catalyst compositions of GDE (*n* = 3, respectively). Electrolysis parameters: Constant current density *j* = 150 mA cm^−2^, runtime = 21 h (≙ 56 700 C), electrolyte = 0.2 mol L^−1^ KH_2_PO_4_/K_2_HPO_4_, initial *V* (catholyte, anolyte) = 500 mL each, membrane = Nafion 424, cathode (GDE, 5 cm^2^) = 87.5 wt% Bi and/or Bi_2_O_3_ (composition specified in graph), 12.5 wt% PTFE on Ni foam, reference electrode = RHE, anode = mixed Ir‐oxide on a Ti‐grid (Platinode EP, Type 177, Umicore).

**Figure** **2** depicts an overview of total concentration and FE after electrolysis and synthesis rates of formate (4–5 and 20–21 h, respectively) for the six different GDE types.

Generally, final concentration of formate was 484 ± 13 mmol L^−1^ for all different GDEs (*n* = 18), which was accompanied by corresponding FE of 87.3 ± 2.3%. Despite significant differences in characteristics before electrolysis (Figure 1), GDEs based on pure Bi (488 ± 3 mmol L^−1^, 87.5 ± 1.1%) performed just as well as those based on pure Bi_2_O_3_ (488 ± 6 mmol L^−1^, 88.0 ± 1.2%). However, mixtures of Bi and Bi_2_O_3_ yielded slightly different results. Thereby, no uniform trend or correlation of concentration and FE with the systematic change in composition was discernible. Of the four mixed catalyst compositions, three yielded lower concentrations, and thus, FE than pure Bi or Bi_2_O_3_ GDEs. Furthermore, results of Bi/Bi_2_O_3_ (60:40) and Bi/Bi_2_O_3_ (20:80) GDEs had relatively high standard deviations. This comparatively lower reproducibility could be due to the lower mechanical stability of these GDE compositions.

In contrast, Bi/Bi_2_O_3_ (80:20) GDEs achieved the best results in terms of total concentration (501 ± 5 mmol L^−1^) and FE (90.3 ± 1.2%) of formate. In all instances, FE balance was most likely closed by the parasitic hydrogen evolution reaction (HER) as main side reaction, which is also favored by the increasingly acidic pH during electrolysis.^[^ ^12^, ^14^ ^]^

To evaluate performance stability of the GDEs, synthesis rates of formate were compared after conditioning of the GDE (*t* = 4–5 h) and at the end of electrolysis (*t* = 20–21 h). Generally, synthesis rates of formate after conditioning (r_1_) were relatively similar for all catalyst compositions (25.2 ± 0.4 mmol L^−1^ h^−1^, *n* = 18). Thereby, synthesis rate (r_1_) of Bi GDE was the lowest (24.71 ± 0.09 mmol L^−1^ h^−1^), whereas Bi/Bi_2_O_3_ (80:20) GDE was the highest (25.62 ± 0.12 mmol L^−1^ h^−1^). However, synthesis rates of formate (r_2_) generally declined at the end of electrolysis (16.9 ± 2.6 mmol L^−1^ h^−1^, *n* = 18) and differed significantly depending on the composition of the catalyst. In contrast to similar total concentration and FE of formate, the synthesis rate (r_2_) of GDEs based on pure Bi (17.45 ± 0.14 mmol L^−1^ h^−1^) was slightly lower than the one based on pure Bi_2_O_3_ (19.0 ± 0.3 mmol L^−1^ h^−1^). As before, there was no uniform trend/correlation between synthesis rate (r_2_) and systematic increase of Bi_2_O_3_ in the catalyst composition of the GDEs. Out of all catalyst compositions, Bi/Bi_2_O_3_ (80:20) GDEs (20.16 ± 0.14 mmol L^−1^ h^−1^) had the highest synthesis rate (r_2_) and thus maintained most of its initial performance (79%). The general decline in synthesis rate (r_1_ vs. r_2_) could be attributed to an increased influence of HER during electrolysis. This could be caused by, for example, either presumed formate mass transport limitations within the GDE's pore system or the increasingly acidic pH during electrolysis (Section S3.1, Supporting Information). Moreover, the employed set‐up was optimized for screening and not yet for continuous synthesis of formate with concentrations above 500 mmol L^−1^. However, the strong relative differences of r_2_ could be attributed to presumably different pore structures due to varying content of Bi_2_O_3_ as pore‐forming agent. Thereby, Bi/Bi_2_O_3_ (80:20) seemed to have the best porous structure to allow good mass transport, as r_2_ was highest here. In regard of total concentration, FE and synthesis rates of formate, Bi/Bi_2_O_3_ (80:20) was the most promising composition of catalyst for electrosynthesis of formate.

In addition to FE and synthesis rate, energy demand was also considered to determine the overall most efficient GDE (**Table** **2**). Electrolyses were run with an average cell voltage of 6.46 ± 0.12 V for all different compositions of catalyst (*n* = 18). The generally relatively high cell voltage was mainly caused by ohmic losses in the anode compartment, as a nonzero gap anode (mixed Ir‐oxide on a Ti‐grid) was used for the oxygen evolution reaction as counter reaction of CO_2_ reduction to formate (cf. Supporting Information). Accordingly, the average potential of GDEs was only −1.57 ± 0.14 V versus reversible hydrogen electrode (RHE) for all different compositions of catalyst (*n* = 18). Thereby, stepwise addition of Bi_2_O_3_ as a reductive binder did not lead to the presumed stepwise lowering of GDE potential/increase in conductivity of the GDE (Section S3.1, Supporting Information). Overall, the employed set‐up was not yet optimized for energy efficiency, which offers opportunities to reduce the required cell voltage greatly (e.g., membrane electrode assembly^[^ ^41^, ^42^ ^]^). Therefore, assessment of energy cost was mainly used to compare the different GDEs in relative terms herein. To determine the composition with the highest energy efficiency, electric energy consumption (EEC) for electrosynthesis of formate was calculated (Table S2 and Equation S2, Supporting Information). Generally, EEC of formate was 8.64 ± 0.17 k Wh kg^−1^ for all different compositions of catalyst (*n* = 18), which corresponded to an energy cost for formate of 1.55 ± 0.04 € kg^−1^ (no downstream processing etc., 0.1800 € kWh^−1^^[^ ^43^ ^]^). The assessment revealed Bi/Bi_2_O_3_ (80:20) enabled the most energy efficient (8.37 ± 0.13 k Wh kg^−1^) and thus least expensive (1.51 ± 0.03 € kg^−1^) electrosynthesis of formate. In comparison, all energy costs of formate were higher than market prices for fossil‐based concentrated formic acid, such as 0.37 and 0.69 € kg^−1^ (0.40^[^ ^44^ ^]^ and 0.74 $ kg^−1^,^[^ ^45^ ^]^ respectively with 1 € ≙ 1.08 $). However, they were already in a reasonable order of magnitude.

**Table 2:** Overview of catalyst loading, catalyst cost and energy demand for electrosynthesis of formate at constant current density (150 mA cm^−2^, 21 h) with GDEs of variable catalyst composition (*n* = 3, respectively).

Catalyst composition of GDE	Catalyst loadinga) [mg cm^−2^]	GDE catalysta) cost [€ m^−2^]	Ub) [V]	E(GDE)b) [V]	EEC [kWh kg^−1^]
Bi	68.6 ± 0.2	34.3 ± 0.1	6.63 ± 0.06	−1.7 ± 0.1	8.84 ± 0.05
Bi/Bi_2_O_3_ (80:20)	69 ± 1	55.5 ± 0.1	6.48 ± 0.06	−1.6 ± 0.1	8.37 ± 0.13
Bi/Bi_2_O_3_ (60:40)	70 ± 2	76.6 ± 2.2	6.47 ± 0.01	−1.5 ± 0.1	8.6 ± 0.3
Bi/Bi_2_O_3_ (40:60)	67.1 ± 0.8	94.0 ± 1.1	6.44 ± 0.08	−1.7 ± 0.3	8.63 ± 0.15
Bi/Bi_2_O_3_ (20:80)	63.3 ± 1.1	107.7 ± 1.8	6.3 ± 0.2	−1.37 ± 0.14	8.79 ± 0.34
Bi_2_O_3_	65.3 ± 0.3	130.5 ± 0.5	6.4 ± 0.3	−1.52 ± 0.13	8.55 ± 1.5

a) Bi and/or Bi_2_O_3_;

b) Average of 21 h runtime, without compensation for *iR* losses.

All in all, the variation of catalyst composition demonstrated mixtures of Bi/Bi_2_O_3_ can improve electrosynthesis of formate compared to pure Bi or Bi_2_O_3_ as electrocatalyst. Among the examined compositions, the second least expensive Bi/Bi_2_O_3_ (80:20) GDE was the best in terms of total concentration as well as FE of formate, stability of formate synthesis rate, and energy demand. Additionally, stability of these GDEs was sufficient as cathodic corrosion was minor at the applied conditions, as discussed previously (Table 1). Consequently, it was applied for the following electrolyses at variable current density to finally assess the possible use intermittent electricity.

### 2.3 Electrosynthesis of Formate at Variable Current Density

After identifying Bi/Bi_2_O_3_ (80:20) GDEs as most promising, their operation was investigated at variable current density based on realistic patterns of intermittent electricity (solar and wind power).^[^ ^46^ ^]^ To utilize intermittent electricity with GDEs, they need to be reliably operated at variable current densities while maintaining high FE, and since intermittent electricity fluctuates on a fairly short time scale (≈20 min), GDEs should be robust enough to adjust current density quickly. Ideally, electrosynthesis could be turned on and off repeatedly without compromising GDE performance to accommodate periods of low availability. To examine whether Bi/Bi_2_O_3_ (80:20) GDEs meet these requirements or not, three patterns of variable current density were applied for electrosynthesis of formate (*n* = 3, respectively). Briefly, the first two concern the repeated adjustment of current density to low or no availability of solar and wind energy, whereas the third simulates the day–night cycle of solar energy.

**Figure 3:**
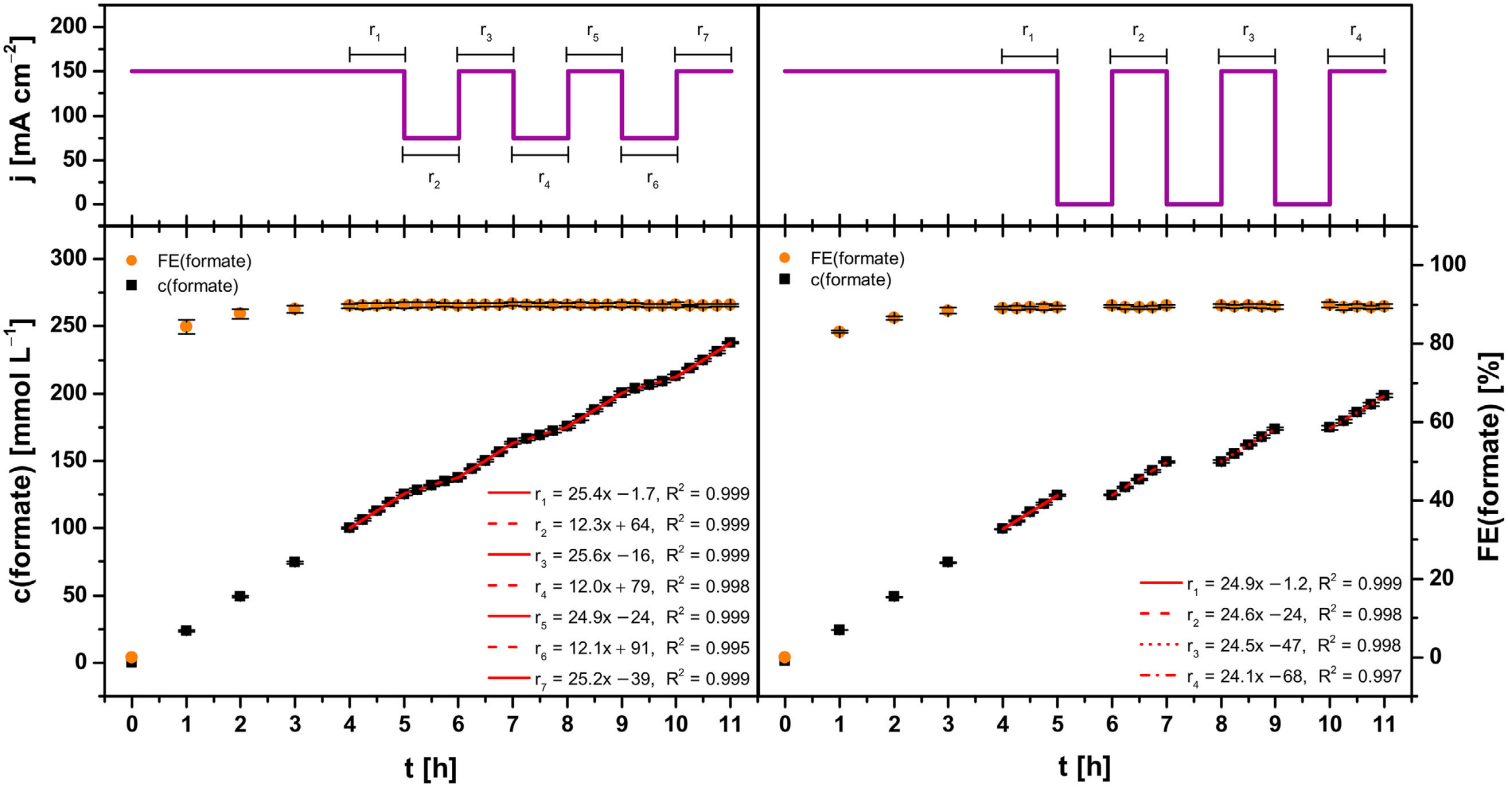
Concentration course and corresponding FE of formate during electrosynthesis at two variable current density patterns. The concentration course was fitted linearly in hourly intervals, respectively. Electrolysis parameters: runtime = 11 h, electrolyte = 0.2 mol L^−1^ KH_2_PO_4_/K_2_HPO_4_, initial *V* (catholyte, anolyte) = 500 mL each, membrane = Nafion 424, cathode (GDE, 5 cm^2^) = 87.5 wt% Bi/Bi_2_O_3_ (80:20), 12.5 wt% PTFE on Ni foam, reference electrode = RHE, anode = mixed Ir‐oxide on a Ti‐grid (Platinode EP, Type 177, Umicore).

**Figure** **3** depicts the first two courses of variable current density alongside total concentration/FE and synthesis rates of formate in the catholyte. In both cases, electrolysis was run at constant current density of 150 mA cm^−2^ (full load) for the first five hours to condition the GDE (as before during catalyst evaluation). Furthermore, the first synthesis rates of formate (r_1_, *t* = 4–5 h) were compared after conditioning to ensure initial performance of the GDEs were comparable to those of the previous evaluation (Figure 2).

For the first pattern, current density was hourly alternated three times between half load (75 mA cm^−2^) and full load (150 mA cm^−2^) after 5 h conditioning (Figure 3, left). Thereby, current density was adjusted using a relatively fast current ramp (60 s). Moreover, no other operational parameters (e.g., CO_2_ overpressure, electrolyte flow rate) were adjusted to examine the electrochemical and mechanical robustness of the GDE. The synthesis rate of formate started out from 25.43 ± 0.08 mmol L^−1^ h^−1^ (r_1_, *t* = 4–5 h) at full load and declined to 12.28 ± 0.12 mmol L^−1^ h^−1^ (r_2_, *t* = 5–6 h) in the first interval at half load. Halving the load approximately halved the synthesis rate. In the subsequent interval at full load again, the synthesis rate increased back up to 25.6 ± 0.3 mmol L^−1^ h^−1^ (r_3_, *t* = 6–7 h). Remarkably, comparable synthesis rates were also obtained with two further current load alternations.

Hence, alternating current density during electrosynthesis of formate between half (75 mA cm^−2^) and full load (150 mA cm^−2^) sustained the formate synthesis rate. This was accompanied by a relatively stable FE (≈90%) for formate throughout electrosynthesis after conditioning, which resulted in a total of 237.3 ± 1.5 mmol L^−1^ formate corresponding to an FE of 91.1 ± 0.5% for 11 h runtime (*n* = 3).

Regarding stability of the GDE, cathodic corrosion of Bi (electrocatalyst) and Ni (support material) were investigated as before using ICP‐OES (**Table** **3**). Analysis of the catholytes revealed the concentration of Bi was higher (≈55%) than for electrolyses at constant current density (Table 1). In terms of molar amounts (considering the increase in catholyte volume after electrolysis), 48% more Bi was dissolved from the GDE at alternating (full and half load) than at constant current density. Consequently, employing the first current density pattern for GDE operation led to more cathodic corrosion of Bi, although absolute values remained relatively low. In contrast, concentration and molar amount of Ni were similar to the results at constant current density. Overall, electrosynthesis of formate maintained consistently high FE and stable synthesis rates despite switching between full and half load repeatedly, while cathodic corrosion of Bi was slightly increased.

**Table 3:** Concentration of Bi^3+^ and Ni^2+^ in the catholyte after electrosynthesis of formate (*n* = 3) using Bi/Bi_2_O_3_ (80:20) based GDE at variable current load determined via ICP‐OES.

Current density [mA cm^−2^]	c(Bi^3+^) [μg L^−1^]	c(Ni^2+^) [μg L^−1^]
150 ⇌ 75	168 ± 37	27 ± 5
150 ⇌ 0	4416 ± 97	411 ± 97
150 → 50 → 150	134 ± 17	143 ± 63

For the second pattern, current density was hourly alternated three times between zero (0 mA cm^−2^) and full load (150 mA cm^−2^) after conditioning (Figure 3, right). As above, current density was adjusted with a steep current ramp (60 s) while no other operational parameters were adjusted throughout. The synthesis rate of formate started out from 24.94 ± 0.07 mmol L^−1^ h^−1^ (r_1_, *t* = 4–5 h) at full load. After the first interval at zero load, the synthesis rate increased back up to 24.6 ± 0.5 mmol L^−1^ h^−1^ (r_2_, *t* = 6–7 h) at full load again. After the second and third interval at zero load, the synthesis rate declined slightly from 24.5 ± 0.4 mmol L^−1^ h^−1^ (r_3_, *t* = 8–9 h) to 24.1 ± 0.7 mmol L^−1^ h^−1^ (r_4_, *t* = 10–11 h). Comparing the first and last synthesis rate, the decline was only about 3%. Hence, alternating current density between zero (0 mA cm^−2^) and full load (150 mA cm^−2^) impacted the synthesis rate of formate only slightly. Furthermore, FE for formate remained relatively stable throughout electrosynthesis (≈90%) after conditioning. This resulted in a total of 198.1 ± 1.7 mmol L^−1^ formate, which corresponded to 91.0 ± 0.8% FE for 11 h runtime (*n* = 3).

Regarding stability of the GDE, dissolution of Bi and Ni was greatly increased by alternating between zero and full load. The concentration of dissolved Bi in the catholyte (4416 ± 97 μg L^−1^) was about factor 27 higher than for the first pattern of variable load and factor 40 for electrolyses at constant current density (Table 1). Likewise, the concentration/amount of dissolved Ni was increased by about factor 20 in comparison to electrolyses without current alternation (Table 1). However, results of the alternation between half and full load indicate the intervals without any current are responsible for the increased dissolution of Bi and Ni instead of cathodic corrosion during full load. Consequently, degradation of the unpolarized GDE occurred most likely and the results cannot be attributed to cathodic corrosion alone.

Nevertheless, the second pattern of current (zero and full load) was suitable for formate electrosynthesis with high FE and relatively constant synthesis rates as well, but demonstrated stability and resilience of GDE could be an important issue in long‐term operation.

**Figure 4:**
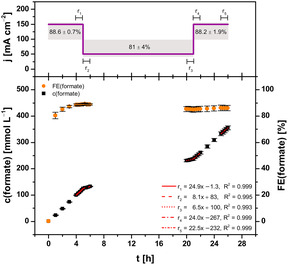
Concentration course and corresponding FE of formate during electrosynthesis at variable current density. The concentration course was fitted linearly in several intervals (r_1 = _4–5 h, r_2 = _5–6 h, r_3 = _20–21 h, r_4 = _21–22 h, r_5 = _25–26 h). Electrolysis parameters: runtime = 26 h, electrolyte = 0.2 mol L^−1^ KH_2_PO_4_/K_2_HPO_4_, initial *V* (catholyte, anolyte) = 500 mL each, membrane = Nafion 424, cathode (GDE, 5 cm^2^) = 87.5 wt% Bi/Bi_2_O_3_ (80:20), 12.5 wt% PTFE on Ni foam, reference electrode = RHE, anode = mixed Ir‐oxide on a Ti‐grid (Platinode EP, Type 177, Umicore).

The third and final pattern of electric current was based on the day–night cycle of electricity generation/demand. **Figure** **4** depicts the corresponding course of current density alongside total concentration, FE, and synthesis rates of formate in the catholyte. First, electrolyses were run at constant current density of 150 mA cm^−2^ (full load) for the first five hours as before to condition the GDE. Afterwards, current density was reduced to 50 mA cm^−2^ (night load) for 16 h throughout the night. Finally, current density was increased back up to 150 mA cm^−2^ (full load) until the end of electrolyses. As before, current density was adjusted using a steep current ramp (60 s) while no other operational parameters were adjusted.

The synthesis rate of formate started out from 24.93 ± 0.16 mmol L^−1^ h^−1^ (r_1_, *t* = 4–5 h) at full load. Thereby, total FE amounted to 88.6 ± 0.7% for the first interval of runtime at full load (*t* = 0–5 h). After switching to night load (*t* = 5–21 h), the synthesis rate of formate declined to 8.1 ± 0.3 mmol L^−1^ h^−1^ (r_2_, *t* = 5–6 h). This was about one third of the previous rate (r_1_), as expected at one third of current density. However, the synthesis rate declined throughout 16 h at night load about 20% to 6.5 ± 0.3 mmol L^−1^ h^−1^ (r_3_, *t* = 20–21 h). Consequently, total FE for the interval at night load was 81 ± 4%, almost 10% lower than for the previous interval at full load. Nonetheless, synthesis rate of formate increased back up to 23.97 ± 0.07 mmol L^−1^ h^−1^ (r_4_, *t* = 21–22 h) in the subsequent interval at full load. Taking an increase in catholyte volume throughout runtime into account, r_4_ was about the same as the initial r_1_. Hence, performance of the GDE at full load remained as good as before, even though it had been operated at lower efficiencies with night load. After a further five hours of operation at full load, the synthesis rate had declined about 6% to 22.5 ± 0.3 mmol L^−1^ h^−1^ (r_5_, *t* = 25–26 h). This was attributed to an increased influence of HER, as the buffer capacity was depleted and pH decreased from ≈6.67 to 5.59 ± 0.13 (*n* = 3) during this last interval (Section S3.2, Supporting Information). Since buffer capacity was a systematic limitation of the employed screening set‐up, its optimization for continuous electrosynthesis of formate could most likely prevent the decline of synthesis rates. Despite the slight decline of synthesis rate, the total FE for this interval was 88.2 ± 1.9%, which was comparable to the first interval at full load. It follows that operation at full load was not significantly impaired by shifting to night load intermediately for 16 h. However, a decrease in GDE performance was observed during night load, as described above. On the one hand, it could suggest there is a minimum current density for operation of GDE with stable synthesis rates of formate. On the other hand, no operational parameters were adjusted together with current density. Therefore, optimization of operating parameters during night load could possibly stabilize synthesis rates. Nonetheless, the whole electrosynthesis reached a total concentration of 355 ± 8 mmol L^−1^ for formate, which corresponded to a total FE of 85.9 ± 2.1% (*n* = 3).

Regarding stability of the GDE, dissolution of Bi for the day–night cycle was the lowest among all variable current patterns, but slightly higher than at constant current density. In contrast, the concentration of dissolved Ni was the second highest among the current patterns, and about factor 7 higher than at constant current density. Consequently, Ni appeared to be more affected by cathodic corrosion at 50 mA cm^−2^ than Bi, possibly due a probable change of the electrowetting state/three‐phase boundary position.

Overall, the third pattern of current (day–night cycle) was suitable for electrosynthesis of formate with high FE (≈88%) and relatively stable synthesis rates, particularly in intervals at full load. Although FE was slightly lower in the night interval and the synthesis rate declined, the GDE performance of the subsequent day interval was as high as before at full load. Furthermore, cathodic corrosion of Bi was only increased slightly by shifting between full and night load.

All in all, the different current patterns demonstrated that Bi/Bi_2_O_3_ (80:20) GDEs had an overall robust performance as they maintained high FE and relatively stable formate synthesis rates despite alternating current densities. The rapid adjustment of current density with a steep ramp (60 s) did not appear to affect their performance. However, alternating current density generally increased dissolution of Bi, but mostly if the electrolysis was turned on and completely off again. This suggests either an alternating mode of operation with a lower limit for current density, or an optimization of operational parameters at zero current to prevent loss of catalyst material in the long‐term. Additionally, a lower limit for current density was also indicated by the moderate loss of efficiency during night load (50 mA cm^−2^). Alternatively, the addition of ionomers to the GDE has recently been demonstrated to improve catalyst stability.^[^ ^47^ ^]^ To examine stability of the GDE at variable current density in the long‐term, time‐dependent studies should be carried out with a set‐up optimized for continuous electrosynthesis of formate employing real intermittent electricity patterns. Thereby, the flexible operation of GDE could be optimized to evaluate an implementation of decentralized utilization of intermittent electricity for CO_2_ reduction.

## 3 Conclusion

To avoid wasting renewable energy resources beyond demand and grid capacity, GDEs for flexible operation at intermittent electricity were successfully established. The GDE materials were based on Bi as inexpensive and relatively abundant electrocatalyst, to facilitate decentralized electrochemical reduction of CO_2_ to formic acid. This formic acid or formate can be stored and has versatile applications.

The catalyst composition of the GDE was optimized to Bi/Bi_2_O_3_ (80:20). It achieved the highest total FE/concentration of formate and the most stable synthesis rates of formate. Additionally, cathodic corrosion of Bi was minor. Without adaption of operational parameters, demonstrative current patterns based on intermittent electricity were applied. The GDE showed overall robust performance despite swift current adjustment. At repeated hourly alternation between full load (150 mA cm^−2^) and half load (75 mA cm^−2^), FE and synthesis rate of formate remained reliably high and stable. This was also the case for similar alternations between full load (150 mA cm^−2^) and zero load (0 mA cm^−2^). Consequently, electrolysis could be turned on and off flexibly as required by fluctuating renewable energy sources. Furthermore, electrolysis was also adapted to a day–night cycle of energy demand/generation. It was demonstrated for 26 h, whereby, full load (150 mA cm^−2^) was decreased to night load (50 mA cm^−2^) intermediately (16 h). Even though performance decreased moderately at night load, the GDE maintained high FE and stable synthesis rates of formate for intervals at full load. Noticeably, dissolution of Bi was only increased if the electrolysis was shut down completely (zero load). This needs to be addressed to prevent loss of catalyst in the long‐term. Overall, this study successfully demonstrates the flexible operation of GDE for the reduction of CO_2_ and thus hands out their future implementation to use intermittent electricity, support grid stability and a sustainable chemical industry.

## Conflict of Interest

The authors declare no conflict of interest.

## Supporting information

Supplementary Material

## Data Availability

The data that support the findings of this study are available in the supplementary material of this article.
