# Supplementary material for: CO2 Reduction to Formic Acid/Formate by Intermittent Electricity at Bismuth Gas Diffusion Electrodes
Source: ChemSusChem. 2025 Sep 30;18(23):e202501583. doi: 10.1002/cssc.202501583 (PMC12665880; doi:10.1002/cssc.202501583)
Supplement: Supplementary file 1 — Supplementary Material [file CSSC-18-e202501583-s001.pdf]

Supporting Information

CO_2 Reduction to Formic acid/Formate by Intermittent Electricity at Bismuth Gas Diffusion Electrodes

Ida Dinges, Siegfried R. Waldvogel, Markus Stöckl*

Outline

1 Experimental protocols and analytical methods ... S3
1.1 General Information ... S3
1.2 Fabrication of GDE ... S5
1.3 Electrochemical flow reactor and electrolysis set-up ... S7
1.4 Electrosynthesis of formate ... S7
1.5 Determination of density ... S8
1.6 Cross sections of GDE ... S10
1.7 Inductively coupled plasma optical emission spectroscopy (ICP-OES) ... S11
1.8 High-performance liquid chromatography (HPLC) ... S14
2 Calculations ... S15
2.1 Faradaic efficiency (FE) ... S15
2.2 Electric energy consumption (EEC) ... S15
3 Results ... S16
3.1 Electrosynthesis of formate at constant current density ... S16
3.2 Electrosynthesis of formate at variable current density ... S32
3.3 Pictures and cross sections of GDE before and after electrolysis ... S42

1 Experimental protocols and analytical methods

1.1 General Information

Selected chemical compounds and details on employed equipment are listed in this section (cf. Table S1, Table S2). All chemicals were used without further purification and all solutions were prepared using high purity H_2O (0.055 µS cm^{-1}, 25 °C, PURELAB Ultra).

Table S1: Selected chemical compounds (Purity grade, supplier, charge number).

Compound	Purity grade	Supplier	Charge
KH_2PO_4	>99% (p.a.)	Carl Roth, Karlsruhe / Germany	453340544
K_2HPO_4	>99% (p.a.)	Carl Roth, Karlsruhe / Germany	024350089
HCOOK	99% (p.a.)	Alfa Aesar, Haverhill / USA	10183323
HCOONa	≥99% (p.a.), ACS	Merck, Darmstadt / Germany	A0703243 608
H_2SO_4	75%, pure	Carl Roth, Karlsruhe / Germany	262322778
HNO_3	69%, ROTIPURAN Supra	Carl Roth, Karlsruhe / Germany	1121091
HCOOH	≥98%, for synthesis	Carl Roth, Karlsruhe / Germany	083329609

Table S2: Equipment / device, function and manufacturer.

Equipment / device	Function / use	Manufacturer
PURELAB Ultra	High purity H_2O	ELGA LabWater, High Wycombe / United Kingdom
A 10 basic	Knife mill	IKA, Staufen / Germany
LaboPress P200S	Heat press	Vogt Labormaschinen, Berlin / Germany
Micromar 40 ER	Thickness measurement	Mahr, Göttingen / Germany
Sartorius 1712004	Analytical scale (0.00000 g)	Sartorius Lab Instruments, Göttingen / Germany
Entris 3202I-1S	Scale (0.00 g)	Sartorius Lab Instruments, Göttingen / Germany
NGP804	Power supply	Rohde & Schwarz, Munich / Germany
CEBO-LC (CESYS C028152)	Analog data logging	CESYS, Herzogenaurach / Germany
GMH 3151	Pressure meter	GHM Messtechnik, Regenstauf / Germany
GMSD 2 BR - K31	Differential pressure sensor	GHM Messtechnik, Regenstauf / Germany
Transferpette® S	Pipetting, sampling (100-1000 µL, 500-5000 µL, 1000-10000 µL)	Brand, Wertheim / Germany
ECOLINE VC–MS/CA8-6	Peristaltic pump	ISMATEC Laboratoriums-technik, Wertheim / Germany

1.2 Fabrication of GDE

The gas diffusion electrodes (GDE) were fabricated by pressing catalyst mixture onto Ni foam as support material and current collector with a heat press. Each catalyst mixture (30.00 g) had the same electrocatalyst to binder ratio (87.5:12.5). As electrocatalysts, Bi (Purity 99.9, particle size <40 µm, Metallpulver24, Sankt Augustin / Germany) and / or Bi_2O_3 (Purity 99.9%, particle size approx. 80 nm, US Research Nanomaterials, Houston / USA) were used. Polytetrafluoroethylene (PTFE) powder (Dyneon™ PTFE TF 2072Z, 3M, Saint Paul / USA) served as hydrophobic binder. The different compositions are summarized in Table S3**.**

Table S3: Overview of the catalyst mixtures' composition of the fabricated GDEs.

GDE	*m* (Bi) / g	*m* (Bi_2O_3) / g	*m* (PTFE) / g
(A)	26.25	0	3.75
(B)	21.00	5.25	3.75
(C)	15.75	10.50	3.75
(D)	10.50	15.75	3.75
(E)	5.25	21.00	3.75
(F)	0	26.25	3.75

Each catalyst mixture was homogenized in a knife mill (A 10 basic). The mixing (30 s, 25000 rpm) was carried out twice and lead to a temperature increase of the mixture (T > 35 °C). After cooling to room temperature, the catalyst mixture was equally distributed onto Ni foam (d = 1.4 cm, 3.5 cm x 4.0 cm ≙ 14 cm^2, Ni-5763, density 420 - 450 g m^{-2}, Recemat BV, Dodewaard / Netherlands) with a sieve (stainless-steel wire mesh, mesh size = 500 µm, ISO 3310-1, Retsch / Verder Scientific, Haan / Germany) and a stencil (Cut-out 3.5 cm x 4.0 cm). The loading of the GDE with catalyst mixture was adjusted by differential weighting (Entris 3202I-1S) of the Ni foam. Afterwards, the GDE blank was placed in between two pieces of ordinary baking sheet in the heat press (LaboPress P200S) and compressed (plate temperature 120 °C, pressure 10 bar, 60 s). After compressing, excess material at the GDE edges was removed. The GDE's final catalyst loading *b* was determined by differential weighing (Sartorius 1712004) and its thickness *d* was measured at the center point/geometrical middle of the 14 cm^2 GDE area (Micromar 40 ER). A summary of all fabricated GDEs is provided in Table S4.

Table S4: Overview of electrocatalyst composition, catalyst mixture loading *b* and thickness *d* of the fabricated GDEs.

GDE	Composition	*b* (catalyst mixture) / mg cm^{-2}	*d* (GDE, center point) / µm
(A1)	Bi	78.21	461
(A2)	Bi	78.30	468
(A3)	Bi	78.52	486
(B1)	Bi / Bi_2O_3 (80:20)	80.19	475
(B2)	Bi / Bi_2O_3 (80:20)	79.66	498
(B3)	Bi / Bi_2O_3 (80:20)	78.10	493
(B4)	Bi / Bi_2O_3 (80:20)	80.29	496
(B5)	Bi / Bi_2O_3 (80:20)	78.24	491
(B6)	Bi / Bi_2O_3 (80:20)	79.54	498
(B7)	Bi / Bi_2O_3 (80:20)	80.48	487
(B8)	Bi / Bi_2O_3 (80:20)	78.52	491
(B9)	Bi / Bi_2O_3 (80:20)	79.92	486
(B10)	Bi / Bi_2O_3 (80:20)	80.81	494
(B11)	Bi / Bi_2O_3 (80:20)	78.34	494
(B12)	Bi / Bi_2O_3 (80:20)	78.99	496
(C1)	Bi / Bi_2O_3 (60:40)	77.12	500
(C2)	Bi / Bi_2O_3 (60:40)	80.24	520
(C3)	Bi / Bi_2O_3 (60:40)	81.48	511
(D1)	Bi / Bi_2O_3 (40:60)	77.67	516
(D2)	Bi / Bi_2O_3 (40:60)	76.55	514
(D3)	Bi / Bi_2O_3 (40:60)	75.95	516
(E1)	Bi / Bi_2O_3 (20:80)	73.47	515
(E2)	Bi / Bi_2O_3 (20:80)	71.09	508
(E3)	Bi / Bi_2O_3 (20:80)	72.57	521
(F1)	Bi_2O_3	74.33	521
(F2)	Bi_2O_3	74.77	544
(F3)	Bi_2O_3	74.68	558

1.3 Electrochemical flow reactor and electrolysis set-up

Detailed descriptions of the electrochemical flow reactor and the electrolysis set-up have already been provided in a previous publication (https://doi.org/10.1002/cssc.202301721). The same electrochemical flow reactor and electrolysis set-up have been employed for the electrosynthesis of formate herein. However, Nafion 424 (The Chemours Company, Wilmington, USA) was used as cation exchange membrane instead of Nafion 117. Nafion 424 is a reinforced and more stable membrane, which will be necessary for an upscaled reactor in the future. It has already been used here to enable a better comparison.

1.4 Electrosynthesis of formate

Electrosynthesis of formate using CO_2 was carried out with self-fabricated Bi / Bi_2O_3 based GDEs (cf. section 1.2) in a flow reactor (cf. section 1.3), whereby each GDE was only used once per experiment.

All electrosyntheses were run with a power supply unit (NGP804), which recorded terminal voltage (U), current (I) and power (P). The power supply unit was operated by analog input (NGP-K107) using *ProfiLab-Expert* (Version 4.0). Furthermore, the electrode potential of the GDE was referenced to a RHE (CEBO-LC). The electrosynthesis at constant current density started with a current ramp (60 s), in which the current density (j) reached 150 mA cm^{-2} (750 mA in total), which was maintained for 21 h runtime. The start-up procedure of the electrosynthesis at variable current density was identical, but the current density was adjusted during runtime with current ramps (60 s). The respective courses of current density are provided in section 3.2.

CO_2 (N4.5) was supplied to the GDE at a flow rate of 10 - 15 mL min^{-1} (Float-type flow meter, Wagner Mess- und Regeltechnik, Offenbach / Germany) and an initial overpressure in range of 50 - 200 mbar relative to ambient pressure. The pressure was recorded (every 2 s) during the running electrolysis (CEBO-LC).

Phosphate based buffer (0.2 mol L^{-1} KH_2PO_4 / K_2HPO_4, equimolar) served as electrolyte for all electrolyses. For each electrolysis, anolyte and catholyte had a starting volume of 500 mL (Volumetric flask, ISO 1042). Anolyte and catholyte were both circulated continuously at a flow rate of approx. 40 mL min^{-1} between flow reactor compartment and reservoir. Moreover, the catholyte reservoir was equipped with a pH electrode (EGA142, Xylem Analytics Germany Sales, Weilheim / Germany). The pH of the catholyte was recorded (every 2 s) during electrolysis (CEBO-LC). Catholyte samples were taken either hourly (1 mL, Transferpette® S) or every 15 min (200 µL, Transferpette® S) in certain intervals to monitor formate concentration and calculate the corresponding Faradaic efficiency (FE). After electrolysis, the catholyte volume was determined by its weight (Entris 3202I-1S) and density. The density was calculated

by taking samples (1 mL, n = 3, Transferpette® S) and weighing them (Sartorius 1712004). The GDE was rinsed with H_2O and dried at room temperature.

Details on the experimental parameters and results for all individual electrolyses generating formate are provided in section 3.1.

1.5 Determination of density

The densities of GDE were determined with the gas pycnometer BELPYCNO L (cf. Table S5), which was operated via *BELPycno-L* (Version 3.1.4).

Table S5: Volume / density measurement conditions performed on BELPYCNO L (Microtrac Retsch, Haan / Germany).

Conditions		Conditions	
Carrier gas	Helium	Restriction delta pressure	2.00000 bar
Temperature	20.00 °C	Equilibrium delta pressure	0.00020 bar
Reference volume	I	Equilibrium delta time	15 s
Flow cleaning time	0 s	Standard deviation (max.)	10%
Number of cleaning cycles	10	Nr. of good measurements	10
Sample cleaning time	5 s	Nr. of max. measurements	10
Atm. stabilization time	15 s	High precision mode	Disabled

The reference volume chamber I was calibrated with steel calibration sphere S using the instrument's standard protocol. All samples were measured (n = 3) in sample chamber S (20 cm^3) using glass beads (⌀ = 2.85 - 3.45 mm, charge 381176662, Carl Roth, Karlsruhe / Germany) as filler volume (approx. 50%). The sample weights (Sartorius 1712004) were used to calculate the densities from the measured volumes. GDE samples before electrolysis remained as fabricated (14 cm^2). To measure the GDE after electrolysis, the area exposed in the reactor during electrolysis was cut out (5 cm^2). As the sample volumes were relatively small compared to the sample chamber despite the filler, GDEs of the same composition were measured together (if available) in order to minimize relative errors. The results are summarized in Table S6.

Table S6: Volume and density results for Bi and / or Bi_2O_3 GDEs before and after electrolysis.

GDE composition	Electrolysis	Volume / cm^3	Density / g cm^{-3}
Bi	-	0.227 ± 0.003	7.37 ± 0.02
Bi	(A1), (A2)	0.190 ± 0.007	7.31 ± 0.09
Bi / Bi_2O_3 (80:20)	-	0.224 ± 0.004	7.46 ± 0.08
Bi / Bi_2O_3 (80:20)	(B1)	0.101 ± 0.002	7.2 ± 0.1
Bi / Bi_2O_3 (60:40)	-	0.223 ± 0.001	7.36 ± 0.04
Bi / Bi_2O_3 (60:40)	(C1)	0.108 ± 0.001	7.18 ± 0.04
Bi / Bi_2O_3 (40:60)	-	0.219 ± 0.004	7.47 ± 0.08
Bi / Bi_2O_3 (40:60)	(D1), (D2)	0.205 ± 0.001	7.11 ± 0.08
Bi / Bi_2O_3 (20:80)	-	0.168 ± 0.003	7.2 ± 0.2
Bi / Bi_2O_3 (20:80)	(E1), (E2)	0.173 ± 0.002	7.06 ± 0.09
Bi_2O_3	-	0.228 ± 0.001	7.13 ± 0.03
Bi_2O_3	(F1), (F2)	0.187 ± 0.001	6.97 ± 0.04

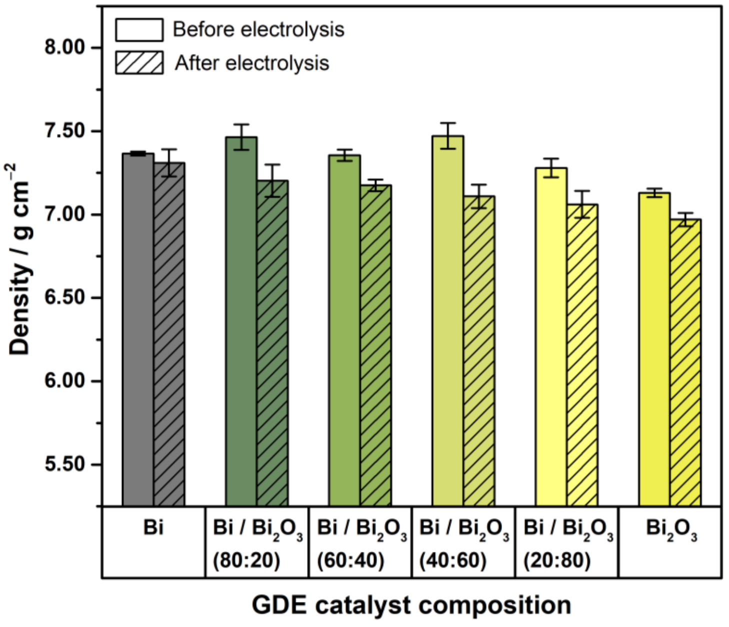

Figure S1: Density results of Bi / Bi_2O_3 GDEs before and after electrolysis (cf. Table S6).

1.6 Cross sections of GDE

Cross sections of GDEs were prepared before and after electrolysis. GDEs after electrolysis were dabbed dry with a paper towel without any prior rinsing. For preparation, each GDE was cut in half vertically with a scissor. Afterwards, one half was placed inside resin (transparent epoxy, Specifix-40, Struers, Ballerup / Denmark) with the cut edge facing upwards. All samples were vacuum impregnated using the CitoVac (Struers, Ballerup / Denmark). The cured, enclosed cross section was sanded with SiC paper (FEPA P320, P500, P1000, P2400 - Struers, Ballerup / Denmark) using water as coolant. Lastly, cross sections were polished with diamond paste (3 µm, 1 µm - Struers, Ballerup / Denmark). Sanding and polishing were both carried out with the Saphir 550 (ATM Qness, Mammelzen / Germany).

Pictures of the cross sections were taken with the bright-field microscope DM6000 M (Leica Microsystems, Wetzlar / Germany). Exemplary pictures are provided in section 3.3.

1.7 Inductively coupled plasma optical emission spectroscopy (ICP-OES)

ICP-OES measurements were performed on Agilent 5800 ICP-OES equipped with an SPS 4 Autosampler, a borosilicate double-pass spray chamber and a Seaspray concentric glass nebulizer (cf. Table S7). The system was operated via *ICP Expert* (Version 7.6.3.12735).

Table S7: ICP-OES measurement conditions performed on Agilent 5800 ICP-OES (Agilent Technologies, Santa Clara / USA).

Conditions		Conditions	
Replicate count	10	Viewing mode	Axial
Pump speed	12 rpm	Viewing height	-
Sample uptake time	25 s	Nebulizer flow	0.7 mL min^{-1}
Stabilization time	15 s	Plasma flow	12 mL min^{-1}
Read time	5 s	Aux Flow	1 mL min^{-1}
Rinse time	30 s	Oxygen content	0%
RF power	1.2 kW	IntelliQuant	Enabled

In between samples, autosampler and measurement system were rinsed with 2 wt% HNO_3 (prepared from 69 wt% HNO_3, Supra Quality, cf. Table S1).

All samples were measured without dilution except for acidification to 2 wt% HNO_3 (using 69 wt% HNO_3). In initial qualitative tests using an IntelliQuant screening, no elements of interest were detected in the anolyte samples. Consequently, only catholyte samples were examined further.

Standards to determine the concentrations of Bi^{3+} (λ = 306.771 nm) and Ni^{2+} (λ = 231.604 nm) were prepared from a stock solution (100 mg L^{-1}). The stock solution was prepared by combining the respective standards (10 mL each, cf. Table S8) in a volumetric flask (100 mL, ISO 1042) using 2 wt% HNO_3 for dilution. Afterwards, the solution was diluted further with 2 wt% HNO_3 to either 6 or 8 mg L^{-1} in a volumetric flask (100 mL, ISO 1042). This was followed by a dilution series by factor 2, respectively. Finally, both sets of standards (0.25, 0.5, 1, 2, 4, 8 mg L^{-1} and 0.1875, 0.375, 0.75, 1.5, 3, 6 mg L^{-1}) were diluted again by factor 2 with a matrix solution (0.4 mol L^{-1} KH_2PO_4 / K_2HPO_4, 0.5 mol L^{-1} HCOOH, 0.5 mol L^{-1} HCOOK in 2 wt% HNO_3). Thereby, a set of standards with a matrix based on the catholyte's composition was obtained. Furthermore, two additional standards (5 and 6 mg L^{-1}) were prepared from the initial stock solution (100 mg L^{-1}) by dilution to 10 and 12 mg L^{-1} with 2 wt% HNO_3, which were diluted again by factor 2 with matrix solution. In the following, the calibrations for each targeted analyte with either catholyte matrix or 2 wt% HNO_3 matrix are presented. The calibration with catholyte matrix was used to calculate the results for the catholyte samples.

Table S8: Single Element ICP-Standard-Solutions used for analyte quantification (Element, Concentration, Supplier, Lot. No.).

Element	Concentration	Supplier	Lot. No.
Bi	1000 mg L^{-1}	Carl Roth, Karlsruhe / Germany	794591
Ni	1000 mg L^{-1}	Carl Roth, Karlsruhe / Germany	974203

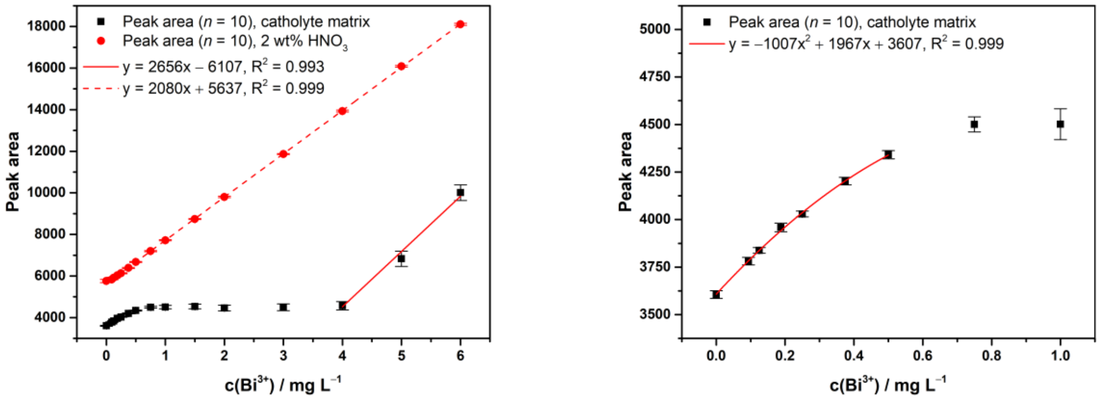

Figure S2: Calibration for Bi^{3+} (λ = 306.771 nm) via ICP-OES measurement with catholyte and 2% HNO_3 matrix**.** Plot of Bi^{3+} concentration (0, 0.09375, 0.125, 0.1875, 0.25, 0.375, 0.5, 1, 1.5, 2, 4, 5, 6 mg L^{-1}) against the peak area of the measured signal (n = 10) with a linear or quadratic fit.

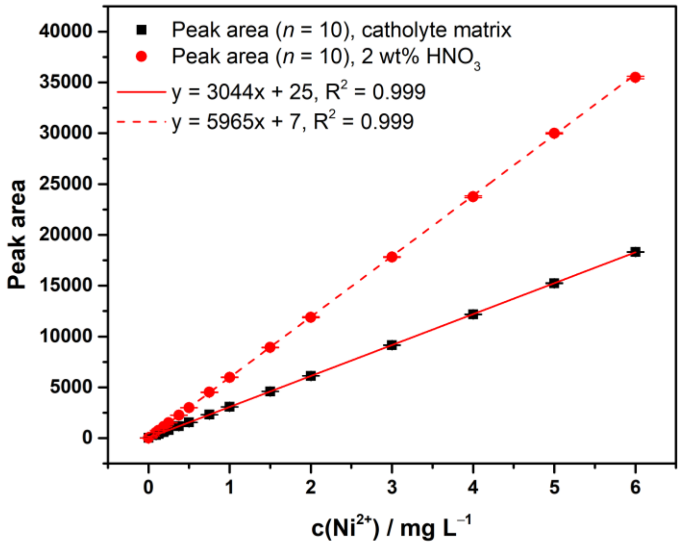

Figure S3: Calibration for Ni^{2+} (λ = 231.604 nm) via ICP-OES measurement with catholyte and 2% HNO_3 matrix**.** Plot of Ni^{2+} concentration (0, 0.09375, 0.125, 0.1875, 0.25, 0.375, 0.5, 1, 1.5, 2, 4, 5, 6 mg L^{-1}) against the peak area of the measured signal (n = 10) with a linear fit forced through zero, respectively.

Table S9: ICP-OES results for Bi^{3+} and Ni^{2+} concentrations in the catholyte samples.

Electrolysis	GDE catalyst composition	c(Bi^{3+}) / µg L^{-1}	c(Ni^{2+}) / µg L^{-1}
(P)[a]	-	0	0
(A1)	Bi	4099 ± 20	27.7 ± 1.6
(A2)	Bi	492 ± 35	28.7 ± 1.9
(A3)	Bi	192 ± 16	18.2 ± 2.0
(B1)	Bi / Bi_2O_3 (80:20)	4053 ± 10	22.4 ± 2.2
(B2)	Bi / Bi_2O_3 (80:20)	131 ± 11	21.9 ± 2.0
(B3)	Bi / Bi_2O_3 (80:20)	87 ± 6	17.2 ± 1.5
(B4)	Bi / Bi_2O_3 (80:20)	151 ± 7	23.9 ± 1.3
(B5)	Bi / Bi_2O_3 (80:20)	210 ± 9	24.9 ± 1.8
(B6)	Bi / Bi_2O_3 (80:20)	144 ± 10	31.9 ± 2.8
(B7)	Bi / Bi_2O_3 (80:20)	4489 ± 16	421 ± 4
(B8)	Bi / Bi_2O_3 (80:20)	4306 ± 13	308 ± 2
(B9)	Bi / Bi_2O_3 (80:20)	4452 ± 14	501 ± 4
(B10)	Bi / Bi_2O_3 (80:20)	117 ± 7	123.8 ± 2.4
(B11)	Bi / Bi_2O_3 (80:20)	137 ± 9	213.9 ± 3.3
(B12)	Bi / Bi_2O_3 (80:20)	150 ± 11	92.8 ± 0.9
(C1)	Bi / Bi_2O_3 (60:40)	110 ± 8	62.7 ± 2.1
(C2)	Bi / Bi_2O_3 (60:40)	95 ± 10	51.6 ± 1.8
(C3)	Bi / Bi_2O_3 (60:40)	99 ± 11	49.9 ± 1.0
(D1)	Bi / Bi_2O_3 (40:60)	126 ± 10	56.4 ± 2.6
(D2)	Bi / Bi_2O_3 (40:60)	122 ± 10	77.7 ± 2.2
(D3)	Bi / Bi_2O_3 (40:60)	102 ± 7	47.2 ± 1.5
(E1)	Bi / Bi_2O_3 (20:80)	79 ± 10	79.9 ± 2.7
(E2)	Bi / Bi_2O_3 (20:80)	83 ± 12	112.3 ± 2.3
(E3)	Bi / Bi_2O_3 (20:80)	95 ± 11	76.0 ± 1.5
(F1)	Bi_2O_3	97 ± 7	47.6 ± 1.6
(F2)	Bi_2O_3	113 ± 10	56.2 ± 2.1
(F3)	Bi_2O_3	124 ± 12	53.7 ± 1.1

[a] The phosphate buffer (0.2 mol L^{-1} KH_2PO_4 / K_2HPO_4) serving as supporting electrolyte for formate electrosynthesis was measured prior to electrolysis for comparison.

1.8 High-performance liquid chromatography (HPLC)

The quantification of formate was carried out via HPLC (cf. Table S10), the system was operated with the software *LabSolutions* (Version 5.93).

Table S10: HPLC measurement conditions for formate and PHB analysis performed on an HPLC unit (LC-20AD, SIL-20AC HT, CBM-20A, CTO-20AC, SPD-M20A - Shimadzu, Kyoto / Japan).

Conditions	Formate
Eluent	5 mmol L^{-1} H_2SO_4
Flow rate	0.6 mL min^{-1}
Pressure	30 ± 1
Column oven	35 °C
Column	Rezex ROA- Organic Acid (8%), 300 mm × 7.8 mm, Phenomenex, Torrance / USA
Injection volume	10 µL
Detector	Photodiode array (PDA)
Wavelength λ	194 nm
Retention time	14.9 min
Duration	25 min

Formate standards were prepared from a stock solution by a dilution series with the dilution factor 2. The stock solution was prepared with HCOONa (3.482 g, 51.2 mmol) in a volumetric flask (100 mL, ISO 1042).

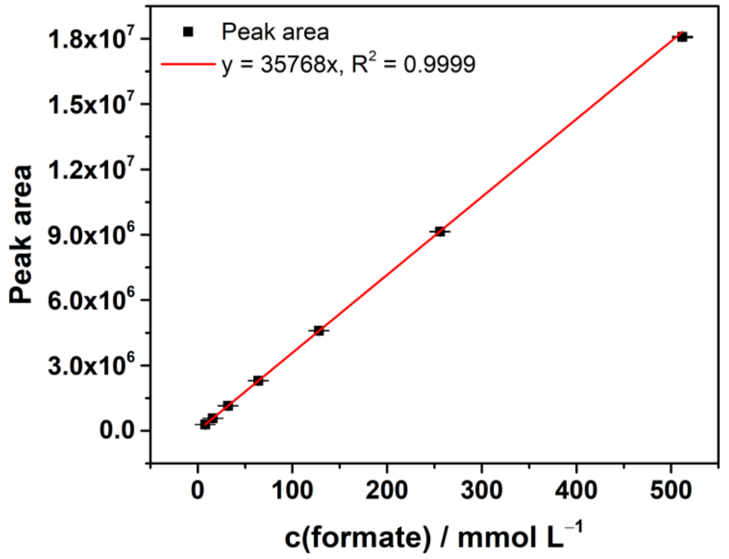

Figure S4: Calibration for formate quantification via HPLC measurement**.** Plot of formate concentration (8, 16, 32, 64, 128, 256, 512 mmol L^{-1}) against the peak area of the measured signal (n = 3) with a linear fit forced through zero.

2 Calculations

2.1 Faradaic efficiency (FE)

The FE for formate was calculated based on the determined amount of electrosynthesized formate using equation (1).

$$FE = \frac{F \cdot z \cdot n}{I \cdot t} \cdot 100\% \quad (1)$$

With FE = Faradaic efficiency / %, F = Faraday constant / A s mol^{-1}, z = Number of transferred electrons (z = 2), n = Amount of synthesised formate / mol, I = Current / A, t = Electrolysis runtime / s.

The results for the different catholytes were averaged and their standard deviation was provided as uncertainty.

2.2 Electric energy consumption (EEC)

The EEC for formate was calculated based on the determined amount of electrosynthesized formate using equation (2).

$$EEC = \frac{F \cdot z \cdot U}{FE \cdot M} \cdot (2.778 \cdot 10^{-7}) \quad (2)$$

With EEC = Electric energy consumption / kWh kg^{-1}, F = Faraday constant / A s mol^{-1}, z = Number of transferred electrons per molecule (z = 2), U = Averaged cell voltage / V, FE = Faradaic efficiency / %, M = Molar mass of formate / kg mol^{-1}.

The results for the different catholytes were averaged and their standard deviation was provided as uncertainty.

3 Results

This chapter contains detailed data for all electrolyses carried out at constant current density (cf. section 3.1) and variable current density (cf. section 3.2). Furthermore, pictures of the self-fabricated GDEs and their cross sections before and after electrolysis are provided (cf. section 3.3).

3.1 Electrosynthesis of formate at constant current density

A comparative overview of the results is presented in Table S11, operational electrolysis parameters have been summarized in Table S12, further electrolysis results have been collected in Table S13. Moreover, detailed courses of each individual electrolysis are provided.

Discussion of cell voltage and potential of GDE

Electrolyses were run with an average cell voltage of 6.46 ± 0.12 V for all different compositions of catalyst (n = 18). The generally relatively high cell voltage was mainly caused by ohmic losses in the anode chamber, as a non-zero gap anode was used for the oxygen evolution reaction (OER) as counter reaction of CO_2 reduction to formate. Accordingly, the average potential of GDEs was only −1.57 ± 0.14 V vs. RHE for all different compositions of catalyst (n = 18). As expected, the average potential of Bi GDE (−1.7 ± 0.1 V) was higher than that of Bi_2O_3 GDE (−1.52 ± 0.13 V). Despite that, there was no clear trend in between. Starting from Bi GDE, the average potential of GDE decreased with addition of Bi_2O_3 until Bi/Bi_2O_3 (60:40) GDE (−1.5 ± 0.1 V), as expected. However, the following Bi/Bi_2O_3 (40:60) GDE (−1.7 ± 0.3 V) and Bi/Bi_2O_3 (20:80) GDE (−1.37 ± 0.14 V) deviated from the preliminary trend, whereby Bi/Bi_2O_3 (20:80) had the overall lowest average potential. Consequently, stepwise addition of Bi_2O_3 as a reductive binder did not lead to the presumed stepwise lowering of GDE potential / increase in conductivity of the GDE. Besides, the average cell voltage of the different compositions showed the same relative correlations as the average potential of GDE, except for Bi/Bi_2O_3 (40:60). Thereby, Bi GDE required the highest average cell voltage (6.63 ± 0.06 V) whereas Bi/Bi_2O_3 (20:80) GDE required the lowest (6.3 ± 0.2 V).

Table S11: Comparative overview of results (expansion of Table 2 in the main text) for electrosynthesis of formate at constant current density (150 mA cm^{-2}, 21 h) with GDEs of variable catalyst composition (*n* = 3, respectively).

Catalyst composition of GDE	GDE catalyst[a)] cost [€ m^{-2}]	GDE material[b)] cost [€ m^{-2}]	c(formate) [mmol L^{-1}]	FE [%]	U[c)] [V]	E(GDE)[c)] [V]	EEC [kWh kg^{-1}]	c(formate) rate r_1[d)] [mmol L^{-1} h^{-1}]	c(formate) rate r_2[e)] [mmol L^{-1} h^{-1}]	pH[f)]
Bi	34.3 ± 0.1	376.9 ± 0.1	488 ± 3	87.5 ± 1.1	6.63 ± 0.06	−1.7 ± 0.1	8.84 ± 0.05	24.71 ± 0.09	17.45 ± 0.14	4.20 ± 0.01
Bi / Bi_2O_3 (80:20)	55.5 ± 0.1	398.3 ± 0.1	501 ± 5	90.3 ± 1.2	6.48 ± 0.06	−1.6 ± 0.1	8.37 ± 0.13	25.62 ± 0.12	20.16 ± 0.14	4.13 ± 0.03
Bi / Bi_2O_3 (60:40)	76.6 ± 2.2	419 ± 3	485 ± 15	87 ± 3	6.47 ± 0.01	−1.5 ± 0.1	8.6 ± 0.3	25.04 ± 0.11	16.8 ± 0.5	4.19 ± 0.03
Bi / Bi_2O_3 (40:60)	94.0 ± 1.1	436.6 ± 1.2	483 ± 5	87.0 ± 0.9	6.44 ± 0.08	−1.7 ± 0.3	8.63 ± 0.15	25.16 ± 0.13	15.4 ± 0.3	4.22 ± 0.01
Bi / Bi_2O_3 (20:80)	107.7 ± 1.8	450.0 ± 1.9	462 ± 18	83 ± 3	6.3 ± 0.2	−1.37 ± 0.14	8.79 ± 0.34	25.41 ± 0.17	12.9 ± 0.3	4.31 ± 0.08
Bi_2O_3	130.5 ± 0.5	473.0 ± 0.5	488 ± 6	88.0 ± 1.2	6.4 ± 0.3	−1.52 ± 0.13	8.55 ± 1.5	25.3 ± 0.2	19.0 ± 0.3	4.19 ± 0.02

[a)] Bi and/or Bi_2O_3; [b)] Including electrocatalyst (Bi and/or Bi_2O_3), PTFE and Ni foam; [c)] Average of 21 h runtime, without compensation for *iR* losses; [d)] *t* = 4 - 5 h; [e)] *t* = 20 - 21 h; [f)] After electrolysis (*t* = 21 h).

Table S12: Overview of operational electrolysis parameters for electrosynthesis of formate using 0.2 mol L^{-1} KH_2PO_4 / K_2HPO_4 as electrolyte. All values are given as average with standard deviation for the 21 h electrolysis duration, excluding the absolute electric energy consumption (EEC).

Electrolysis	OCP vs. RHE / V[a]	E(GDE) vs. RHE / V	U / V	EEC / Wh	$p(CO_2)$ / mbar
(A1)	0.950 ± 0.002	−1.63 ± 0.13	6.6 ± 0.7	103.6	98 ± 5
(A2)	0.966 ± 0.001	−1.82 ± 0.11	6.7 ± 0.7	105.1	252 ± 10
(A3)	0.901 ± 0.001	−1.74 ± 0.11	6.7 ± 0.6	105.1	78 ± 5
(B1)	0.817 ± 0.002	−1.47 ± 0.16	6.5 ± 0.8	102.3	116 ± 19
(B2)	0.965 ± 0.001	−1.67 ± 0.14	6.4 ± 0.7	101.3	174 ± 22
(B3)	0.991 ± 0.001	−1.56 ± 0.14	6.5 ± 0.7	102.9	147 ± 10
(C1)	0.543 ± 0.001	−1.46 ± 0.17	6.5 ± 0.7	102.0	141 ± 42
(C2)	0.586 ± 0.002	−1.63 ± 0.15	6.5 ± 0.6	101.9	179 ± 17
(C3)	0.510 ± 0.003	−1.53 ± 0.16	6.5 ± 0.6	101.8	114 ± 7
(D1)	0.503 ± 0.001	−2.0 ± 0.2	6.5 ± 0.7	102.6	230 ± 18
(D2)	0.506 ± 0.001	−1.67 ± 0.10	6.4 ± 0.6	101.7	208 ± 21
(D3)	0.836 ± 0.001	−1.52 ± 0.11	6.4 ± 0.6	100.4	147 ± 20
(E1)	0.296 ± 0.003	−1.21 ± 0.14	6.1 ± 0.6	96.7	191 ± 27
(E2)	0.512 ± 0.001	−1.43 ± 0.15	6.4 ± 0.6	101.7	148 ± 17
(E3)	0.41 ± 0.02	−1.47 ± 0.16	6.2 ± 0.6	98.4	218 ± 19
(F1)	0.262 ± 0.001	−1.51 ± 0.13	6.3 ± 0.7	99.9	260 ± 18
(F2)	1.13 ± 0.04	−1.40 ± 0.11	6.3 ± 0.7	99.7	182 ± 11
(F3)	1.15 ± 0.04	−1.65 ± 0.14	6.7 ± 0.8	105.6	227 ± 12

[a] Average with standard deviation measured for 5 min prior to electrolysis.

Table S13: Overview of volume, formate concentration, formate FE and pH determined *n* = 3 after 21 h electrolysis in the catholyte of formate electrosynthesis with 0.2 mol L^{-1} KH_2PO_4 / K_2HPO_4 as starting electrolyte.

Electrolysis	V / mL	c(formate) / mmol L^{-1}	FE(formate) / %	pH
(A1)	522.9 ± 0.2	485.0 ± 0.3	86.32 ± 0.04	4.20 ± 0.05
(A2)	528.6 ± 0.4	488.0 ± 0.4	86.79 ± 0.04	4.21 ± 0.05
(A3)	529.9 ± 0.3	490.2 ± 0.2	88.41 ± 0.03	4.19 ± 0.05
(B1)	527.1 ± 0.4	496.2 ± 0.3	89.01 ± 0.06	4.10 ± 0.05
(B2)	530.4 ± 0.6	503.4 ± 0.2	90.87 ± 0.08	4.14 ± 0.05
(B3)	530.2 ± 0.3	504.1 ± 0.2	90.97 ± 0.07	4.14 ± 0.05
(C1)	527.2 ± 0.1	469.8 ± 0.2	84.29 ± 0.03	4.21 ± 0.05
(C2)	531.4 ± 0.4	484.8 ± 0.1	87.67 ± 0.07	4.20 ± 0.05
(C3)	530.0 ± 0.2	499.5 ± 0.2	90.11 ± 0.05	4.16 ± 0.05
(D1)	529.3 ± 0.3	484.1 ± 0.2	87.21 ± 0.06	4.21 ± 0.05
(D2)	528.9 ± 0.4	478.3 ± 0.3	86.09 ± 0.05	4.23 ± 0.05
(D3)	529.5 ± 0.3	486.4 ± 0.1	87.65 ± 0.06	4.12 ± 0.05
(E1)	530.1 ± 0.6	446.3 ± 0.2	80.5 ± 0.1	4.38 ± 0.05
(E2)	531.0 ± 0.3	458.6 ± 0.2	82.86 ± 0.08	4.32 ± 0.05
(E3)	528.5 ± 0.5	480.2 ± 0.3	86.38 ± 0.06	4.23 ± 0.05
(F1)	529.6 ± 0.5	491.2 ± 0.5	88.53 ± 0.05	4.19 ± 0.05
(F2)	529.2 ± 0.5	481.0 ± 0.1	86.64 ± 0.07	4.21 ± 0.05
(F3)	531.2 ± 0.4	490.8 ± 0.2	88.73 ± 0.04	4.18 ± 0.05

3.1.1 Bi GDEs

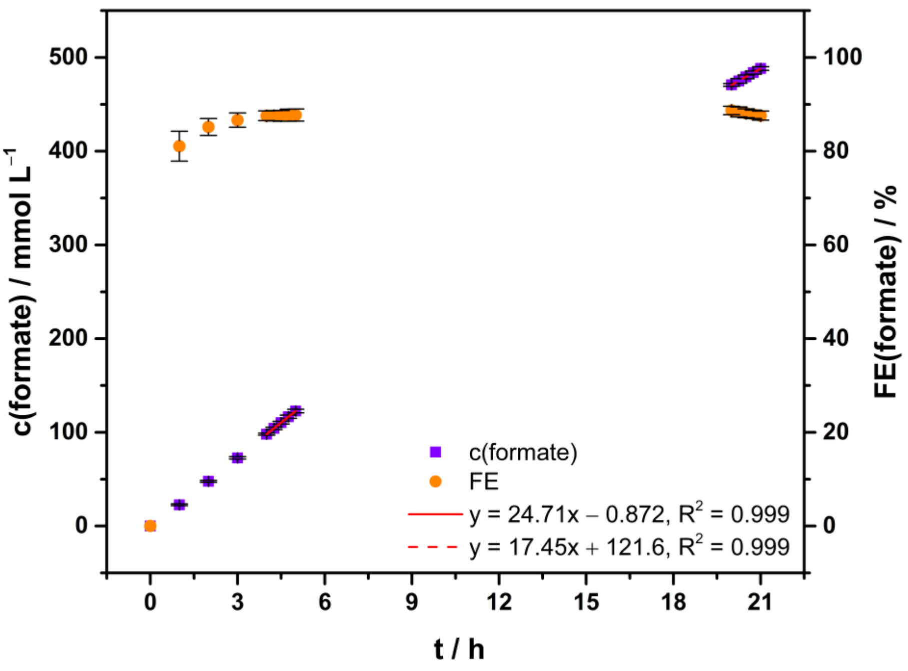

Figure S5: Data for electrolysis (A1, A2, A3), experimental details are provided in section 1.4 and results in Table S11, Table S12 and Table S13.

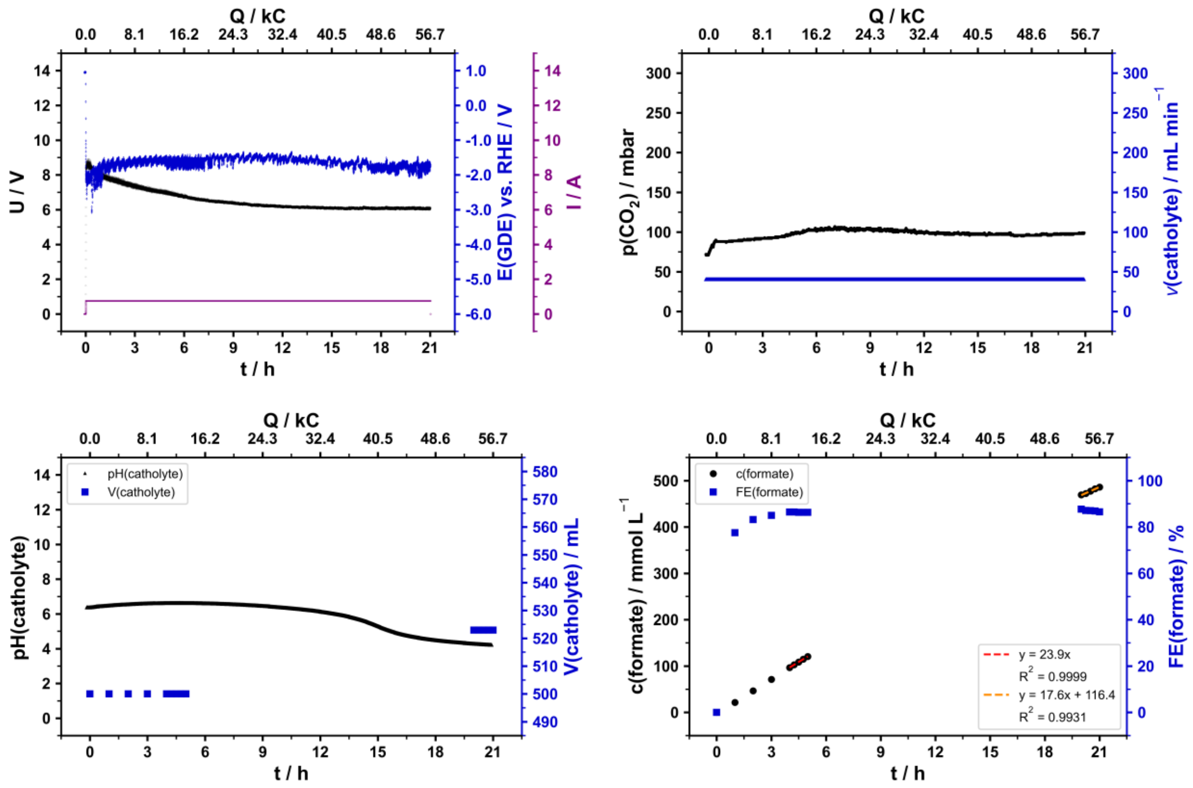

Figure S6: Data for electrolysis (A1), experimental details are provided in section 1.4 and results in Table S12 and Table S13.

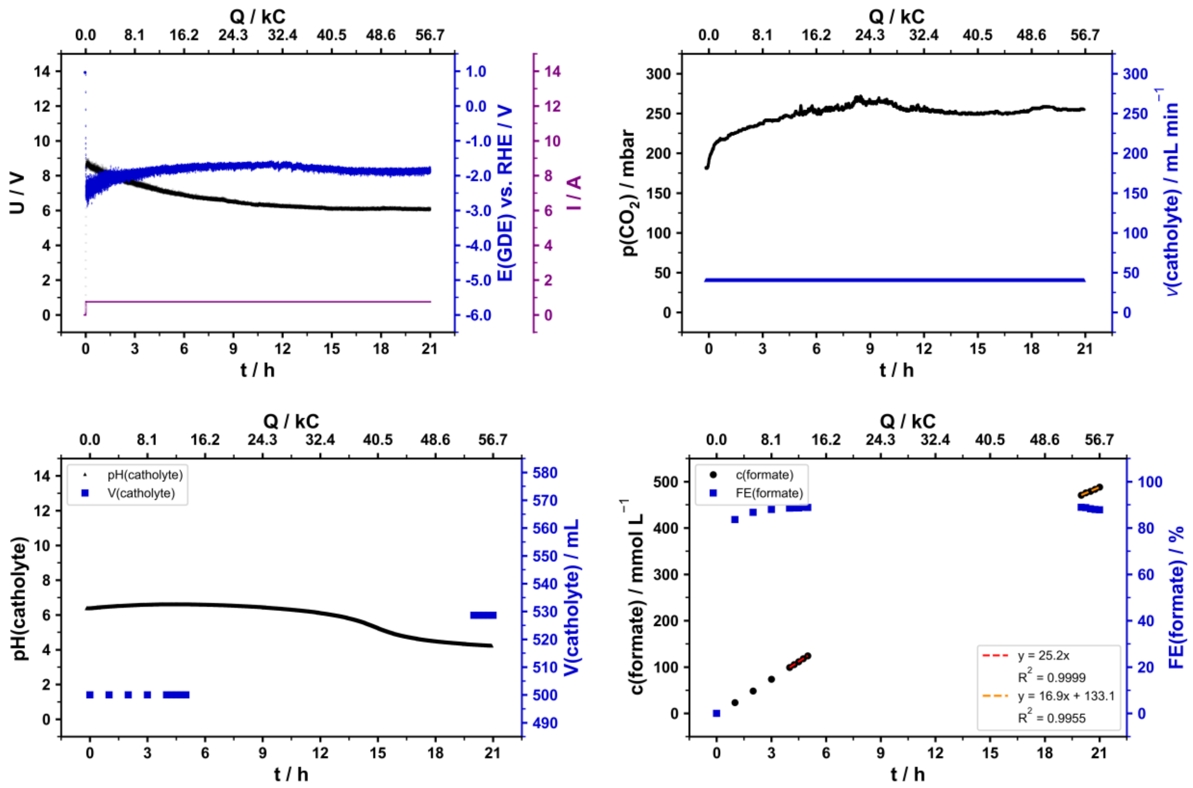

Figure S7: Data for electrolysis (A2), experimental details are provided in section 1.4 and results in Table S12 and Table S13.

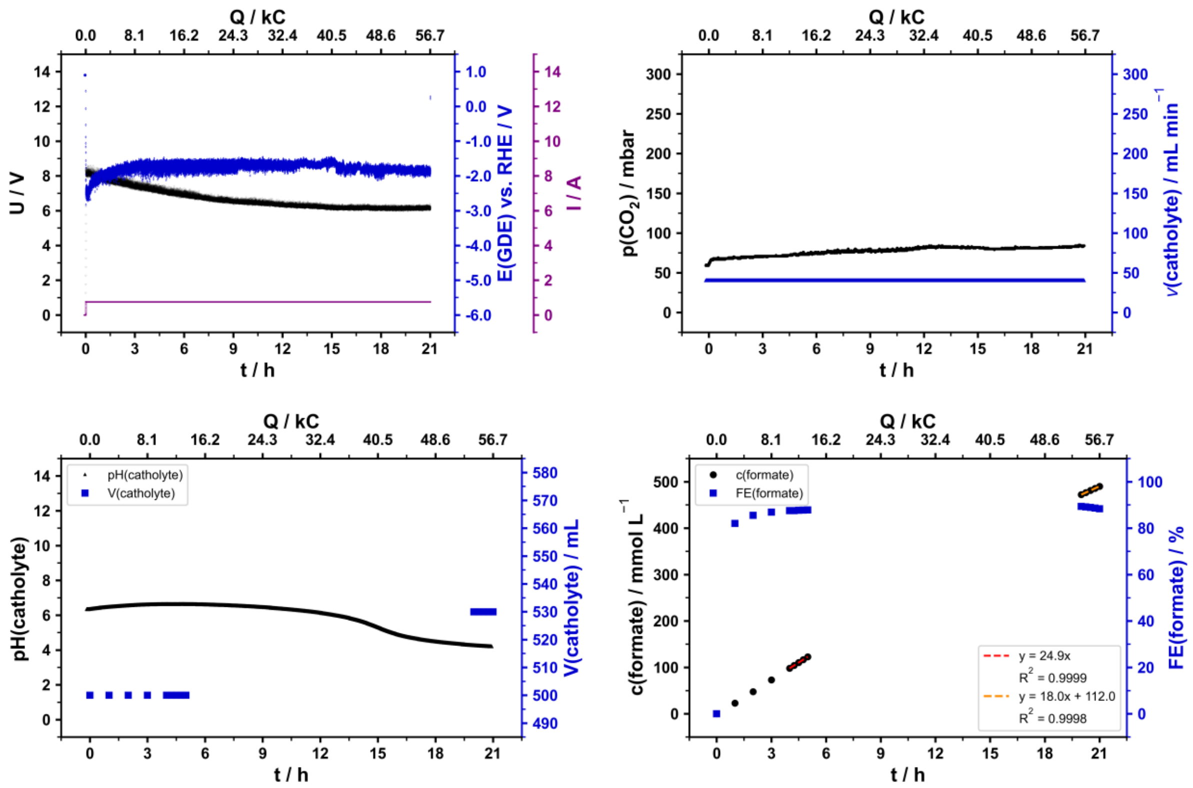

Figure S8: Data for electrolysis (A3), experimental details are provided in section 1.4 and results in Table S12 and Table S13.

3.1.2 Bi / Bi_2O_3 (80:20) GDEs

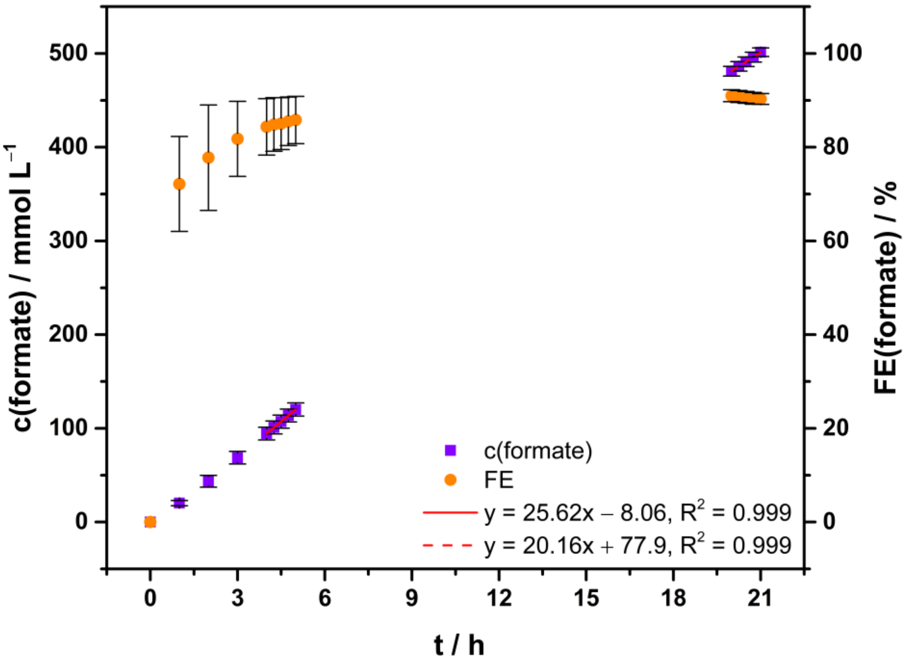

Figure S9: Data for electrolysis (B1, B2, B3), experimental details are provided in section 1.4 and results in Table S11, Table S12 and Table S13.

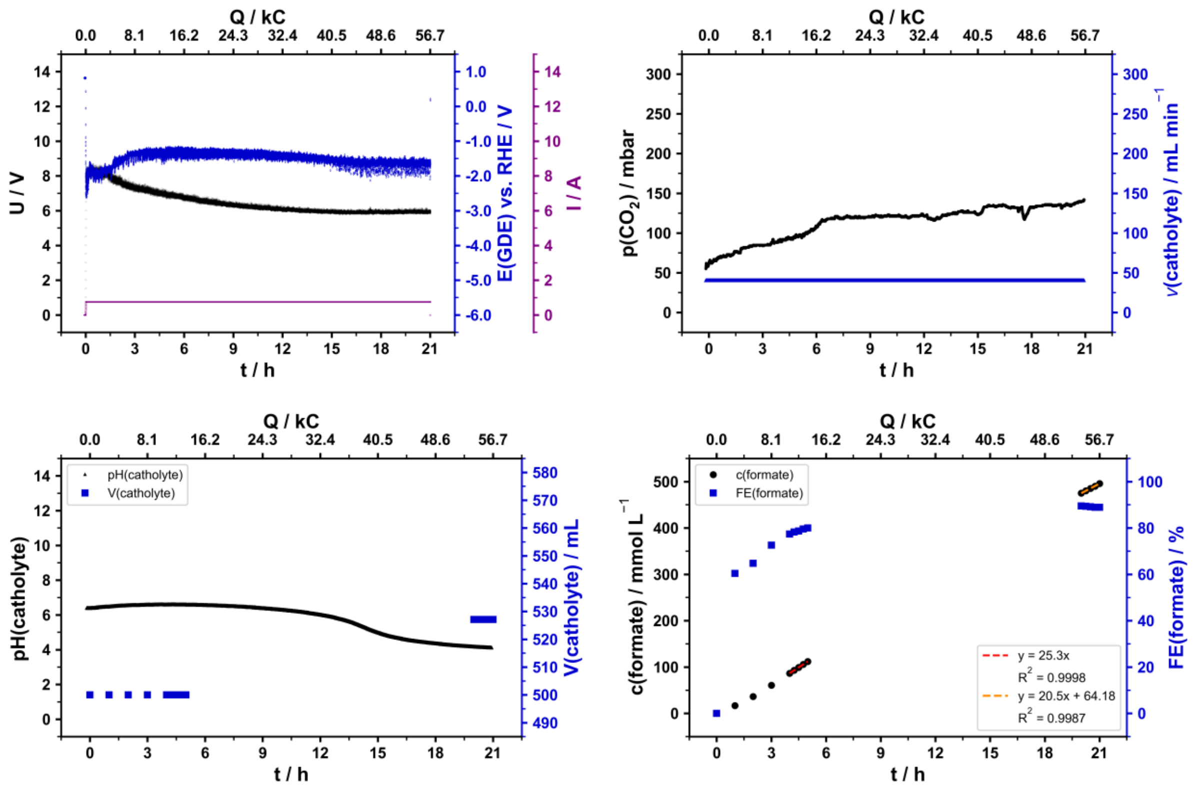

Figure S10: Data for electrolysis (B1), experimental details are provided in section 1.4 and results in Table S12 and Table S13.

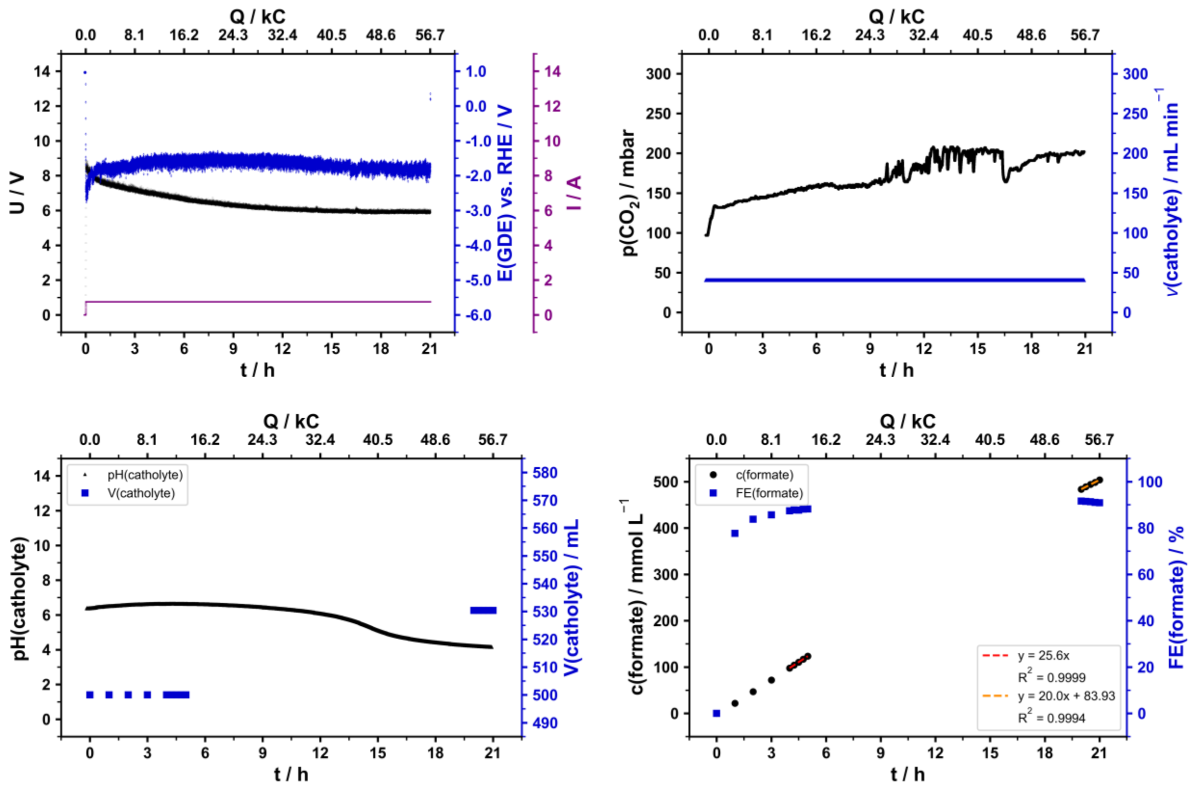

Figure S11: Data for electrolysis (B2), experimental details are provided in section 1.4 and results in Table S12 and Table S13.

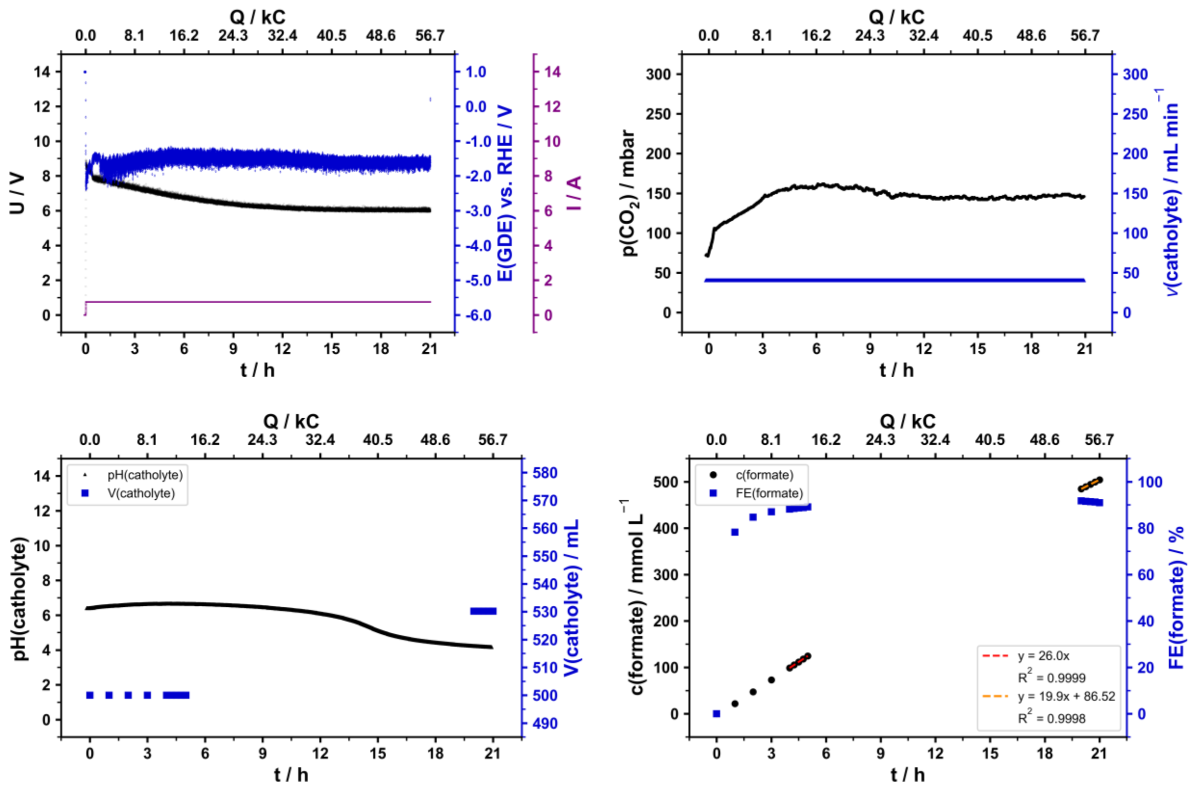

Figure S12: Data for electrolysis (B3), experimental details are provided in section 1.4 and results in Table S12 and Table S13.

3.1.3 Bi / Bi_2O_3 (60:40) GDEs

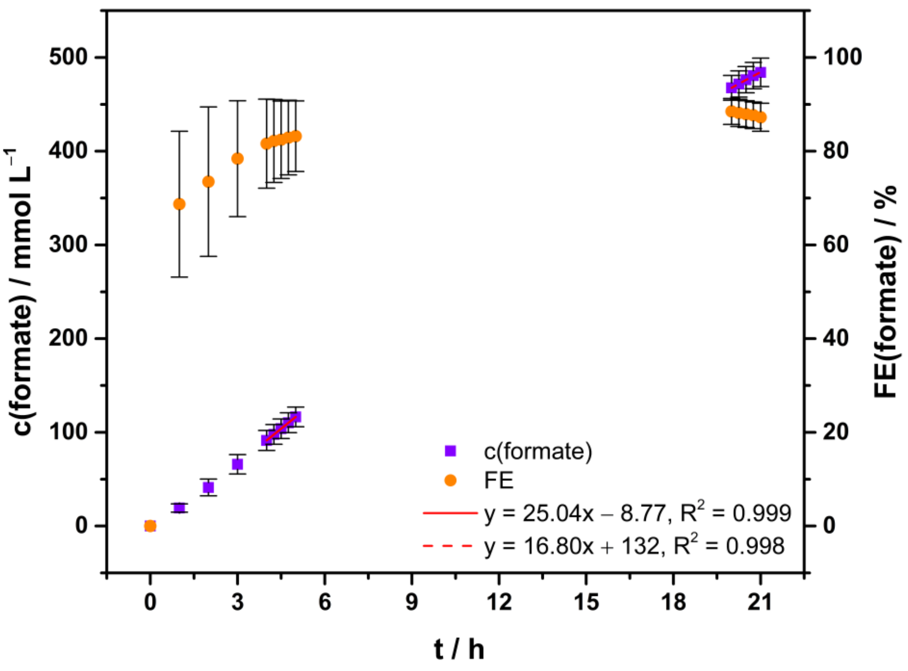

Figure S13: Data for electrolysis (C1, C2, C3), experimental details are provided in section 1.4 and results in Table S11, Table S12 and Table S13.

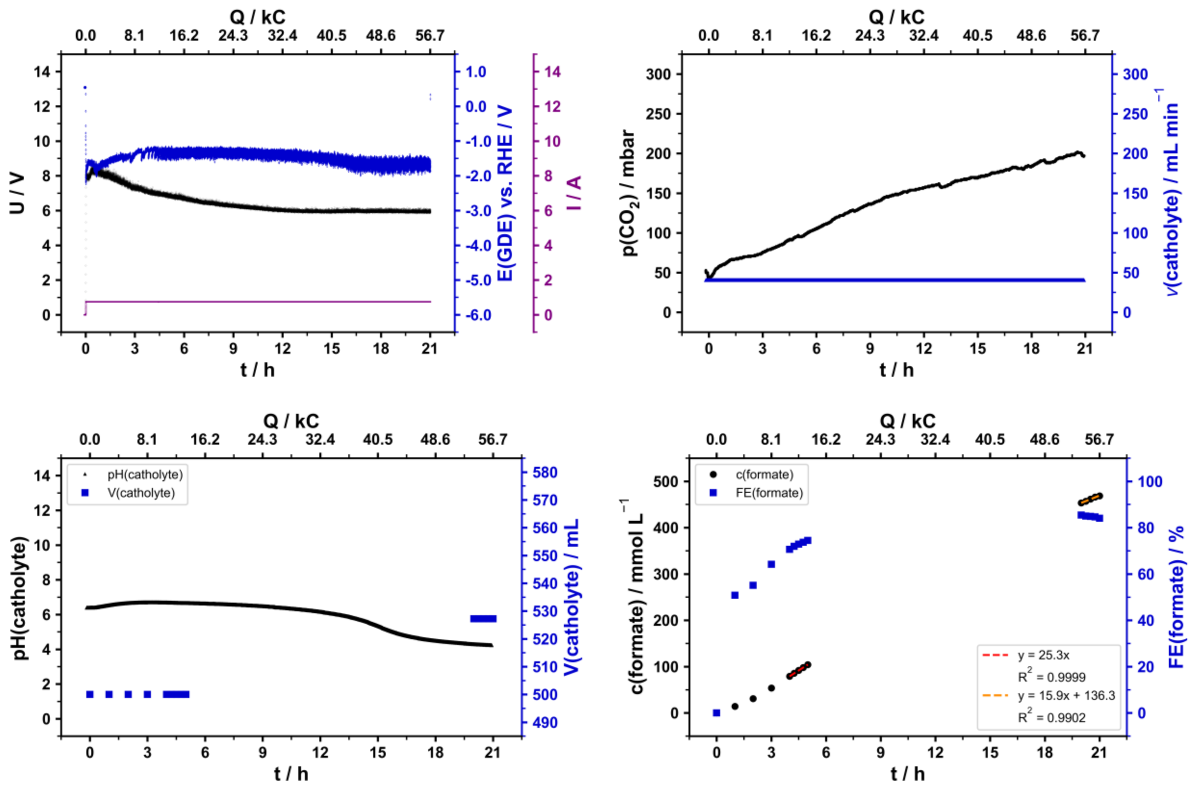

Figure S14: Data for electrolysis (C1), experimental details are provided in section 1.4 and results in Table S12 and Table S13.

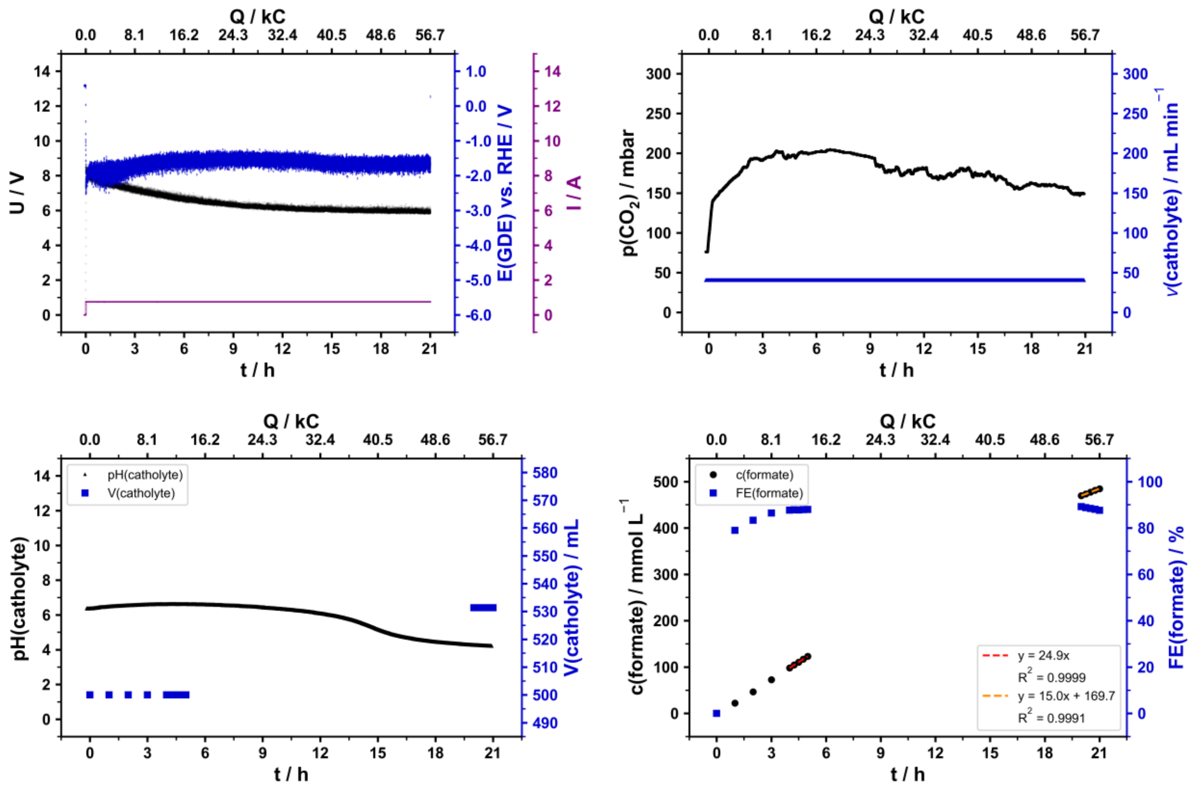

Figure S15: Data for electrolysis (C2), experimental details are provided in section 1.4 and results in Table S12 and Table S13.

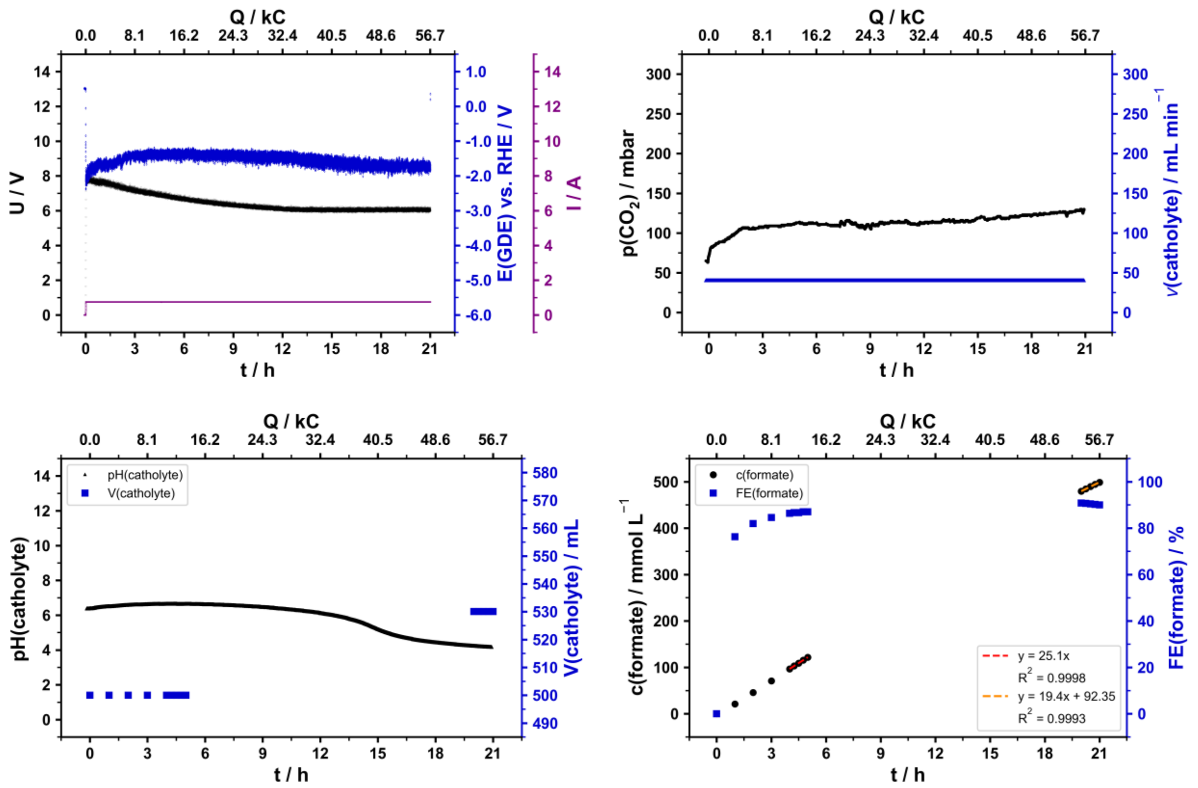

Figure S16: Data for electrolysis (C3), experimental details are provided in section 1.4 and results in Table S12 and Table S13.

3.1.4 Bi / Bi_2O_3 (40:60) GDEs

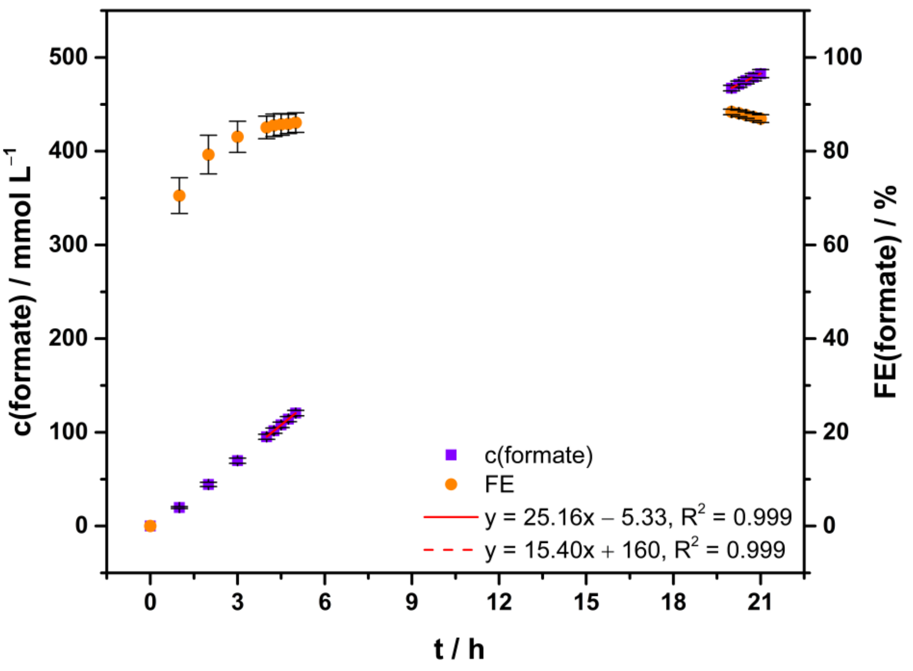

Figure S17: Data for electrolysis (D1, D2, D3), experimental details are provided in section 1.4 and results in Table S11, Table S12 and Table S13.

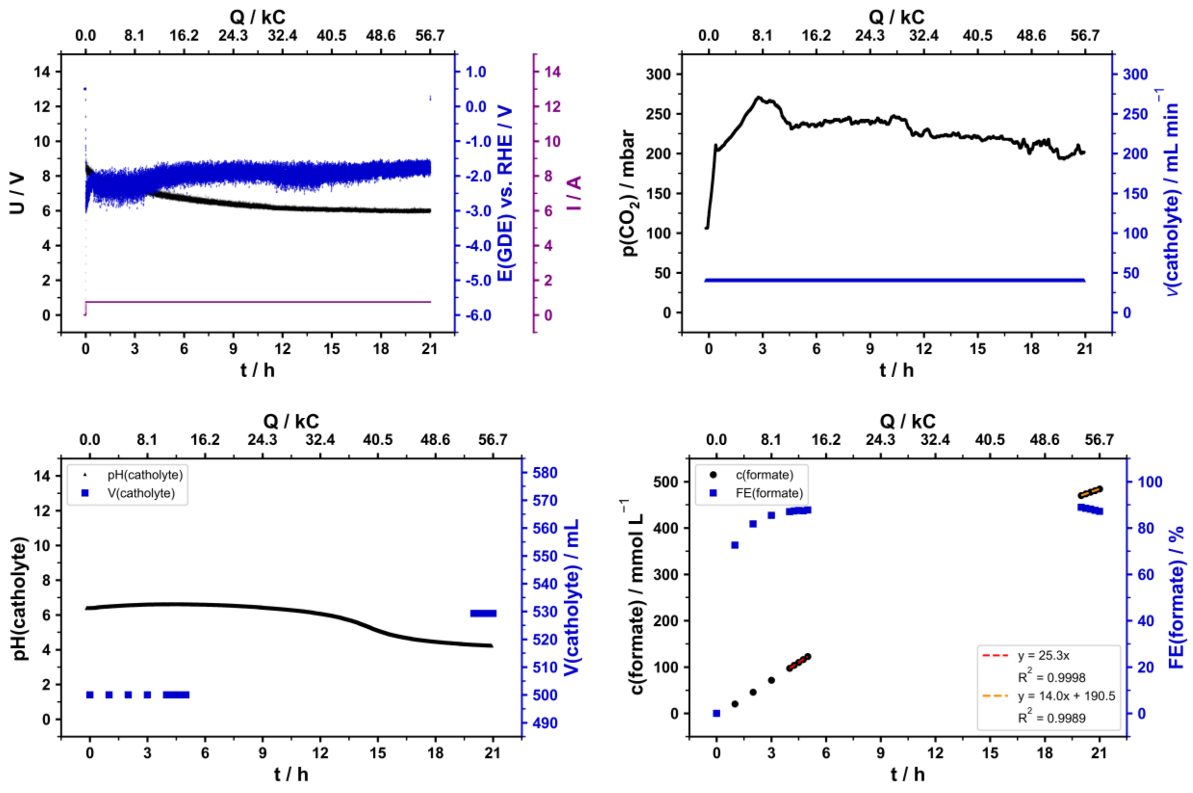

Figure S18: Data for electrolysis (D1), experimental details are provided in section 1.4 and results in Table S12 and Table S13.

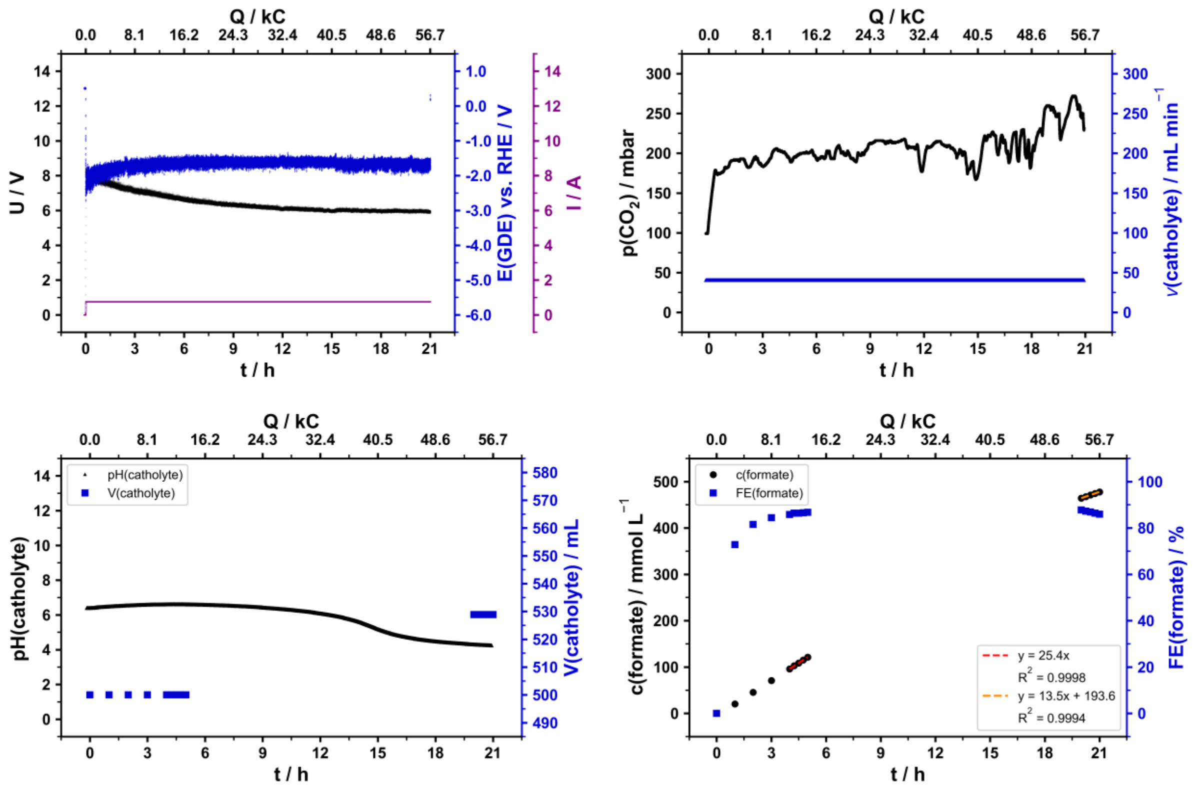

Figure S19: Data for electrolysis (D2), experimental details are provided in section 1.4 and results in Table S12 and Table S13.

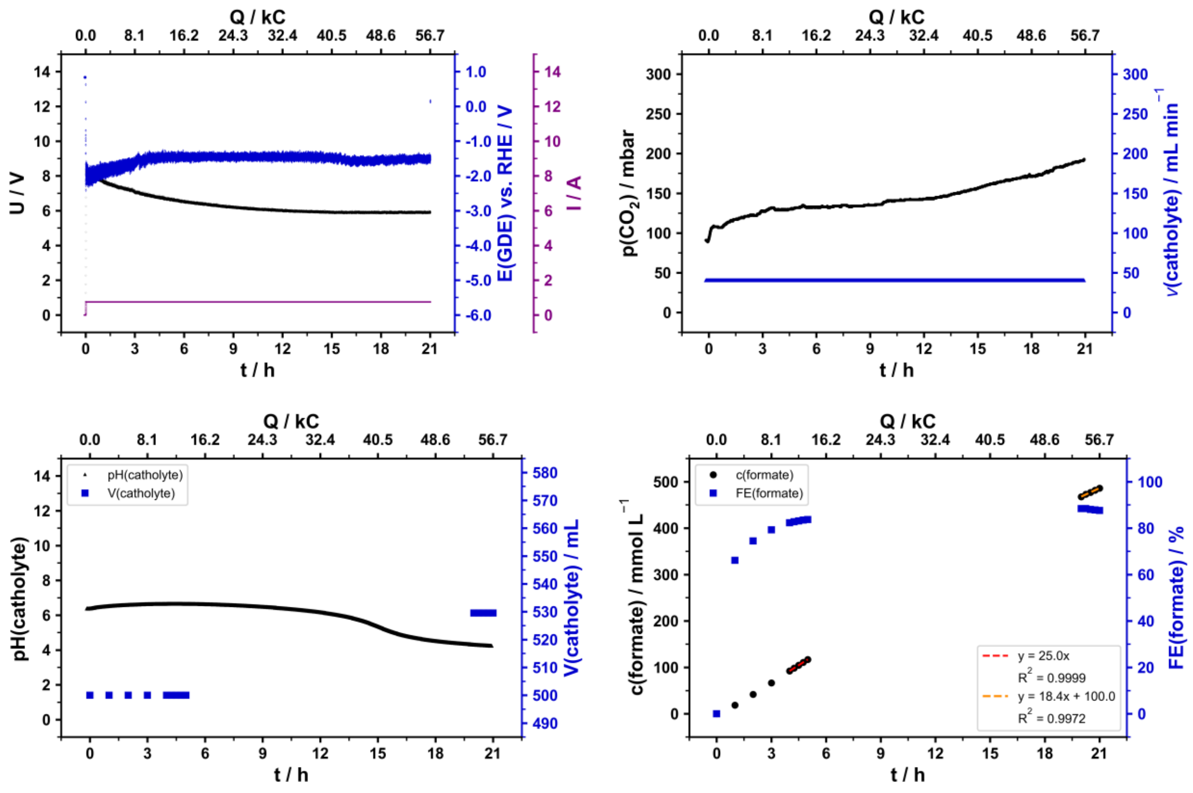

Figure S20: Data for electrolysis (D3), experimental details are provided in section 1.4 and results in Table S12 and Table S13.

3.1.5 Bi / Bi_2O_3 (20:80) GDEs

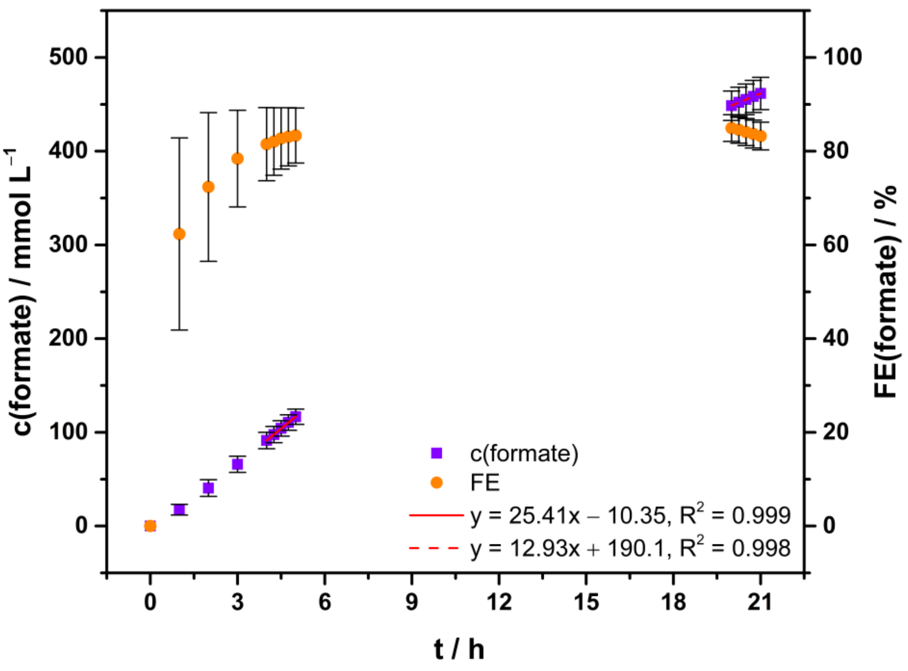

Figure S21: Data for electrolysis (E1, E2, E3), experimental details are provided in section 1.4 and results in Table S11, Table S12 and Table S13.

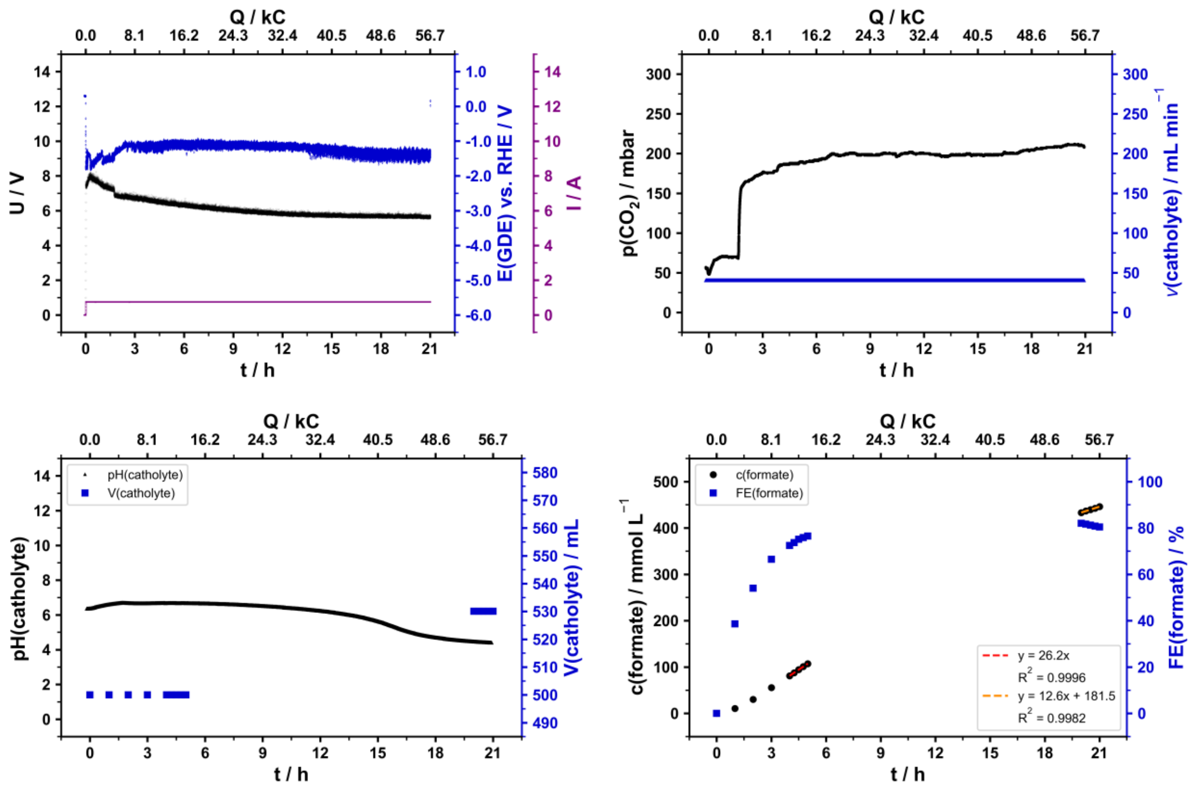

Figure S22: Data for electrolysis (E1), experimental details are provided in section 1.4 and results in Table S12 and Table S13.

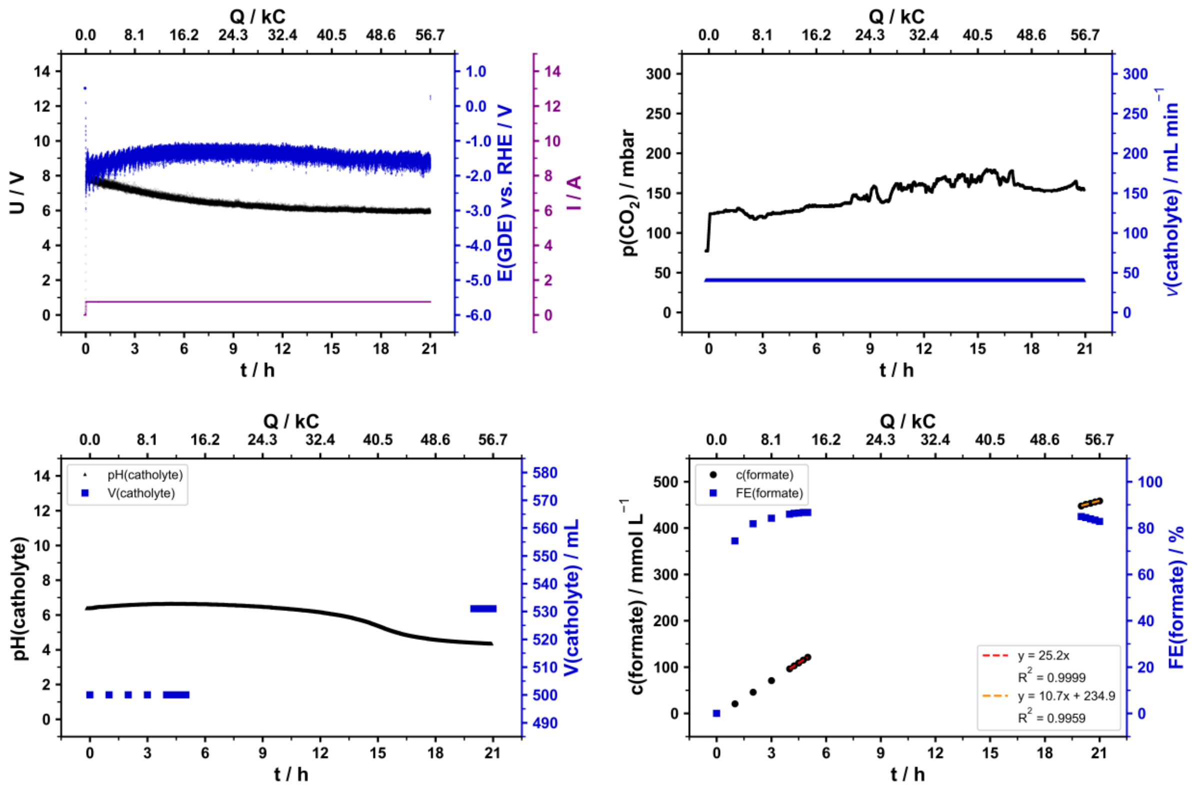

Figure S23: Data for electrolysis (E2), experimental details are provided in section 1.4 and results in Table S12 and Table S13.

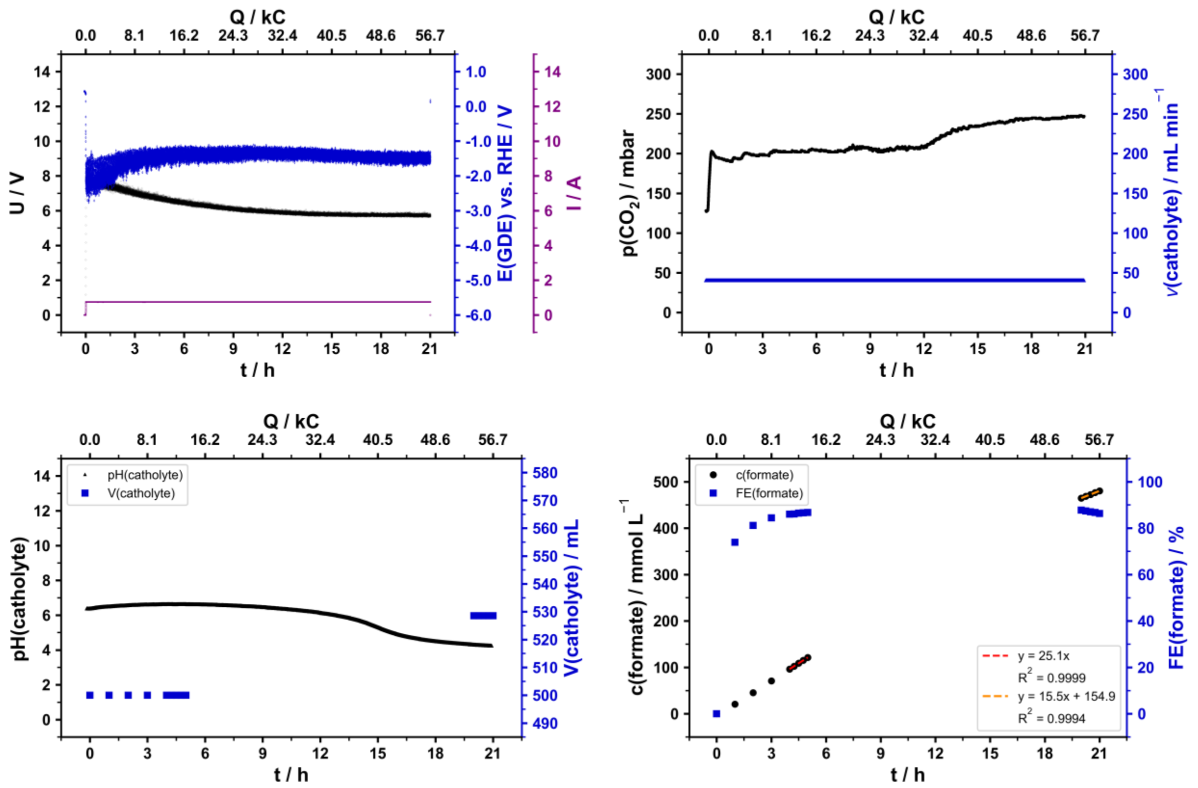

Figure S24: Data for electrolysis (E3), experimental details are provided in section 1.4 and results in Table S12 and Table S13.

3.1.6 Bi_2O_3 GDEs

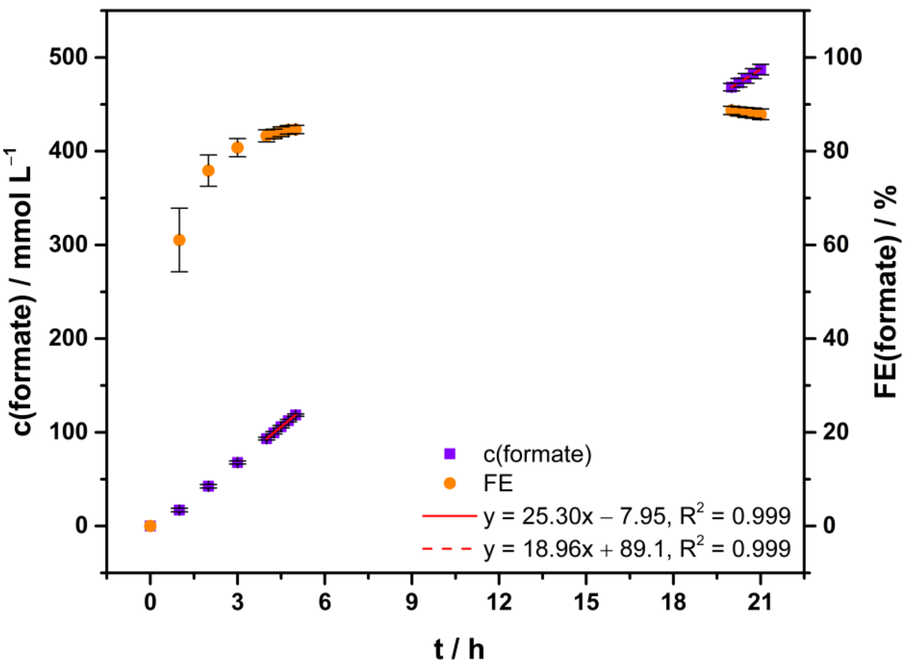

Figure S25: Data for electrolysis (F1, F2, F3), experimental details are provided in section 1.4 and results in Table S11, Table S12 and Table S13.

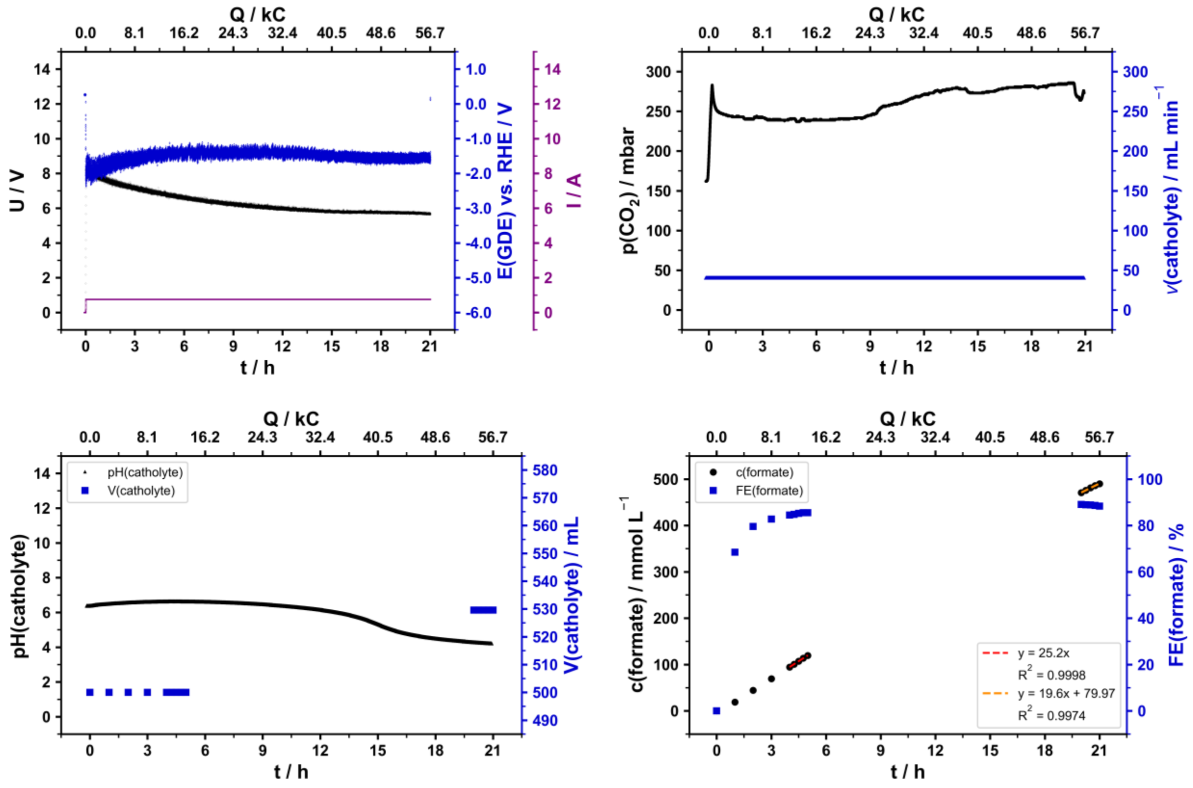

Figure S26: Data for electrolysis (F1), experimental details are provided in section 1.4 and results in Table S12 and Table S13.

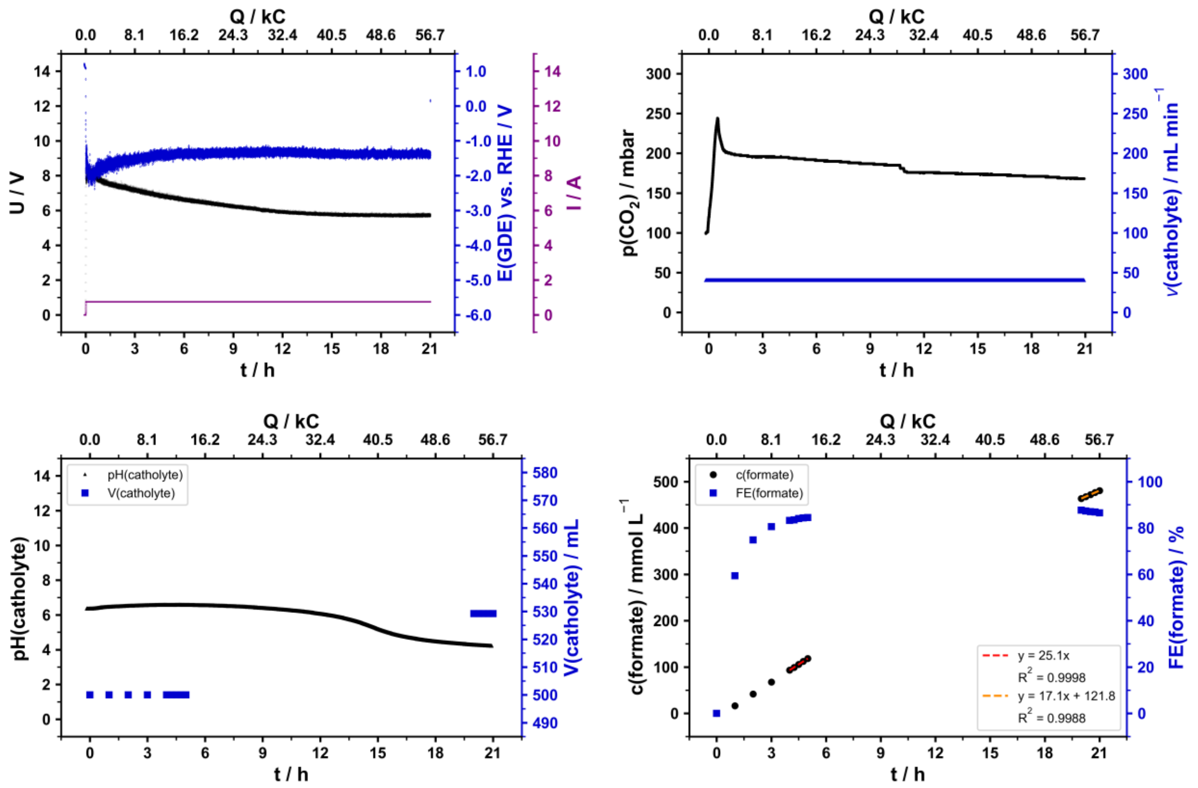

Figure S27: Data for electrolysis (F2), experimental details are provided in section 1.4 and results in Table S12 and Table S13.

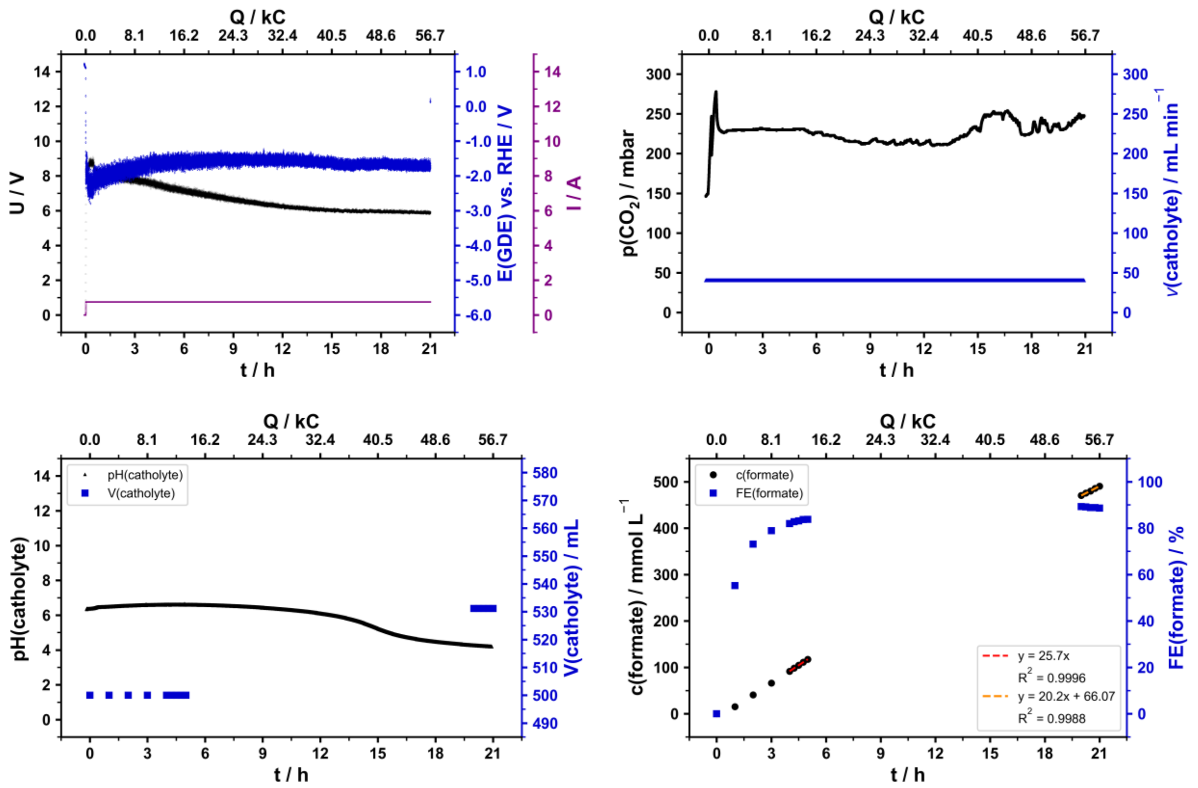

Figure S28: Data for electrolysis (F3), experimental details are provided in section 1.4 and results in Table S12 and Table S13.

3.2 Electrosynthesis of formate at variable current density

Electrolysis results have been summarized in Table S14. Moreover, detailed courses of each individual electrolysis are provided.

Table S14: Overview of volume, formate concentration, formate FE and pH determined *n* = 3 after electrolysis at variable current densities in the catholyte of formate electrosynthesis with 0.2 mol L^{-1} KH_2PO_4 / K_2HPO_4 as starting electrolyte.

Electrolysis	V / mL	c(formate) / mmol L^{-1}	FE(formate) / %	pH
(B4)	507.1 ± 0.4	238.9 ± 0.3	91.44 ± 0.07	6.41 ± 0.05
(B5)	508.7 ± 0.8	236.1 ± 0.4	90.6 ± 0.3	6.42 ± 0.05
(B6)	509.3 ± 1.0	236.9 ± 0.1	91.3 ± 0.2	6.43 ± 0.05
(B7)	527.1 ± 0.4	198.4 ± 0.3	90.8 ± 0.2	6.47 ± 0.05
(B8)	530.4 ± 0.4	199.6 ± 0.4	91.87 ± 0.09	6.47 ± 0.05
(B9)	530.2 ± 0.5	196.4 ± 0.3	90.43 ± 0.04	6.52 ± 0.05
(B10)	518.3 ± 1.3	362.1 ± 0.2	87.3 ± 0.3	5.47 ± 0.05
(B11)	518.4 ± 1.1	354.5 ± 0.2	86.9 ± 0.3	5.57 ± 0.05
(B12)	516.9 ± 0.7	347.2 ± 0.5	83.5 ± 0.3	5.72 ± 0.05

3.2.1 Operation alternating between full and half load

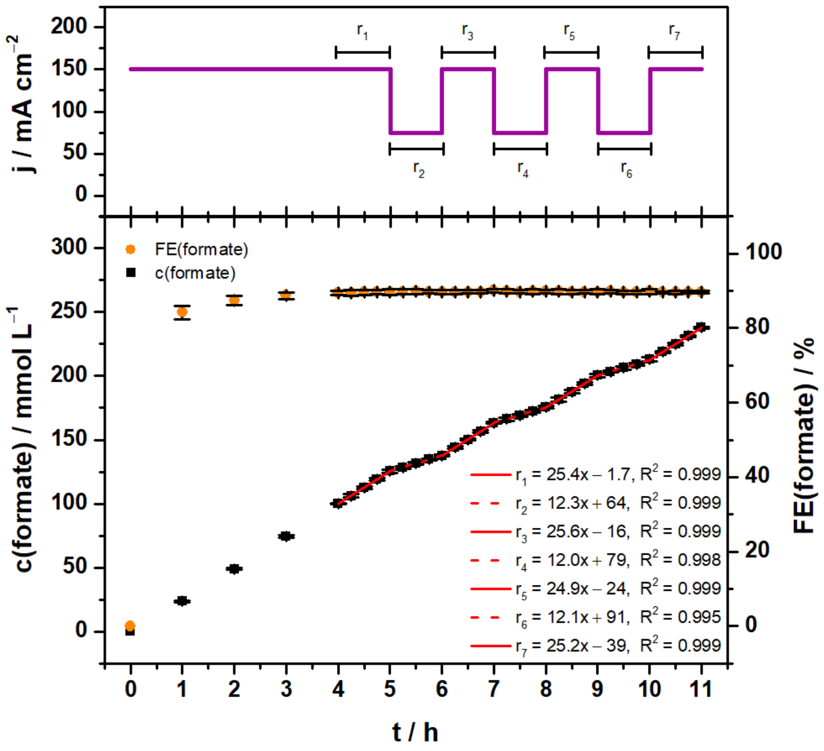

Figure S29: Data for electrolysis (B4, B5, B6), experimental details are provided in section 1.4 and results in Table S14.

Table S15: Synthesis rates of formate for different time intervals at different current densities as shown in Figure S29.

Rate	Interval	Current density / mA cm^{-2}	c(formate) rate / mmol L^{-1} h^{-1}
r_1	4 - 5 h	150	25.43 ± 0.08
r_2	5 - 6 h	75	12.28 ± 0.12
r_3	6 - 7 h	150	25.6 ± 0.3
r_4	7 - 8 h	75	12.0 ± 0.3
r_5	8 - 9 h	150	24.95 ± 0.06
r_6	9 - 10 h	75	12.1 ± 0.5
r_7	10 - 11 h	150	25.2 ± 0.4

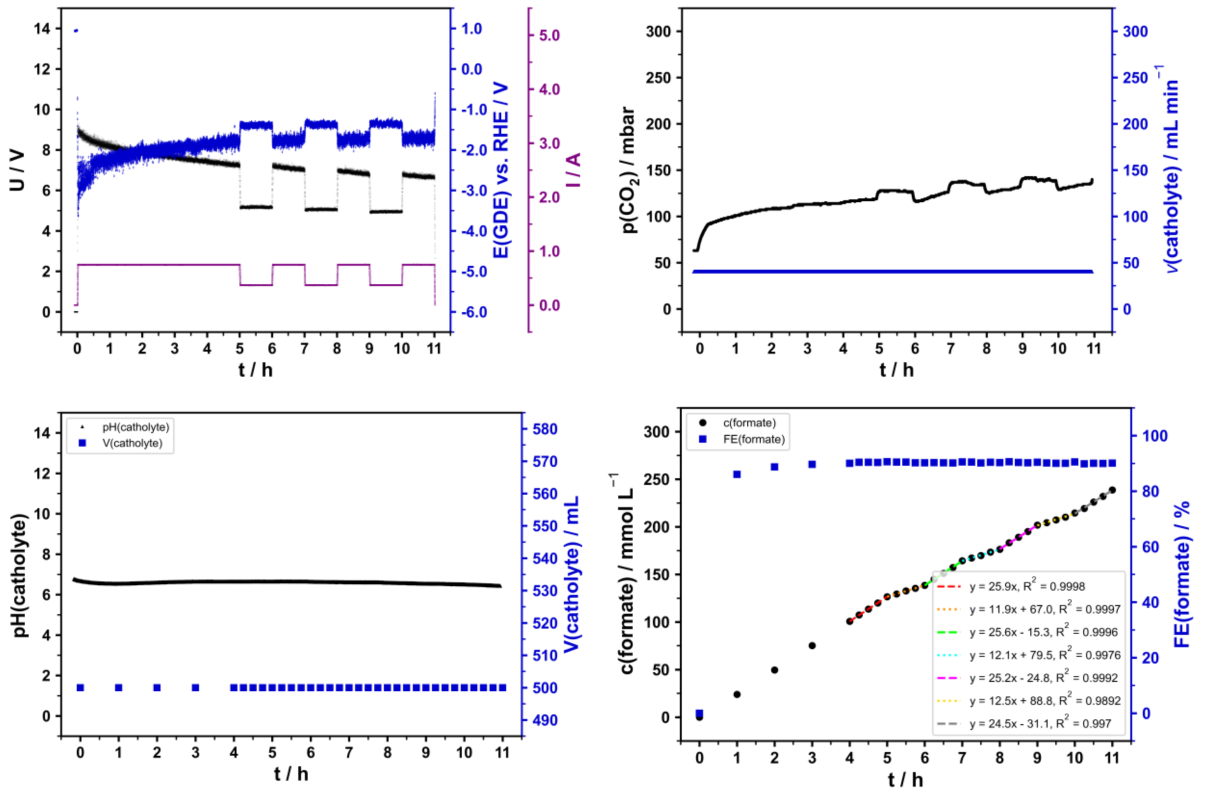

Figure S30: Data for electrolysis (B4), experimental details are provided in section 1.4 and results in Table S14.

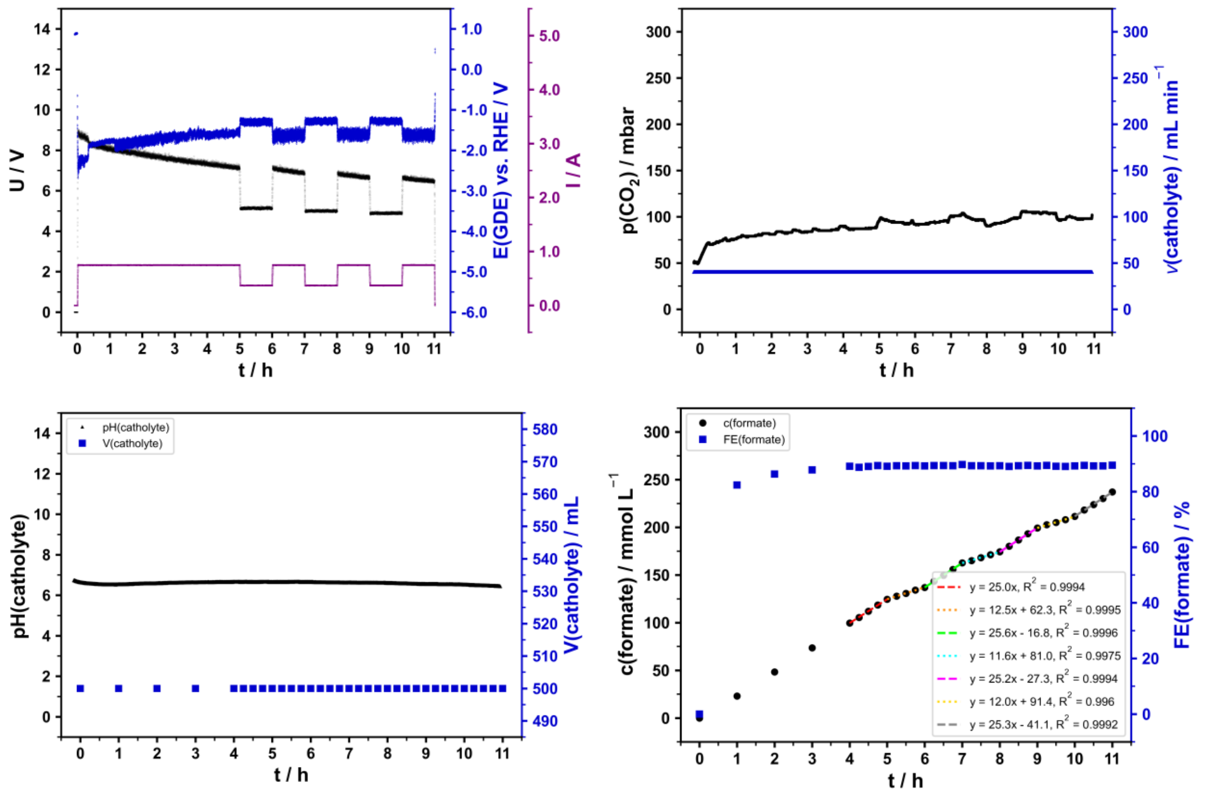

Figure S31: Data for electrolysis (B5), experimental details are provided in section 1.4 and results in Table S14.

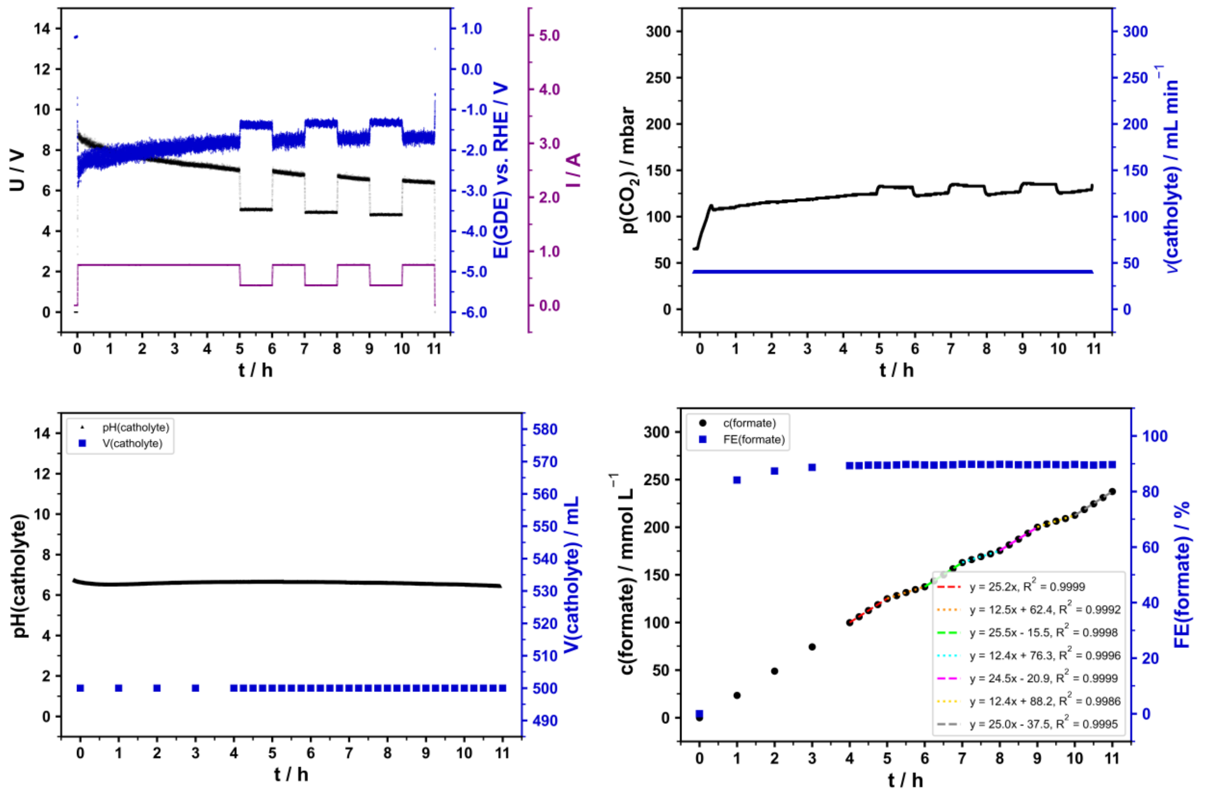

Figure S32: Data for electrolysis (B6), experimental details are provided in section 1.4 and results in Table S14.

3.2.2 Operation alternating between full and zero load

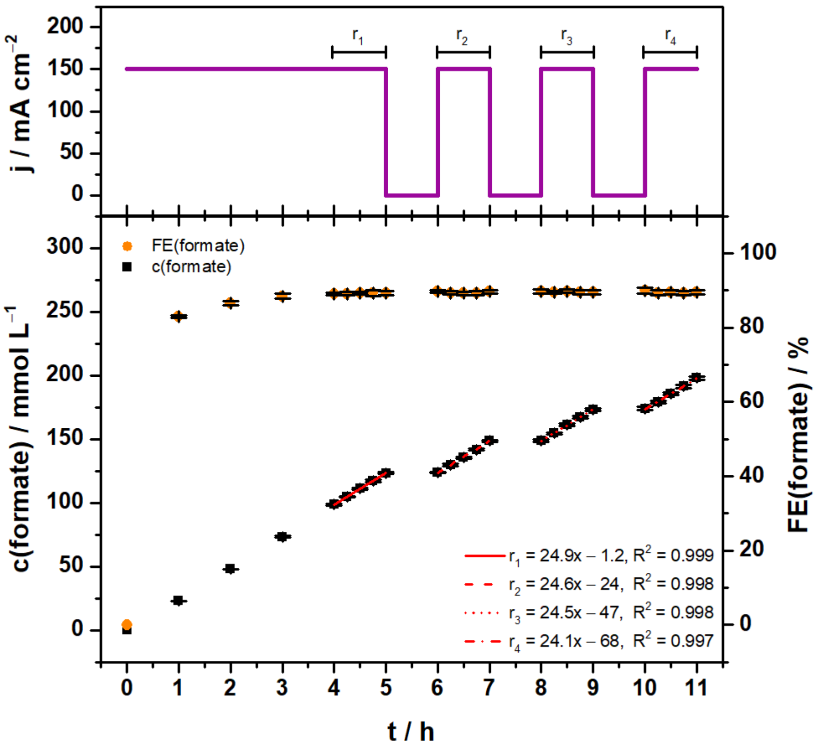

Figure S33: Data for electrolysis (B7, B8, B9), experimental details are provided in section 1.4 and results in Table S14.

Table S16: Synthesis rates of formate for different time intervals at different current densities as shown in Figure S33.

Rate	Interval	Current density / mA cm^{-2}	c(formate) rate / mmol L^{-1} h^{-1}
r_1	4 - 5 h	150	24.93 ± 0.07
r_2	6 - 7 h	150	24.6 ± 0.5
r_3	8 - 9 h	150	24.5 ± 0.4
r_4	10 - 11 h	150	24.1 ± 0.7

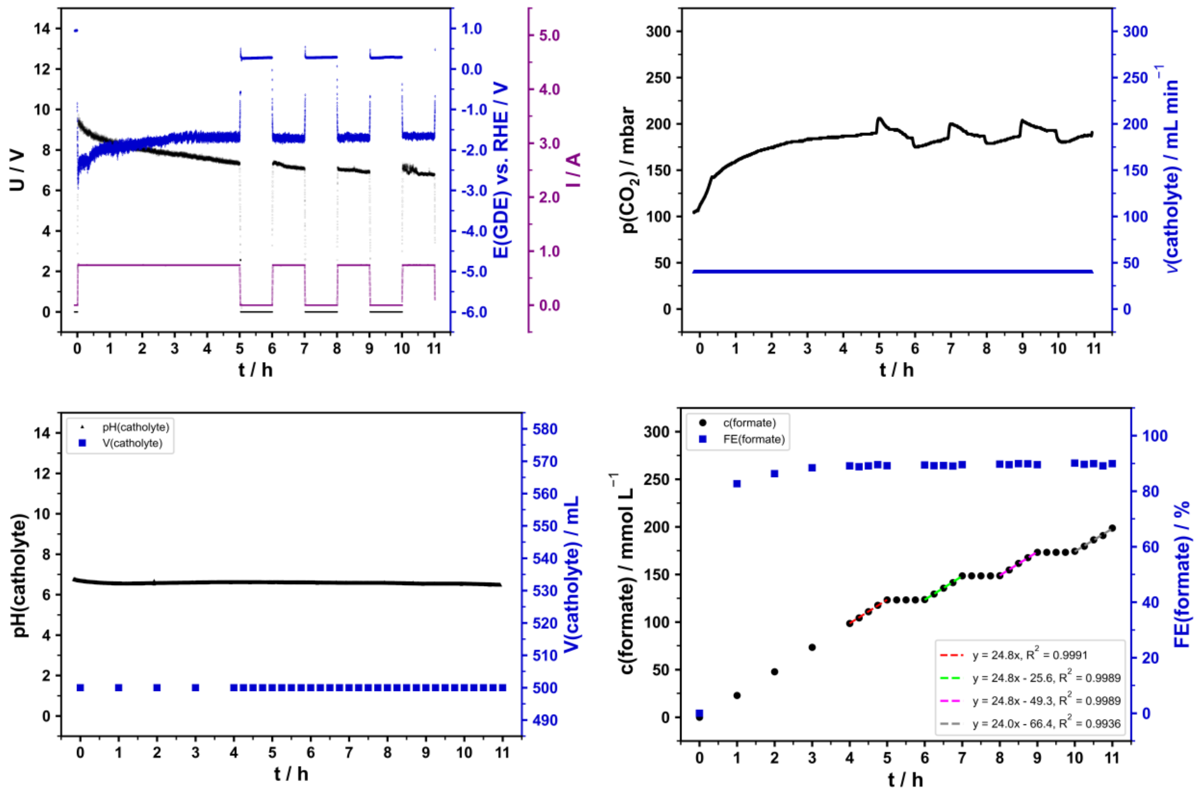

Figure S34: Data for electrolysis (B7), experimental details are provided in section 1.4 and results in Table S14.

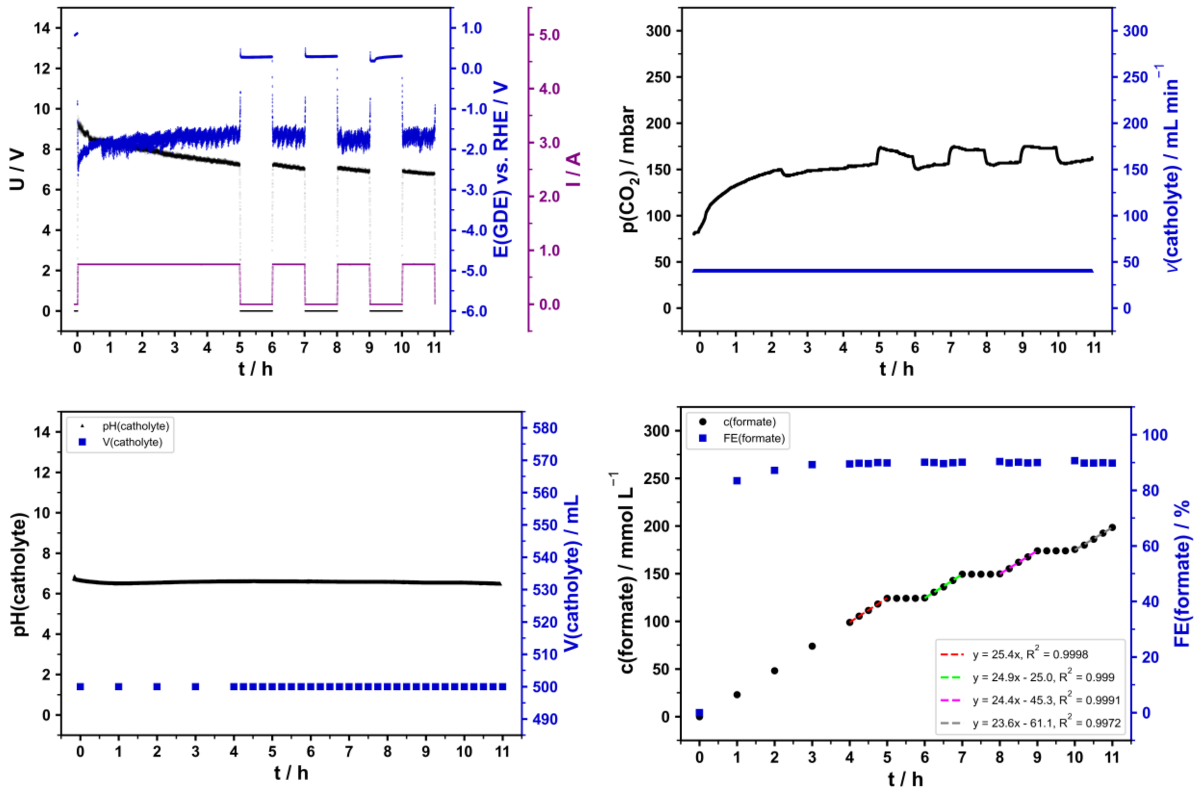

Figure S35: Data for electrolysis (B8), experimental details are provided in section 1.4 and results in Table S14.

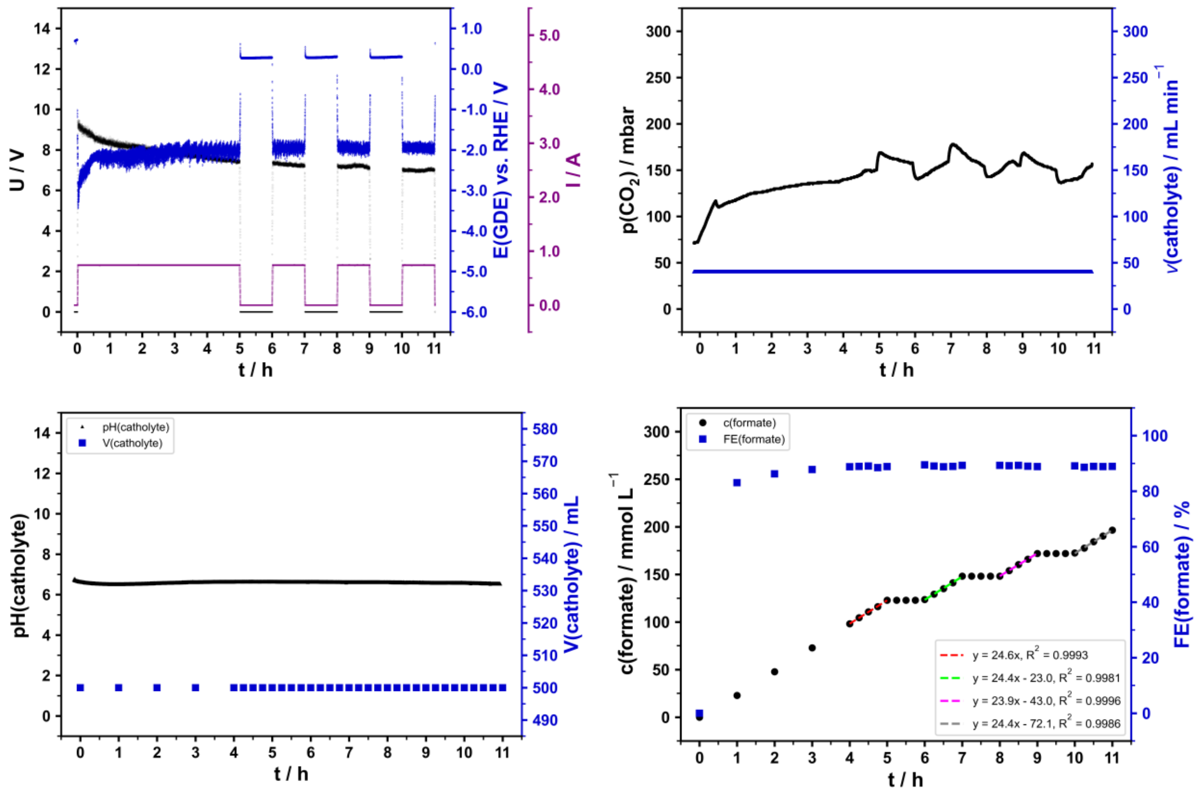

Figure S36: Data for electrolysis (B9), experimental details are provided in section 1.4 and results in Table S14.

3.2.3 Day-night cycle

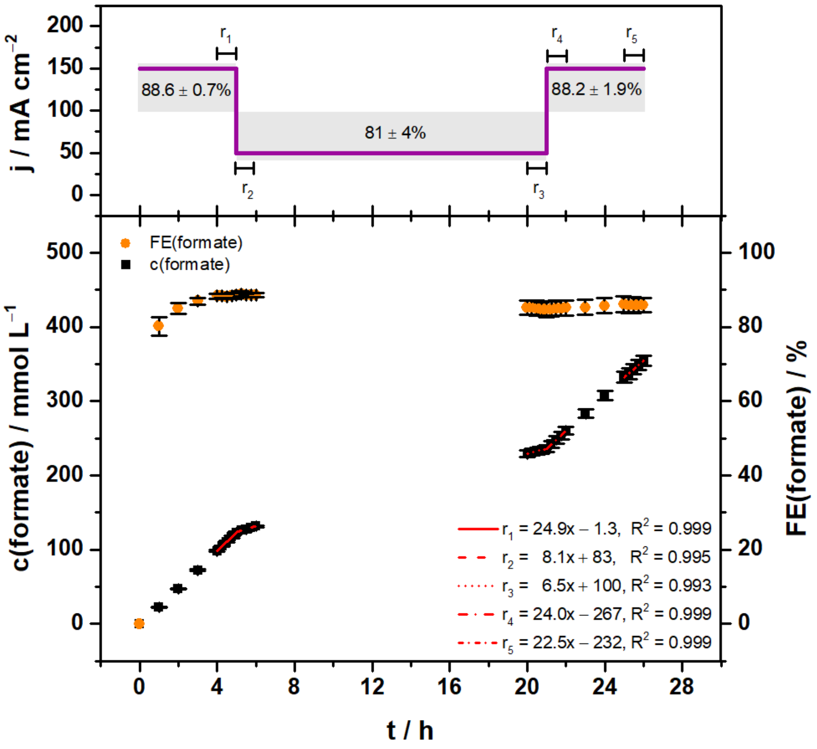

Figure S37: Data for electrolysis (B10, B11, B12), experimental details are provided in section 1.4 and results in Table S14.

Table S17: Synthesis rates of formate for different time intervals at different current densities as shown in Figure S37.

Rate	Interval	Current density / mA cm^{-2}	c(formate) rate / mmol L^{-1} h^{-1}
r_1	4 - 5 h	150	24.93 ± 0.16
r_2	5 - 6 h	50	8.1 ± 0.3
r_3	20 - 21 h	50	6.5 ± 0.3
r_4	21 - 22 h	150	23.97 ± 0.07
r_5	25 - 26 h	150	22.5 ± 0.3

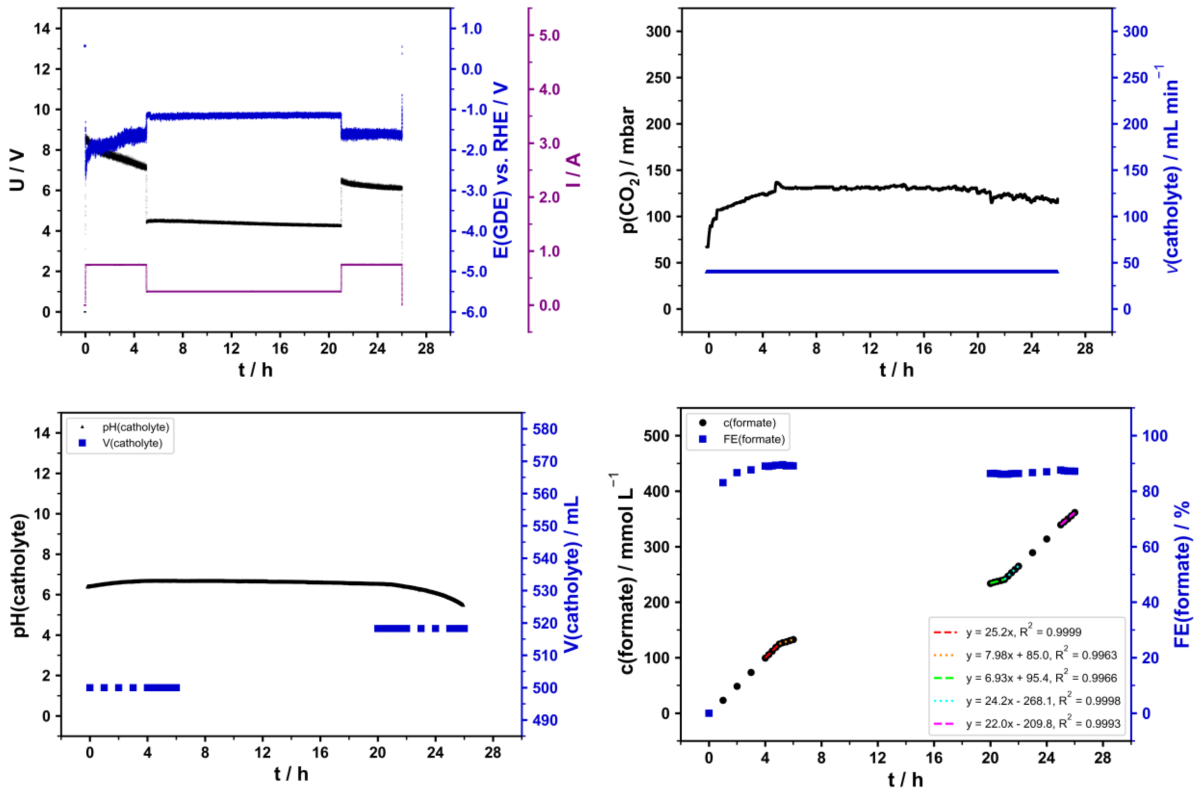

Figure S38: Data for electrolysis (B10), experimental details are provided in section 1.4 and results in Table S14.

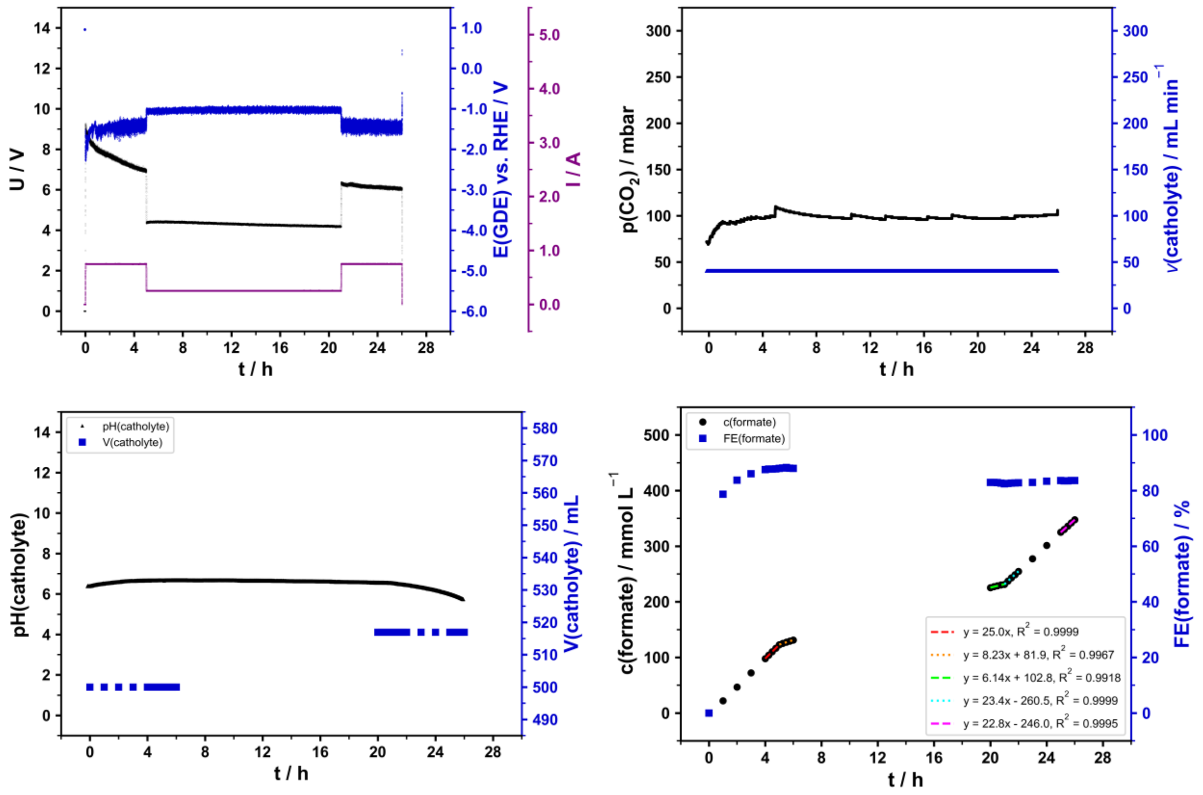

Figure S39: Data for electrolysis (B11), experimental details are provided in section 1.4 and results in Table S14.

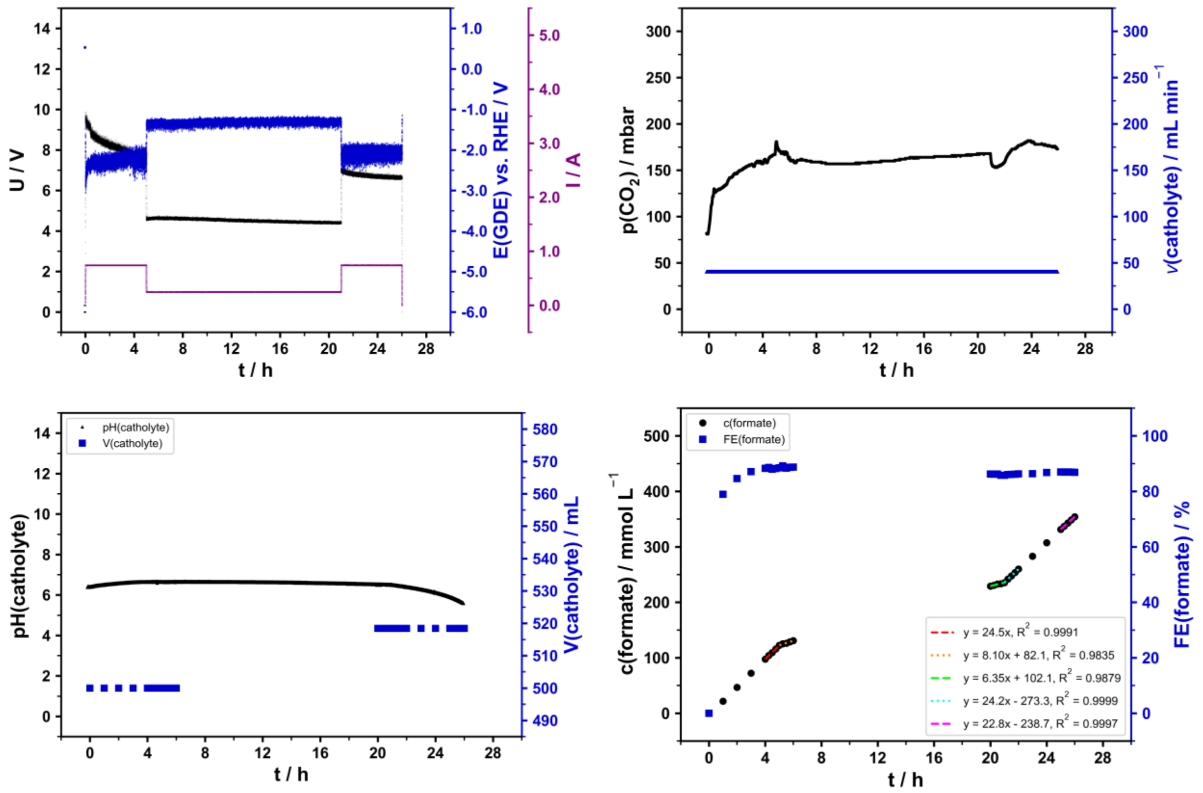

Figure S40: Data for electrolysis (B12), experimental details are provided in section 1.4 and results in Table S14.

3.3 Pictures and cross sections of GDE before and after electrolysis

3.3.1 Bi GDEs

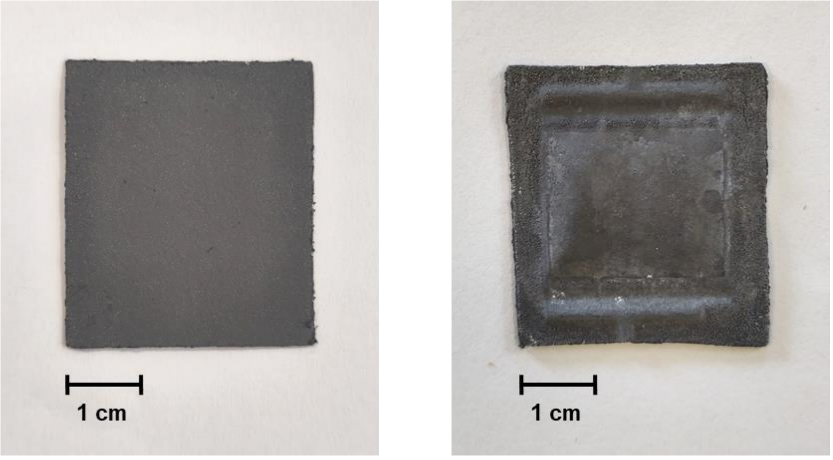

Figure S41: Exemplary pictures of a self-fabricated Bi based GDE (A, cf. Table S3) before (left) and after (right) electrolysis. Details of the fabrication process are provided in section 1.2, details on the electrolysis conditions in section 1.4.

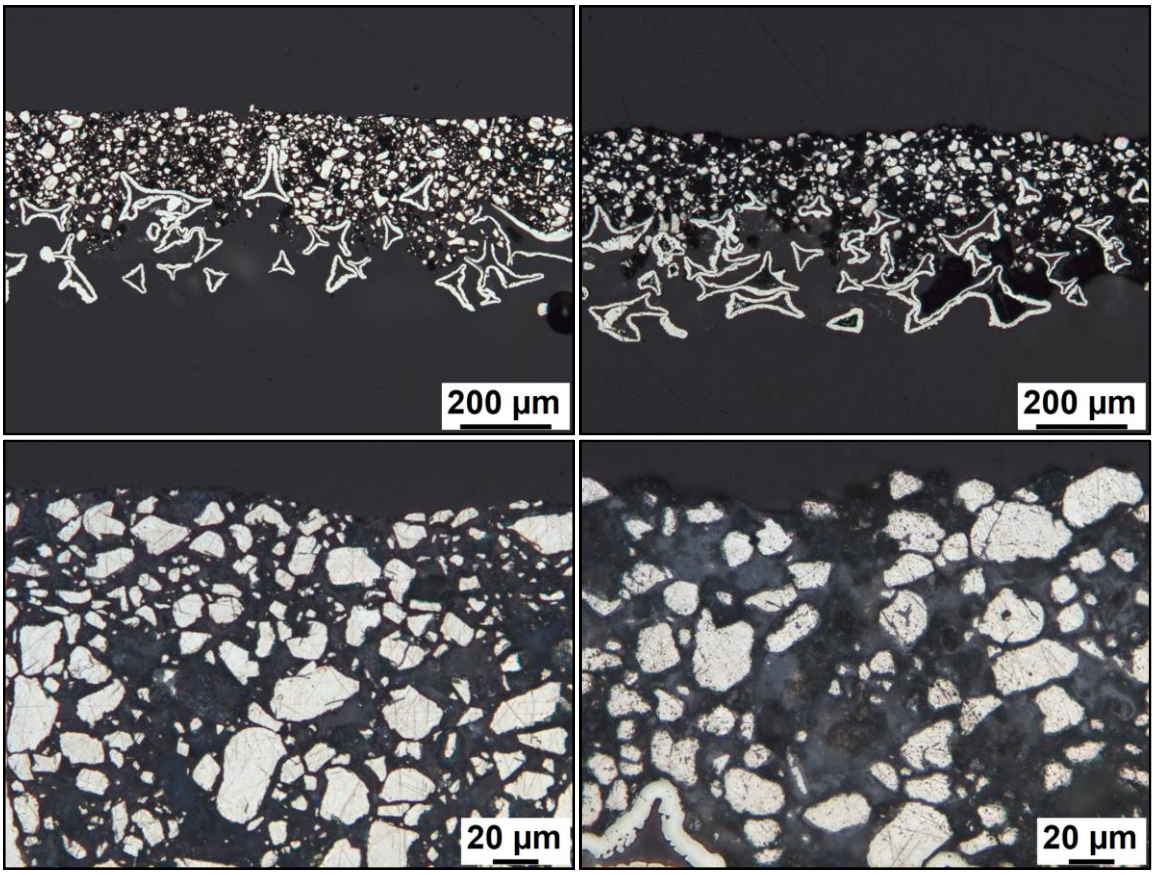

Figure S42: Exemplary cross section of a self-fabricated Bi based GDE (A, cf. Table S3) before (left) and after (right) electrolysis. Details of the fabrication process are provided in section 1.2, details on the electrolysis conditions in section 1.4.

3.3.2 Bi / Bi_2O_3 (80:20) GDEs

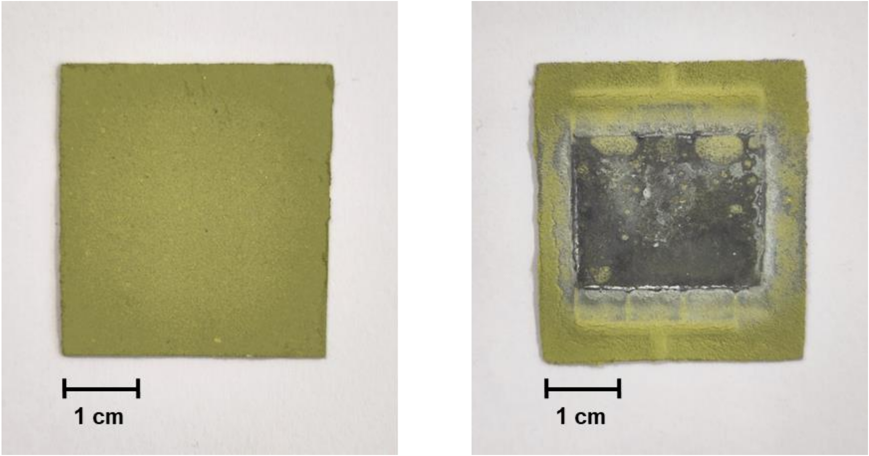

Figure S43: Exemplary pictures of a self-fabricated Bi / Bi_2O_3 (80:20) based GDE (B, cf. Table S3) before (left) and after (right) electrolysis. Details of the fabrication process are provided in section 1.2, details on the electrolysis conditions in section 1.4.

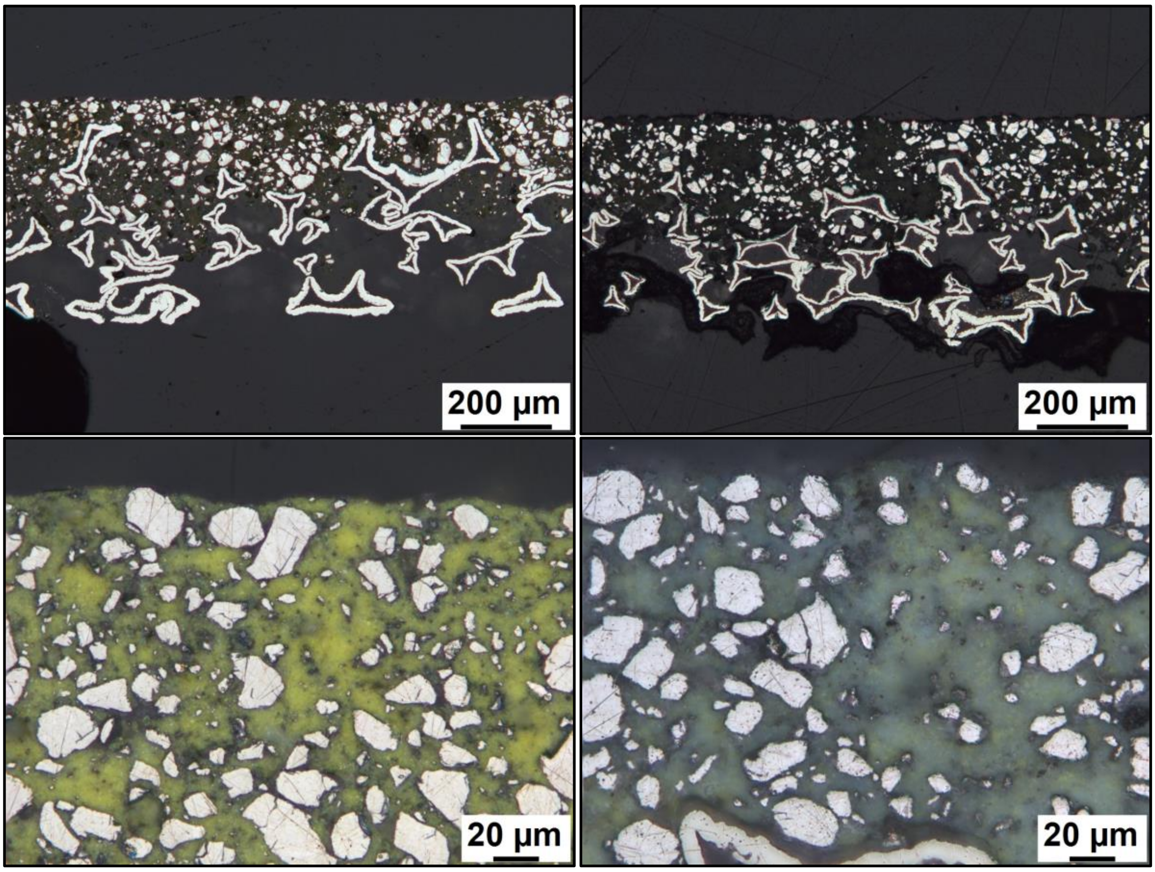

Figure S44: Exemplary cross section of a self-fabricated Bi / Bi_2O_3 (80:20) based GDE (B, cf. Table S3) before (left) and after (right) electrolysis. Details of the fabrication process are provided in section 1.2, details on the electrolysis conditions in section 1.4.

3.3.3 Bi / Bi_2O_3 (60:40) GDEs

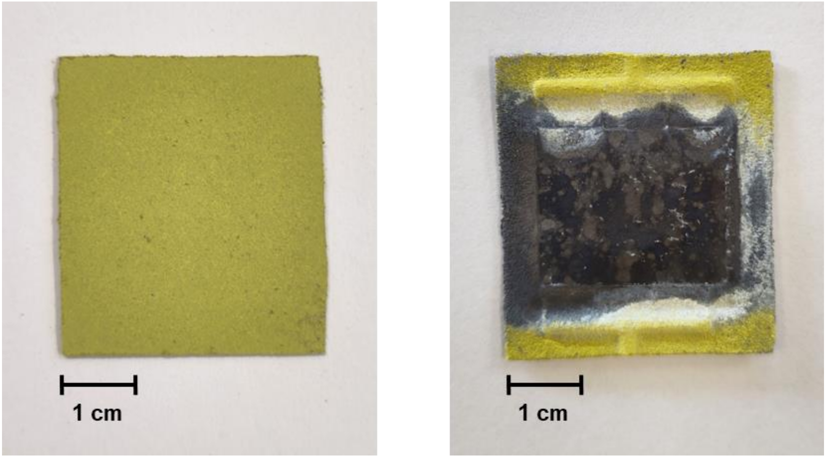

Figure S45: Exemplary pictures of a self-fabricated Bi / Bi_2O_3 (60:40) based GDE (C, cf. Table S3) before (left) and after (right) electrolysis. Details of the fabrication process are provided in section 1.2, details on the electrolysis conditions in section 1.4.

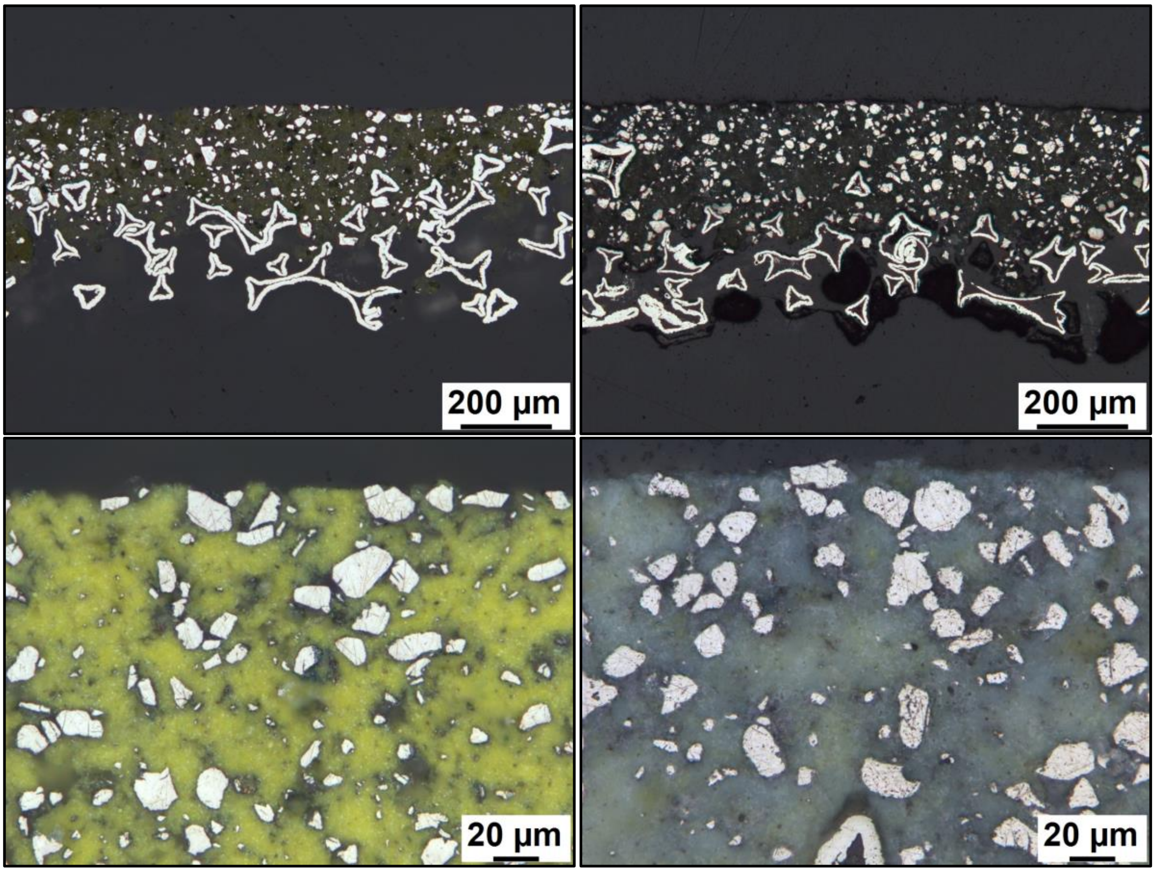

Figure S46: Exemplary cross section of a self-fabricated Bi / Bi_2O_3 (60:40) based GDE (C, cf. Table S3) before (left) and after (right) electrolysis. Details of the fabrication process are provided in section 1.2, details on the electrolysis conditions in section 1.4.

3.3.4 Bi / Bi_2O_3 (40:60) GDEs

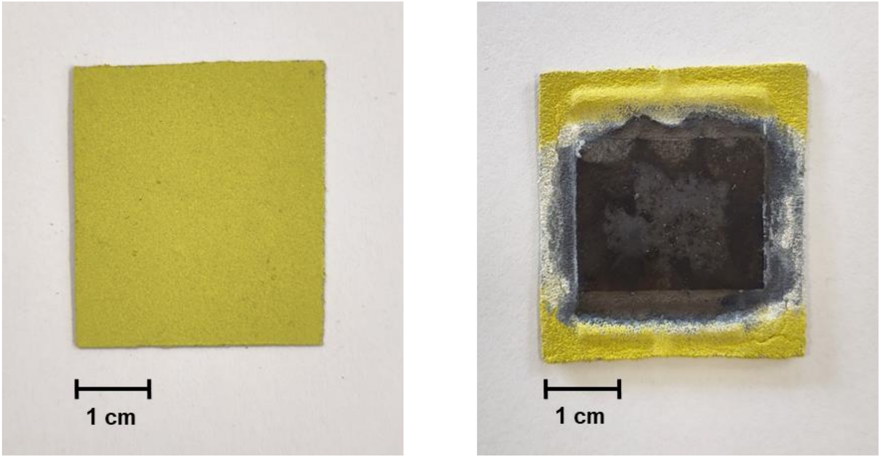

Figure S47: Exemplary pictures of a self-fabricated Bi / Bi_2O_3 (40:60) based GDE (D, cf. Table S3) before (left) and after (right) electrolysis. Details of the fabrication process are provided in section 1.2, details on the electrolysis conditions in section 1.4.

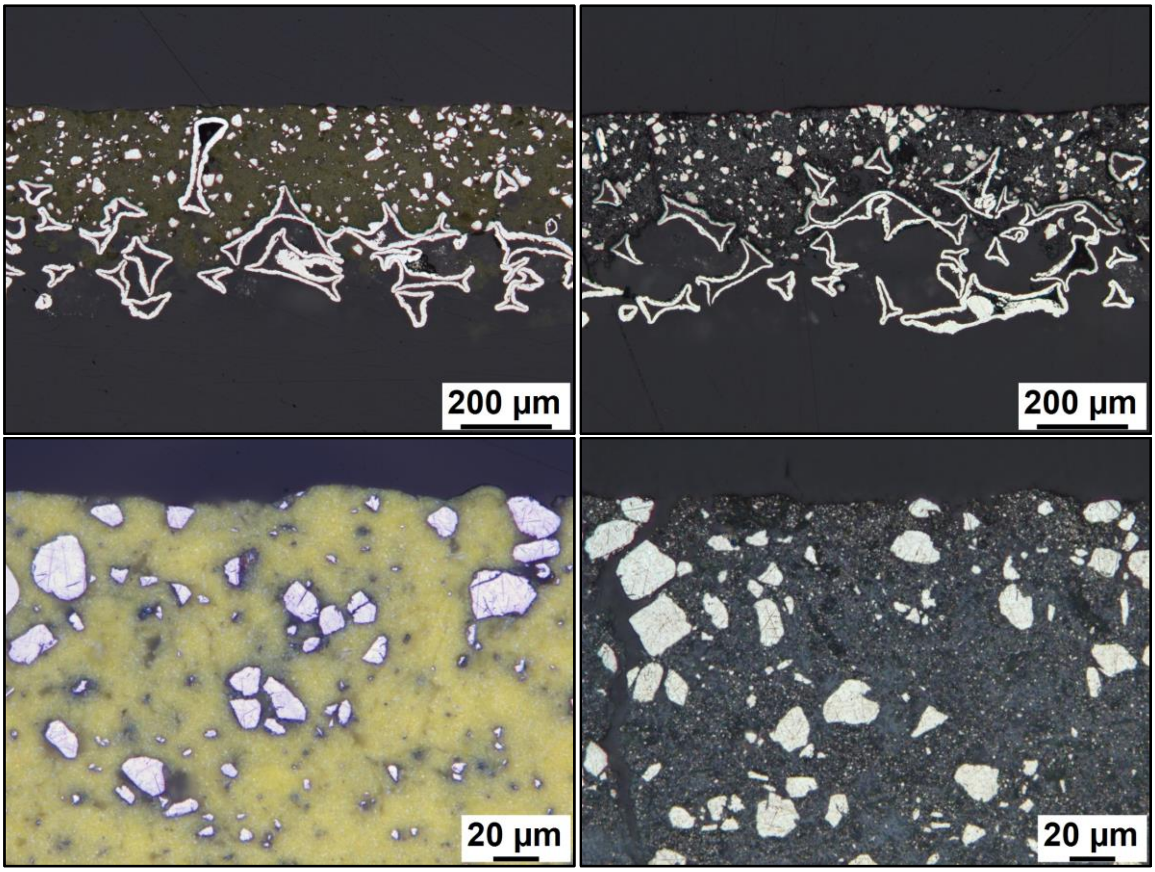

Figure S48: Exemplary cross section of a self-fabricated Bi / Bi_2O_3 (40:60) based GDE (D, cf. Table S3) before (left) and after (right) electrolysis. Details of the fabrication process are provided in section 1.2, details on the electrolysis conditions in section 1.4.

3.3.5 Bi / Bi_2O_3 (20:80) GDEs

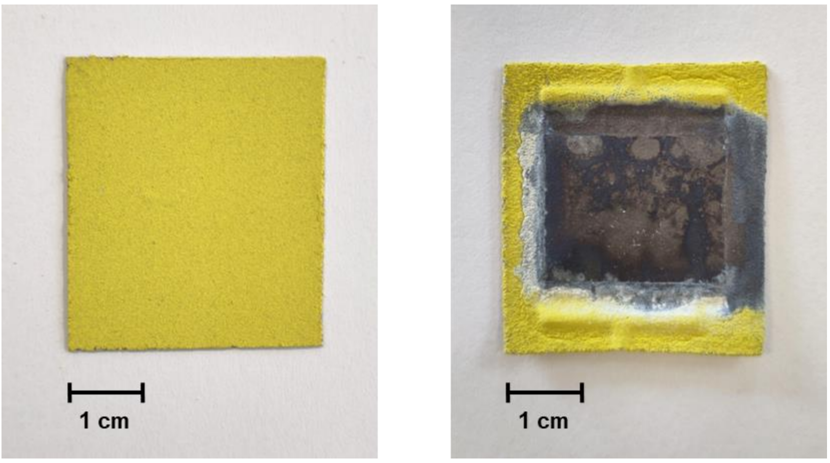

Figure S49: Exemplary pictures of a self-fabricated Bi / Bi_2O_3 (20:80) based GDE (E, cf. Table S3) before (left) and after (right) electrolysis. Details of the fabrication process are provided in section 1.2, details on the electrolysis conditions in section 1.4.

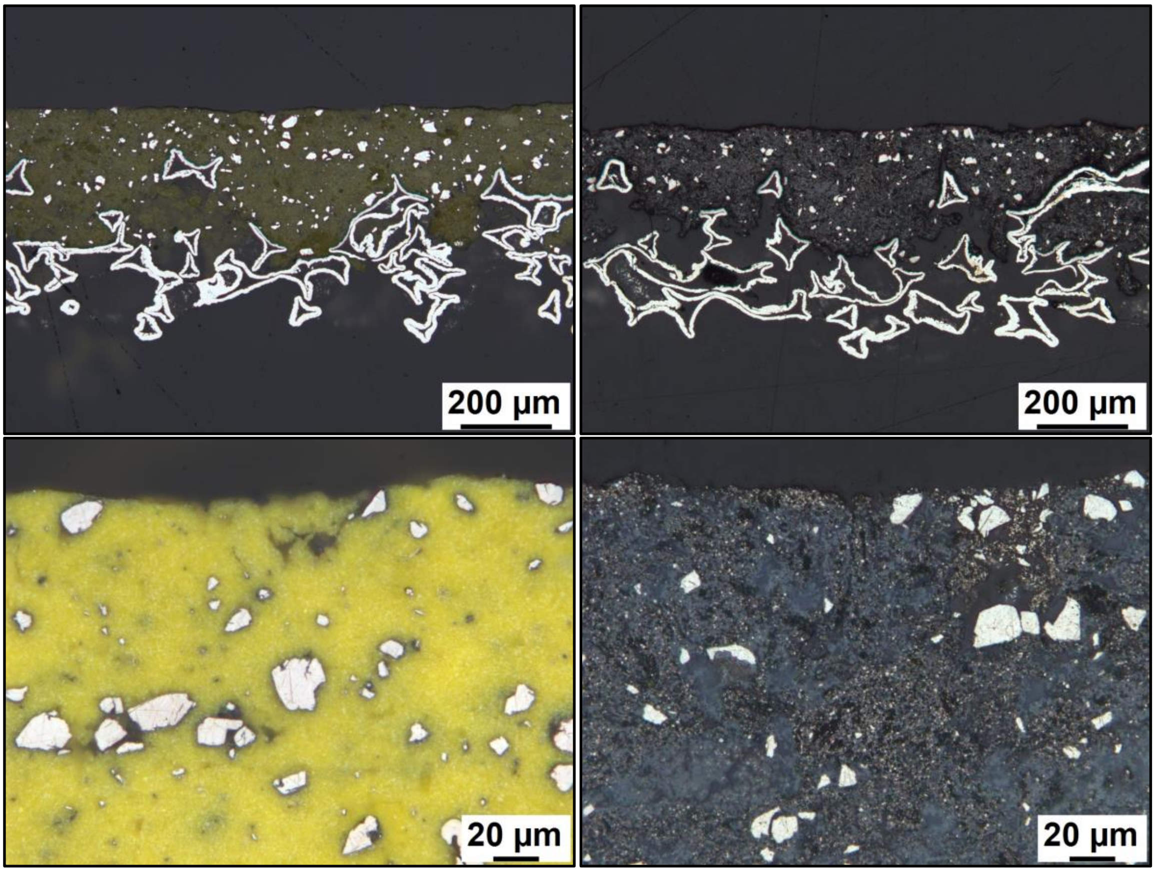

Figure S50: Exemplary cross section of a self-fabricated Bi / Bi_2O_3 (20:80) based GDE (E, cf. Table S3) before (left) and after (right) electrolysis. Details of the fabrication process are provided in section 1.2, details on the electrolysis conditions in section 1.4.

3.3.6 Bi_2O_3 GDEs

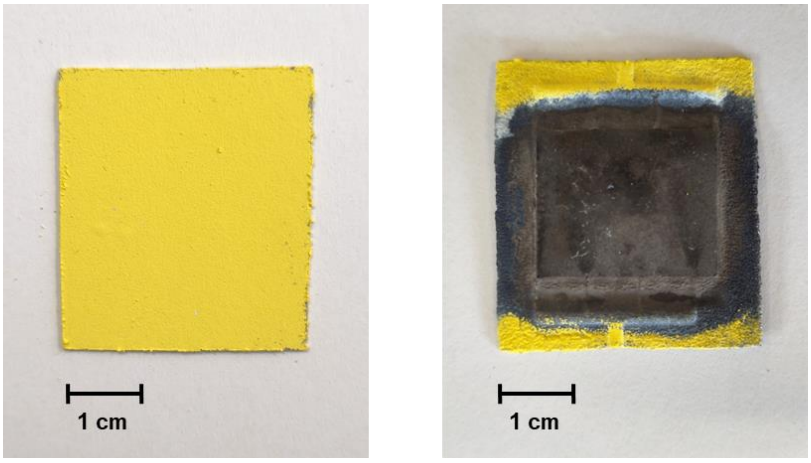

Figure S51: Exemplary pictures of a self-fabricated Bi_2O_3 based GDE (F, cf. Table S3) before (left) and after (right) electrolysis. Details of the fabrication process are provided in section 1.2, details on the electrolysis conditions in section 1.4.

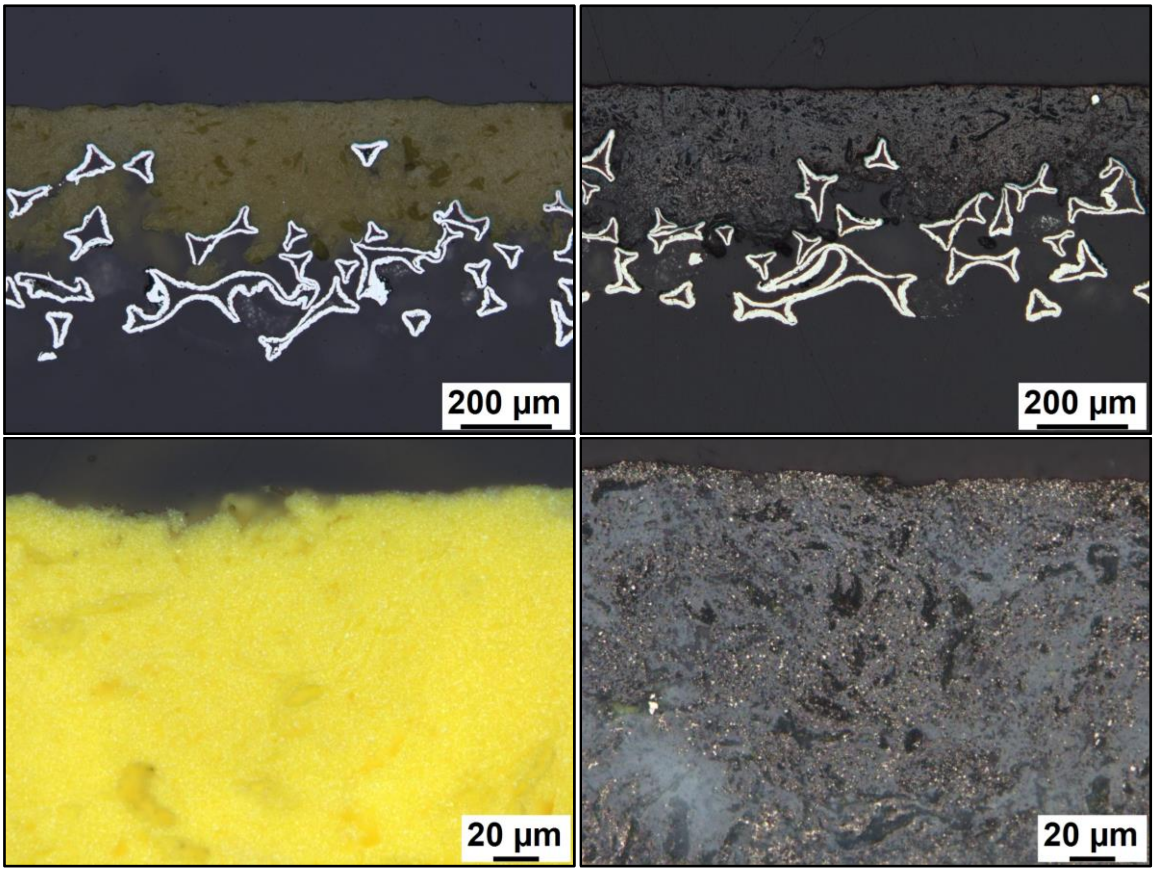

Figure S52: Exemplary cross section of a self-fabricated Bi_2O_3 based GDE (F, cf. Table S3) before (left) and after (right) electrolysis. Details of the fabrication process are provided in section 1.2, details on the electrolysis conditions in section 1.4.
